# Personalized Neoantigen Vaccine plus Regorafenib Increases Rgs2⁺CD8⁺ T Cells Infiltration and Reprograms the Tumor Microenvironment in Microsatellite Stable Colorectal Cancer Liver Metastases

**DOI:** 10.1002/advs.202508040

**Published:** 2025-06-30

**Authors:** Hengkai Chen, Bin Chen, Yuanfeng Yang, Shoufeng Li, Huajun Cai, Zhicheng Zhuang, Yong Wu, Yuan Gao, Yupeng Chen, Xing Liu, Guoxian Guan, Jinfu Zhuang

**Affiliations:** ^1^ Department of Colorectal Surgery The First Affiliated Hospital of Fujian Medical University Fuzhou 350005 China; ^2^ Department of Colorectal Surgery National Regional Medical Center Binhai Campus of the First Affiliated Hospital Fujian Medical University Fuzhou 350212 China; ^3^ Fujian Abdominal Surgery Research Institute The First Affiliated Hospital of Fujian Medical University Fuzhou 350005 China

**Keywords:** microsatellite stable colorectal cancer liver metastasis, neoantigen vaccine, regorafenib, Rgs2^+^CD8^+^ T cell

## Abstract

Microsatellite stable colorectal cancer liver metastases (MSS‐CRLM) resist immune checkpoint inhibitors due to their immunosuppressive tumor microenvironment (TME) and low mutation burden (TMB). A personalized neoantigen vaccine, Neo‐CRCVAS, using whole‐exome and RNA sequencing of murine MSS‐CRC cells, comprising 7 immunogenic neoantigen peptides with Poly(I:C) is developed and combined with regorafenib as a novel therapy (RegoNeo). In MSS‐CRLM mouse models, RegoNeo significantly enhanced tumor regression and survival while establishing durable immune memory. Single‐cell RNA and TCR sequencing revealed that RegoNeo expanded a distinct Rgs2⁺CD8⁺ T cell population with strong cytotoxic activity and TCR clonal expansion. These Rgs2⁺CD8⁺ T cells, enriched for neoantigen‐specific T cells, demonstrated potent tumor‐killing capabilities in both mouse models and patient‐derived organoids. The findings establish RegoNeo as a promising personalized immunotherapy that reprograms the immunosuppressive tumor microenvironment by increasing Rgs2⁺CD8⁺ T cell infiltration, highlighting both the treatment approach and this specific T cell subset as potential therapeutic targets for MSS‐CRLM patients.

## Graphical abstract

This study synthesized a personalized neoantigen vaccine (Neo‐CRCVAS) and combined it with regorafenib, termed RegoNeo. This combination therapy significantly enhanced antitumor efficacy and promoted long‐term tumor‐specific immune memory in microsatellite stable colorectal cancer liver metastasis (MSS‐CRLM) models by increasing neoantigen‐specific Rgs2^+^CD8^+^ T cells infiltration and reprogramming the immunosuppressive tumor microenvironment (TME), providing novel immunotherapy insights for MSS‐CRLM patients.

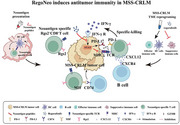

## 1 Introduction

Colorectal cancer (CRC) ranks as the third most prevalent cancer and the second leading cause of cancer‐related deaths worldwide, responsible for more the 935 000 deaths annually.^[^ ^1^ ^]^ Approximately half of CRC patients experience colorectal liver metastases (CRLM) disease progression, making it the leading cause of CRC‐related mortality.^[^ ^2^ ^]^ Despite advancements in systemic chemotherapy, targeted therapies, and surgical resection, the prognosis for CRLM patients remains poor. The 5‐year overall survival (OS) rate for treated patients is 30%–57%, while untreated patients have a median OS less than 12 months.^[^ ^3^ ^]^

Recently, while tumor mutation burden (TMB) has proven inadequate as a predictive marker for immunotherapy efficacy in all solid cancer types,^[^ ^4^ ^]^ checkpoint inhibitors (ICIs) have nonetheless substantially revolutionized cancer treatment, especially in tumors c with high TMB. In CRC, the response to ICIs heavily depends on mismatch repair (MMR) status. Microsatellite instability‐high (MSI‐H) or deficient mismatch repair (dMMR) tumors, with high TMB, account for ≈5%–10% of metastatic CRC (mCRC) cases and demonstrate significant benefit from ICIs, with objective response rates (ORRs) ≈30%–50%, even with monotherapy, and 2‐year OS rates exceeding 60%.^[^ ^5^, ^6^ ^]^ In contrast, microsatellite stable (MSS) or proficient mismatch repair (pMMR) tumors, which account for 90%–95% of metastatic colorectal cancer (mCRC) cases, exhibit minimal responses to ICIs as monotherapy and demonstrate limited efficacy even when treated with a combination of programmed cell death 1 (PD‐1) and cytotoxic T lymphocyte‐associated antigen‐4 (CTLA‐4) antibodies. The ORR for these tumors is generally below 5%, and in the case of MSS‐CRLM, can be as low as 0%. Furthermore, the OS for patients with MSS‐mCRC shows barely any improvement when compared to chemotherapy alone.^[^ ^7^, ^8^ ^]^ The immune resistance of MSS‐CRLM is primarily attributed to its immunosuppressive tumor microenvironment (TME), characterized by a low TMB (with a median number of ≈4–6 Mut Mb^−1^), restricted tumor‐infiltrating lymphocytes (TILs), an abundance of immunosuppressive cells, elevated expression of inhibitory immune checkpoints, and a limited capacity for antigen presentation.^[^ ^9^, ^10^ ^]^ Consequently, strategies aimed at remodeling the TME and enhancing tumor immunogenicity are essential for overcoming immune resistance in MSS‐CRLM.

Neoantigens, originating from tumor‐specific somatic mutations absent in normal tissues, can be presented on tumor cell surfaces via major histocompatibility complex (MHC) molecules and identified as neoepitopes by T cell receptors. Personal vaccines targeting tumor neoantigens can induce tumor‐specific immune responses without causing central tolerance.^[^ ^11^, ^12^ ^]^ Our clinical trials, along with others, have verified that personalized neoantigen vaccines are safe, feasible, and capable of inducing an immune response in melanoma, glioblastoma, non‐small cell lung cancer, and hepatocellular carcinoma.^[^ ^13^, ^14^, ^15^, ^16^ ^]^ As for CRC, recent studies have also demonstrated that the neoantigen burden has the potential to accurately indicate the patients’ prognosis and a subset of these neoantigens may prove to be highly effective targets for CRC immunotherapy.^[^ ^17^, ^18^ ^]^ Preliminary studies suggest that neoantigen vaccines can induce tumor‐specific immune responses even in MSS‐CRC.^[^ ^19^, ^20^ ^]^ Nevertheless, the immunosuppressive TME in MSS‐CRLM limits the efficacy of neoantigen vaccines alone. Therefore, combining neoantigen vaccines with agents that can reprogram the TME offers a promising strategy.

Tyrosine kinase inhibitors (TKIs) could both induce tumor vascular normalization, thereby enhancing the infiltration of effector immune cells and stimulating effector immune cells and dendritic cells (DCs) while concurrently suppressing immunosuppressive cells, leading to immunomodulation.^[^ ^21^ ^]^ Among them, regorafenib was the only TKI that could reprogram the immunosuppressive TME by rescuing the upregulation of immune checkpoints including programmed cell death ligand 1 (PD‐L1) mediated by INF‐γ through the STAT pathway, while also upregulating MHC expression in both tumor cells and immune cells.^[^ ^22^ ^]^ Consequently, these multifaceted mechanisms contribute to the inhibition of tumor cell growth and proliferation.^[^ ^21^, ^23^ ^]^ However, the efficacy of regorafenib combined with traditional immunotherapy based on ICIs is hindered by the limited presence of tumor‐specific TILs in TME, thereby restricting the ORR of patients with MSS‐CRLM (ranging from 0%–8.7%), highlighting the need for optimized combinatorial approaches.^[^ ^24^, ^25^, ^26^ ^]^ Considering the broad potential of personalized neoantigen vaccines to trigger neoantigen‐specific T cells, it is plausible that combining this therapy with regorafenib could improve the ORR and long‐term effectiveness of immunotherapy in MSS‐CRLM by inducing tumor‐specific TILs and reprogramming TME through multi‐mechanisms.

**Scheme 1:**
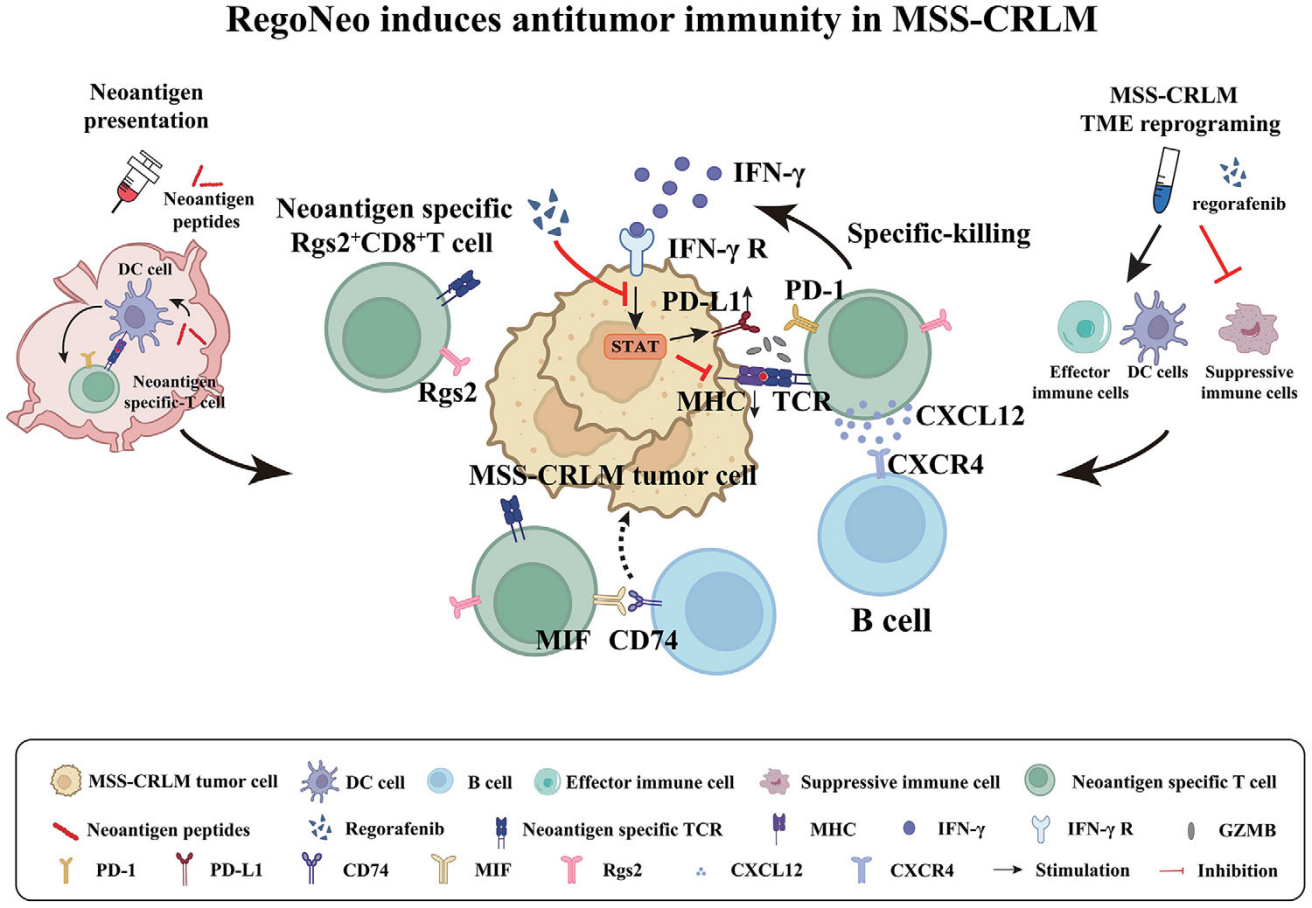
The mechanisms of RegoNeo induing antitumor immunity in MSS‐CRLM. Personalized neoantigen vaccine elicits neoantigen‐specific T cells (left); Regorafenib reprograms the immunosuppressive TME (right); Rgs2^+^CD8^+^ T cells recruit B cells through the CXCL12/CXCR4 axis and facilitate their collaboration via the MIF/CD74 pathway, thereby enhancing tumor‐specific immunity (middle).

In this study, we developed a personalized neoantigen peptide vaccine for the murine MSS‐CRC cell line (Neo‐CRCVAS) with safety, feasibility, and efficacy. Additionally, a Neo‐CRCVAS/regorafenib combination therapy (ReoNeo) was developed to enhance long‐term antitumor efficacy in a murine MSS‐CRLM model. Additionally, a comprehensive multi‐omics study was performed to explore the TME dynamics and potential immune responses after ReoNeo treatment. In summary, this study provides compelling evidence supporting the efficacy of personalized neoantigen therapy in combination with regorafenib for the treatment of MSS‐CRLM and reveals the potential role and mechanisms of Rgs2^+^CD8^+^ T cells as a promising target for MSS‐CRLM immunotherapy (**Scheme** **1**).

## 2 Results

### 2.1 Identification and Screening of Neoantigens

**Figure 1:**
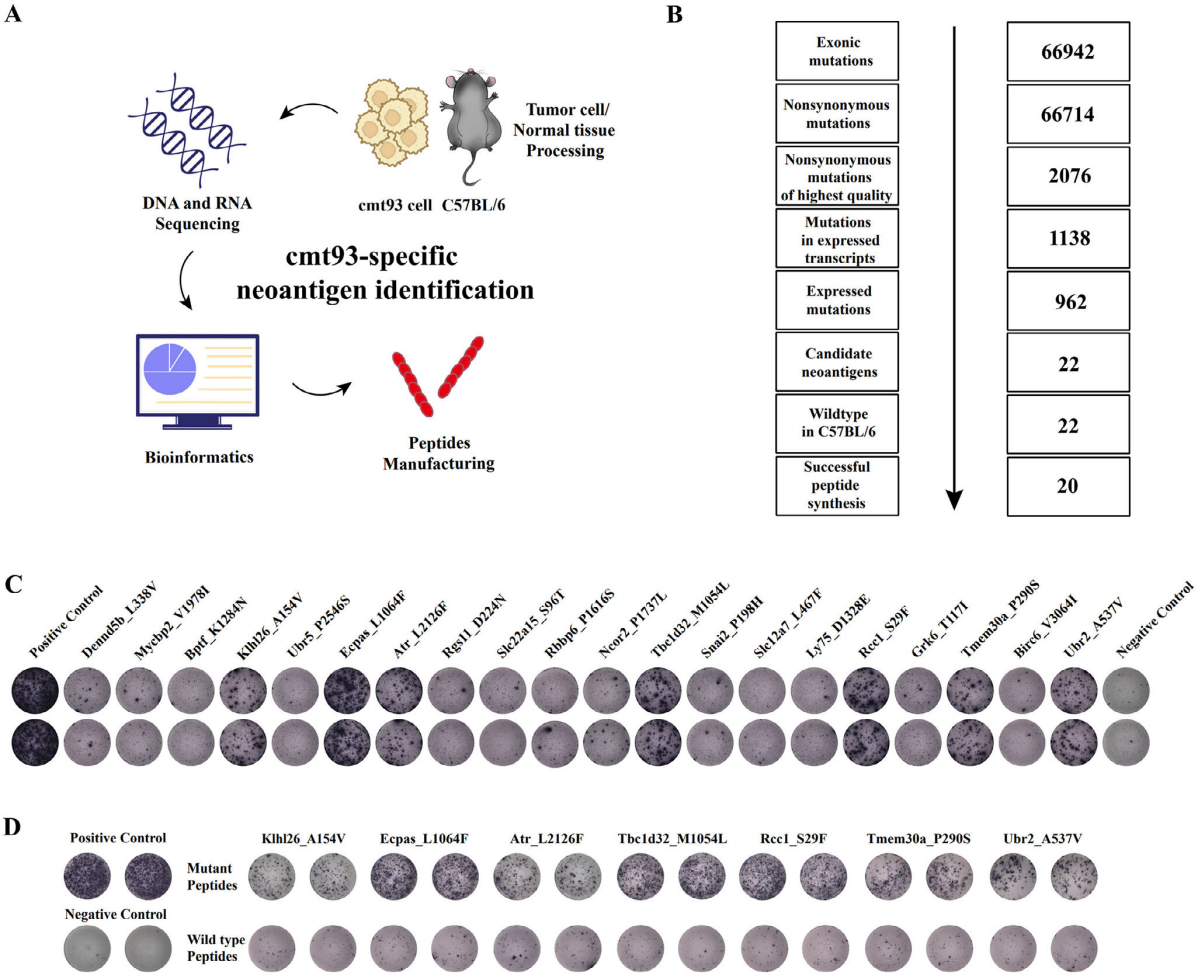
Neoantigen identification and immunogenicity validation. A) Identification process of tumor neoepitope in murine MSS CRC cell line cmt93 cells. B) Neoantigen peptide screening workflow. C) ELISPOT validation of neoantigen immunogenicity. D) Cross‐reactivity analysis of seven neoantigen peptides with wild‐type counterparts.

Whole‐exome sequencing (WES) and transcriptome sequencing (RNA‐seq) were performed on murine MSS‐CRC cmt93 cells and C57BL/6 normal tissues to identify potential neoantigen peptides, followed by bioinformatics algorithms for analysis (**Figure** **1**A). According to Figure 1B, VarScan identified a total of 66714 qualified nonsynonymous mutations in whole exome data with a variant allele frequency (VAF) ≥ 10% (Table S1, Supporting Information); Integration with RNA‐seq data showed that 962 of those exhibited consistent expression levels, with a kilobase per million mapped reads (TPM) ≥1 for the corresponding gene expression. In order to identify potentially immunogenic neoantigens, 22 mutated peptides were found to have high binding affinity (IC50 <500 nM) to H‐2K^b^ alleles in cmt93 cells and C57BL/6 mice using NetMHCpan (Table S2, Supporting Information). Subsequently, the peptides containing mutations from 20 neoantigens with higher immunogenicity (IC50<500 nM to H‐2K^b^) were synthesized for the development of a neoantigen vaccine (Figure 1B). Next, twenty neoantigen peptides were split into two groups randomly and injected subcutaneously with Poly(I:C) into the lateral flank of male C57BL/6 mice on days 0, 4, and 8. Following the initial injection, the mice were euthanized after 15 days, and splenic T cells were subsequently collected for immune analysis. The ex vivo IFN‐γ ELISPOT assay (Figure 1C) demonstrated that 7 out of 20 mutated peptides (Klhl26_A154V, Ecpas_L1064F, Atr_L2126F, Tbc1d32_M1054L, Rcc1_S29F, Tmem30a_P290S, Ubr2_A537V) elicited strong immune responses in immunized mice by activating their autologous splenic T cells with neoantigen peptide‐loaded autologous matured dendritic cells (DCs), with no observed cross‐reactivity to the corresponding wild‐type peptide (Figure 1D). Consequently, the seven peptides that exhibited a significant immune response in vivo were chosen as components of the cmt93 neoantigen vaccine. Building on prior evidence that the toll‐like receptor 3 (TLR3) agonist Poly(I:C) is an effective adjuvant for neoantigen‐peptide vaccines in digestive system immunotherapy,^[^ ^27^ ^]^ we developed MSS‐CRC neoantigen vaccines (Neo‐CRCVAS) comprising seven neoantigen peptides (10 µg/peptide) combined with Poly(I:C) for further study.

### 2.2 Neo‐CRCVAS Displays Neoantigen‐Specific Antitumor Efficacy in MSS‐CRLM Mice Model

**Figure 2:**
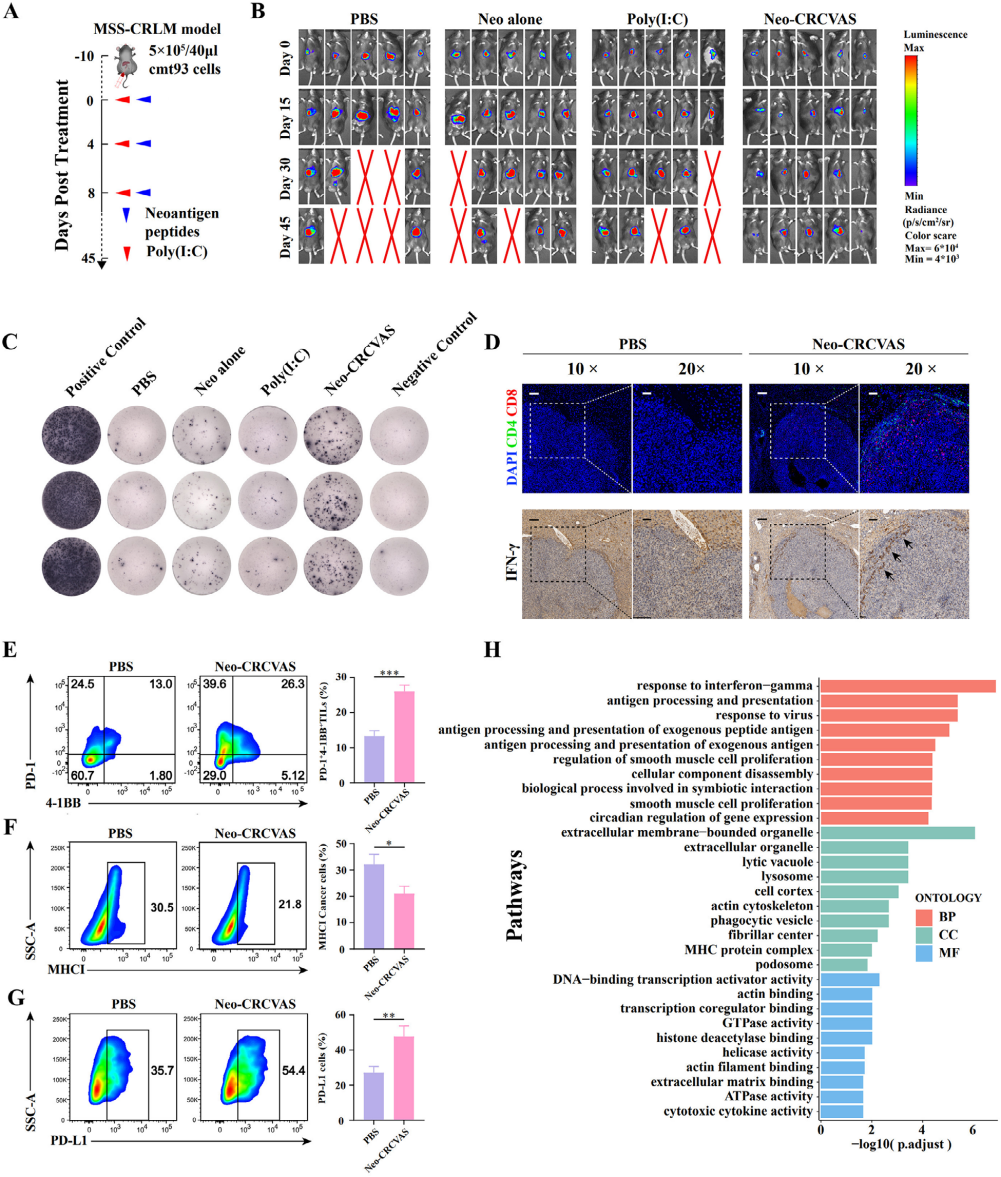
Antitumor efficacy and potential resistant mechanism of Neo‐CRCVAS in MSS‐CRLM mice model. A) Treatment timeline for Neo‐CRCVAS in MSS‐CRLM mice model. B) Tumor burden monitoring of PBS, neoantigen peptides alone, Poly(I:C) alone and Neo‐CRCVAS treated mice by bioluminescence imaging (*n* = 5). C) ELISPOT assay showing neoantigen specific‐reactivity of splenic T cells against seven neoantigen peptides in mice treated with PBS, neoantigen peptides alone, Poly(I:C) alone and Neo‐CRCVAS. D) The representative immunofluorescence image of CD4^+^ and CD8^+^ T cell infiltration in tumor tissues and the representative immunohistochemistry image of IFN‐γ at each treated group, Scare bars, 100 µm (10×), 50 µm (20×). E) Flow cytometry showing the percentage of infiltrating CD8^+^ T cells expressing PD‐1 and 4‐1BB (*n* = 3). F) Flow cytometry analysis showing the percentage of tumor cells expressing MHC1 (*n* = 3). G) Flow cytometry showing the percentage of tumor cells expressing PD‐L1 (*n* = 3). H) Top genes downregulated in biological process (BP), (C) cellular comparison (CC), and (D) molecular function (MF) in the GOALL enrichment analysis in tumor treated with Neo‐CRCVAS compared with tumor treated with PBS. The statistical analysis was performed with ANOVA analysis. Results are shown as mean ± SD. *p < 0.05; **p < 0.01; ***p < 0.001; ****p < 0.0001.

To assess the anti‐tumor effectiveness of Neo‐CRCVAS, an orthotopic MSS‐CRLM mouse model was developed by orthotopically injecting 3 × 10^5^ cmt93 cells from a superior mesenteric vein (SMV), and Neo‐CRCVAS was administered on days 0, 4, and 8 (**Figure** **2**A). Figure 2B and Figure S1A, Supporting Information demonstrated that 15 days post‐neoantigen vaccination, the Neo‐CRCVAS treated group exhibited significant tumor regression (3/5, 60%) compared to the group treated with PBS, Poly(I:C) or neoantigen peptide alone (Neo‐CRCVAS alone). Moreover, during the 45‐day observation period, 40% of Neo‐CRCVAS‐treated mice (2/5) remained a low tumor burden and 100% survived, while 60%, 40%, and 40% were observed dead in the PBS, Poly(I:C), and Neo‐CRCVAS alone‐treated mice, respectively (Figure 2B). Next, we proceeded to investigate the ability of Neo‐CRCVAS to induce neoantigen‐specific reactivity by ELISPOT at day 15. As expected, the Neo‐CRCVAS group demonstrated strong neoantigen‐specific responses of splenic T cells to seven neoantigen peptides, whereas the PBS, Poly(I:C), and Neo‐CRCVAS alone groups showed minimal immunogenicity to the neoepitopes (Figure 2C; Figure S1B, Supporting Information).

Although Neo‐CRCVAS treatment alone reduced tumor growth, but did not completely suppress tumor growth. 60% of tumors from Neo‐CRCVAS‐treated mice exhibited resistance to the treatment or continued to progress following the initial response. We hypothesized that the immunosuppressive TME of MSS‐CRLM may have limited the efficacy of vaccine treatment alone due to multiple immune evasion mechanisms. Thus, we analyzed the immunological changes in TME of Neo‐CRCVAS‐treated mice at day 15. Immunofluorescence (IF) and Immunohistochemistry (IHC) results revealed that, in Neo‐CRCVAS‐treated mice, lymphocytes were recruited to the tumor site, but the infiltration of lymphocytes into the tumor tissue and the secretion of IFN‐γ remained limited (Figure 2D). Additionally, TILs in Neo‐CRCVAS‐treated mice exhibited high expression of PD‐1 but low expression of activation marker 4‐1BB (Figure 2E); correspondingly, the obvious upregulation of PD‐L1 and downregulation of MHCI were confirmed by flow cytometry analysis in tumor tissues of Neo‐CRCVAS‐treated mice (Figure 2F,G). Moreover, Functional enrichment analysis based GOALL indicated a significant down‐regulation of antigen presentation, MHC expression, and cytotoxic cytokine genes in tumor tissue of Neo‐CRCVAS‐treated mice compared to those treated with PBS (Figure 2H). Such characteristics of TME in MSS‐CRLM worked together to promote immune escape following Neo‐CRCVAS monotherapy, which could be potentially reversed by regorafenib.

### 2.3 Neo‐CRCVAS Combined with Regorafenib Boosts Antitumor Immunity Response

Due to the limited infiltration of lymphocytes, upregulation of PD‐L1, and downregulation of MHCI after Neo‐CRCVAS treatment, it is reasonable to combine Neo‐CRCVAS with regorafenib, the only TKI known to enhance immune cell infiltration and reverse IFN‐γ‐induced MHC downregulation and PD‐L1 upregulation, to strengthen the antitumor immune response. To reassess the immunomodulatory effects of regorafenib on MSS‐CRLM, patient‐derived organoids (PDOs) of MSS‐CRLM were developed and subsequently treated with IFN‐γ and regorafenib. The findings indicated that, without impacting the IFN‐γ‐induced apoptosis of tumor cells (Figure S2A, Supporting Information), there was a significant rise in MHCI expression was observed, along with a notable reduction in PD‐L1 expression in PDOs treated with regorafenib (Figure S2B,C, Supporting Information).

**Figure 3:**
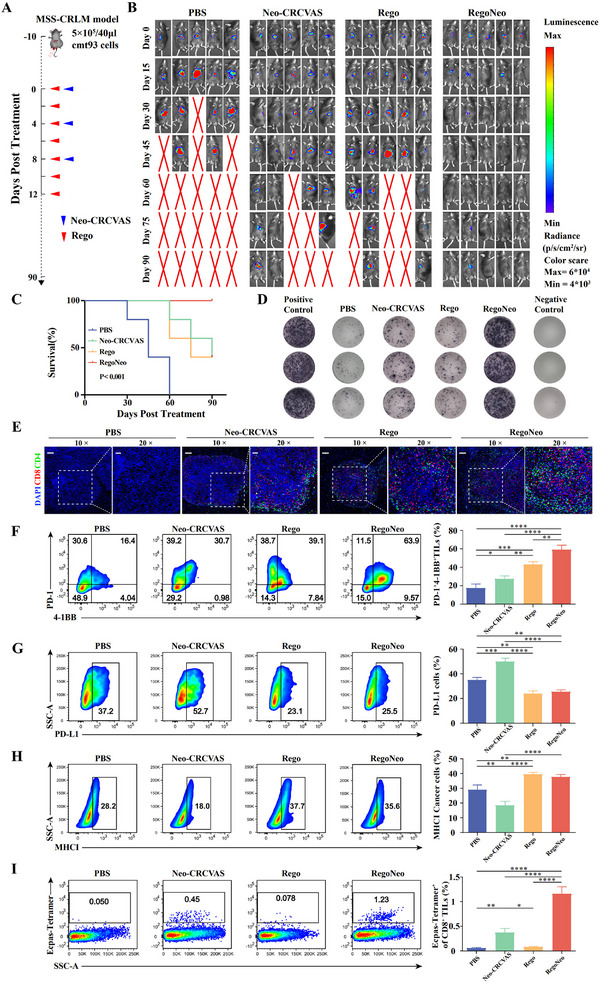
Antitumor efficacy of Neo‐CRCVAS plus regorafenib treatment (RegoNeo) in MSS‐CRLM mice model. A) Treatment timeline for Neo‐CRCVAS in MSS‐CRLM mice model. B) Tumor burden monitoring of PBS, Neo‐CRCVAS alone, Regorafenib alone and RegoNeo treated mice by bioluminescence imaging (*n* = 5). C) Kaplan‐Meier survival curves of PBS, Neo‐CRCVAS alone, Regorafenib alone and RegoNeo treated groups (*n* = 5). D) ELISPOT assay showing neoantigen specific‐reactivity of splenic T cells against seven neoantigen peptides in each treated group. E) The representative immunofluorescence image of CD4^+^ and CD8^+^ T cell infiltration in tumor tissues at each treated group, Scare bars, 100 µm (10×), 50 µm (20×). F) Flow cytometry showing the percentage of infiltrating CD8^+^ T cells expressing PD‐1 and 4‐1BB (*n* = 3). G) Flow cytometry showing the percentage of tumor cells expressing PD‐L1 (*n* = 3). H) Flow cytometry analysis showing the percentage of tumor cells expressing MHC1 (*n* = 3). I) Flow cytometry analysis showing the percentage of Ecpas_L1064F:H‐2K^b^ specific CD8^+^ T cells in infiltrating CD8^+^ T cells (*n* = 3). Ecpas, Ecpas_L1064F:H‐2K^b^. The statistical analysis was performed with ANOVA analysis. Survival curves were generated using Kaplan‐Meier estimates and tested using the log‐rank test. Results are shown as mean ± SD. *p <0.05; **p <0.01; ***p <0.001; ****p <0.0001.

Considering regorafenib's capacity to enhance immune cell infiltration, upregulate tumor cell MHC, and downregulate PD‐L1 expression, we subsequently evaluated the potential of Neo‐CRCVAS combined with regorafenib (RegoNeo) to elicit tumor‐specific immune responses. As depicted in **Figure** **3**A, orthotopic MSS‐CRLM mice were split into four groups and treated with PBS, Neo‐CRCVAS alone, regorafenib, and RegoNeo respectively. According to Figure 3B and Figure S3A, Supporting Information, tumor burdens of mice in the PBS group experienced a significant increase at day 15, ultimately resulting in the mortality of all mice at day 60 (5/5). Conversely, both the Neo‐CRCVAS and regorafenib groups exhibited a 20% incidence of consistently low tumor burdens and a 40% survival rate at day 90. Notably, mice in the RegoNeo group demonstrated the most significant tumor suppression, achieving 80% sustained tumor regression and a 100% survival rate compared to the other three groups. At the end of the study or following the death of the mice, tumors were extracted and their weight was measured. The tumor weight findings corroborated the bioluminescence images, further validating the antitumor efficacy of RegoNeo therapy (Figure S3B, Supporting Information). The Kaplan‐Meier analysis further revealed that mice treated with RegoNeo exhibited a significantly prolonged overall survival compared to those treated with PBS, Neo‐CRCVAS, or regorafenib alone (*p* < 0.0001, Figure 3C). Furthermore, to evaluate the safety of the RegoNeo treatment, biochemical tests were conducted, organ sections were stained with HE the 15th day after treatment (Figure S4A–N, Supporting Information), and body weight changes were monitored every three days (Figure S4O, Supporting Information). These findings provide evidence supporting the safety and viability of RegoNeo.

To further investigate if RegoNeo treatment can trigger immune responses against neoantigens, splenic T cells were collected on day 15 following RegoNeo therapy and tested for neoantigen‐specific reactivity against neoantigen pools using ELISPOT analysis. The findings indicated that mice undergoing combinational therapy exhibited a higher number of IFN‐γ spots compared to other treatment groups (Figure 3D; Figure S3C, Supporting Information). Subsequently, we evaluated the presence of TILs on the 15th day through IF staining following RegoNeo treatment. As expected, the results showed a notable increase in CD8^+^ and CD4^+^ T cell infiltration in the RegoNeo group compared to other groups (Figure 3E). Additionally, flow cytometry analysis showed that TILs from mice treated with RegoNeo exhibited increased expression of 4‐1BB and PD‐1 on CD8^+^ T cells, markers of T‐cell activation and proliferation, compared to those treated with PBS, Neo‐CRCVAS, or regorafenib alone (Figure 3F), suggesting that the combination therapy activates a broader TIL repertoire. Moreover, the tumor cells from mice treated with RegoNeo exhibited significantly decreased levels of PD‐L1 expression and increased levels of MHCI expression compared to other treatments, confirmed by flow cytometry analyses (Figure 3G,H).

Next, to evaluate the recruitment of tumor‐specific T cells to tumor tissues following RegoNeo therapy, we synthesized a fluorescently labeled tetramer targeting the highly immunogenic neoantigen peptide Ecpas_L1064F:H‐2K^b^ (Ecpas) in order to identify T cells expressing Ecpas‐specific T cell receptors (TCRs) within TILs. Figure 3I and Figure S3D, Supporting Information showed a significant rise in intratumoral Ecpas‐specific CD8+ T cells in mice treated with Neo‐CRCVAS, with or without regorafenib, compared to PBS or regorafenib alone, suggesting that RegoNeo therapy promotes the infiltration of neoantigen‐specific T cells into tumors. In summary, the findings suggested that the combination of Neo‐CRCVAS with regorafenib could effectively stimulate a robust neoantigen‐specific antitumor immune response in an in vivo model, indicating potential for MSS‐CRLM therapy.

### 2.4 RegoNeo Therapy in MSS‐CRLM Elicits Durable Immune Memory to Prevent Metastasis

**Figure 4:**
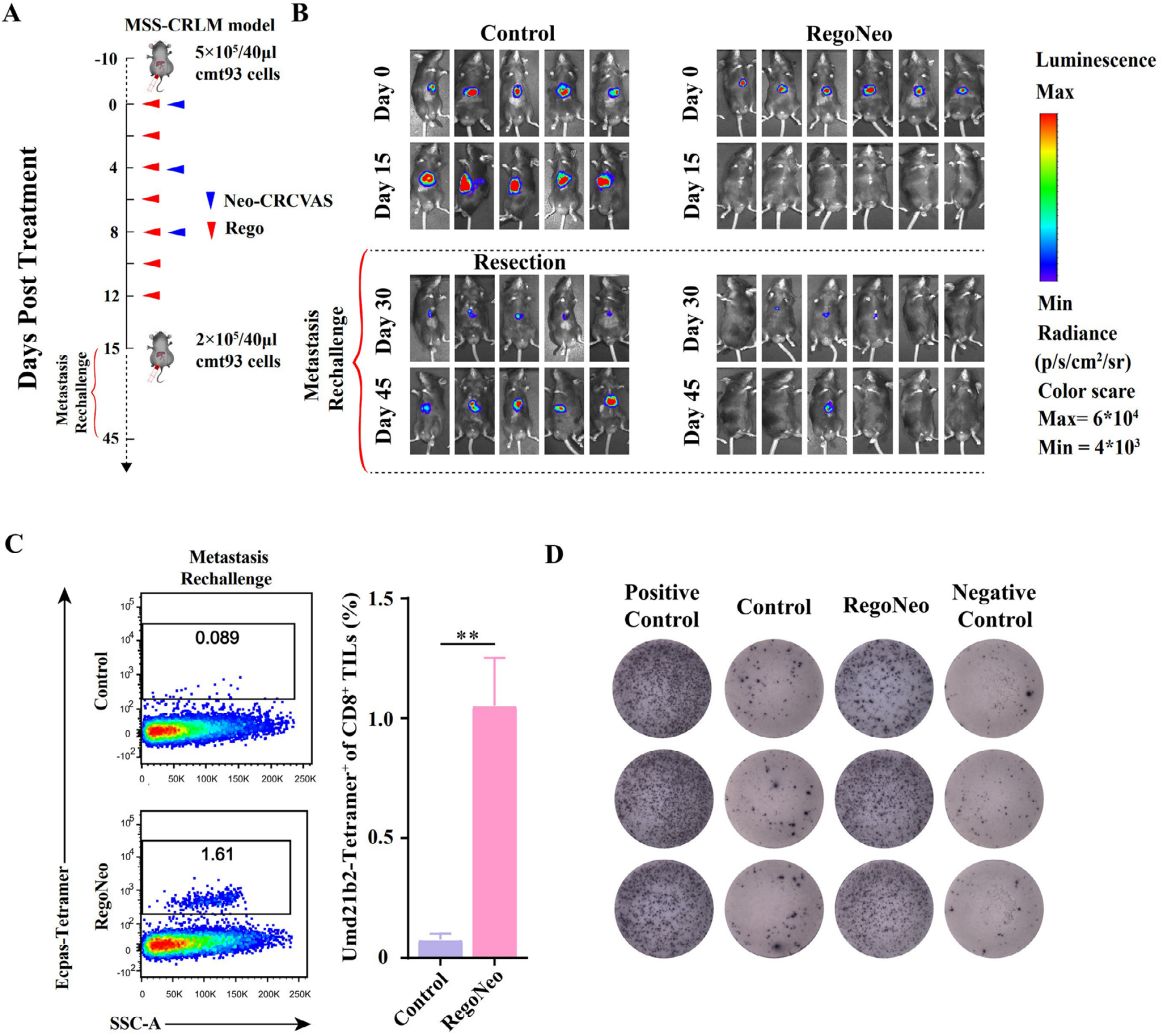
Metastasis rechallenge of RegoNeo treatment in vivo. A) Treatment timeline for the process of establishing metastasis rechallenge MSS‐CRLM models. B) Tumor burdens of RegoNeo treated mice (*n* = 6) and PBS treated mice (*n* = 5) after treatment and rechallenge measured by bioluminescence imaging. C) Flow cytometry analysis showing the percentage of Ecpas_L1064F:H‐2K^b^ specific CD8^+^ T cells in CRLM tissue after 2 days metastasis rechallenge (*n* = 3). D) ELISPOT assay showing neoantigen specific‐reactivity of splenic T cells against 7 neoantigen peptides at the day 50 after treatment (*n* = 3). Ecpas, Ecpas_L1064F:H‐2Kb. The statistical analysis was performed with ANOVA analysis. Results are shown as mean ± SD. *p < 0.05; **p <0.01; ***p <0.001; ****p <0.0001.

Considering the robust immune responses and therapeutic effects in MSS‐CRLM treatment achieved by RegoNeo therapy, we continued to explore its effectiveness in preventing metastasis. MSS‐CRLM mice were constructed and treated with either a combinational therapy or PBS, following established methods. Tumor burden was assessed via bioluminescence imaging, as shown in **Figure** **4**A. Fifteen days after treatment, all mice (*n* = 6) receiving RegoNeo showed tumor regression, whereas those treated with PBS (*n* = 5) experienced continued rapid tumor progression (Figure 4B). This result provided additional evidence for the effectiveness of RegoNeo therapy in treating MSS‐CRLM. Subsequently, mice that had been successfully treated with combination therapy were subjected to orthotopic injection of 2×10^5^ cmt93 cells from SMV on day 20 post‐treatment to simulate the metastasis of CRLM while tumor‐burden mice treated with PBS underwent surgical resection of the tumor and received the same cell injection serving as control. It is noteworthy that the tumor could not exhibit growth in 5 out of 6 mice cured by RegoNeo, whereas all control mice experienced a rapid increase in tumor burden (Figure 4B).

To evaluate the immune infiltration following metastasis rechallenge, the presence of TILs was assessed through IF staining of tumor tissue on the 45th day. As expected, the findings demonstrated a significant increase in CD8^+^ and CD4^+^ T cell infiltration in the RegoNeo group compared to the control group. (Figure S5A, Supporting Information). Moreover, Peptide‐MHC tetramer staining indicated a notable presence of Ecpas‐specific CD8^+^ T cells in tumor tissues from mice treated with RegoNeo therapy after metastasis rechallenge, while control mice exhibited minimal levels (Figure 4C; Figure S5B, Supporting Information). Significantly, ELISPOT analysis demonstrated that splenic T cells from mice receiving RegoNeo therapy retained significant sensitivity to neoantigen pools 45 days post‐treatment (Figure 4D; Figure S5C, Supporting Information). Collectively, these results indicated that the combination therapy, RegoNeo, for MSS‐CRLM, may elicit durable neoantigen‐specific immune memory responses, thereby potentially preventing metastasis after treatment.

### 2.5 Immune Status Changes in the TME Post‐Combination Therapy

**Figure 5:**
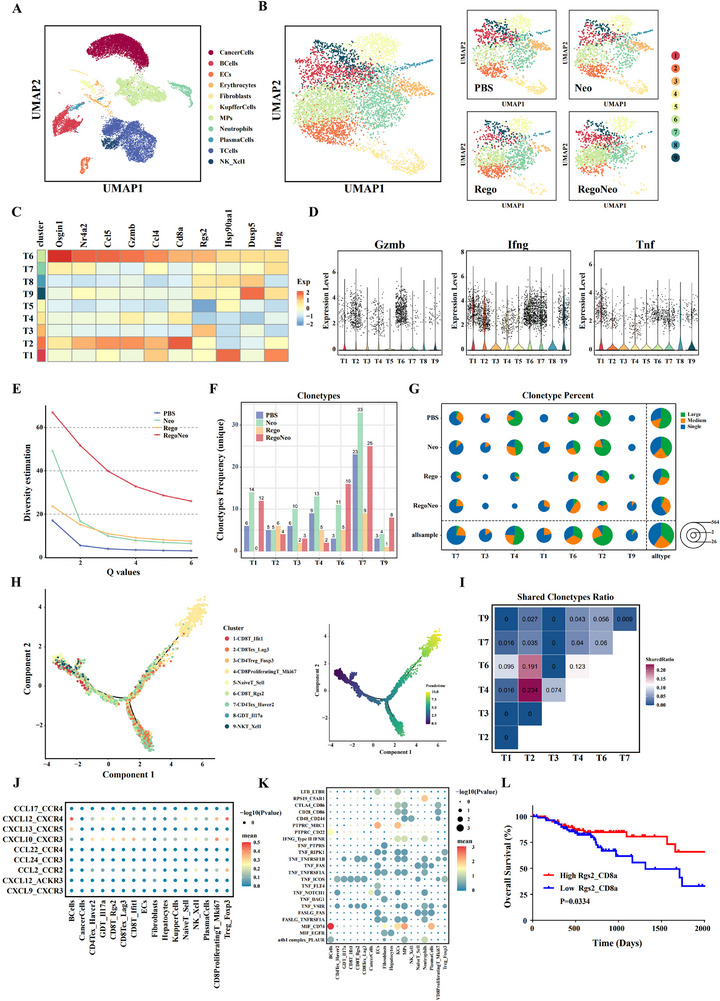
Characterization of immune microenvironment after RegoNeo treatment via scRNA‐seq data. A) UMAP plot showing cells merged from all groups. B) UMAP plot showing subsets of infiltrating T cell clusters merged from all groups (right) and each group (left). C) Heatmap showing the expression of top upregulated genes of T6 cluster. D) Expression levels of Gzmb, Ifng, Tnf across all T cell clusters. E) TCR clonetype diversity estimation of each treatment group using hill numbers. F) Unique TCR clonetype frequency across all T cell clusters. G) Clonetype percent across all T cell clusters of each treatment group, single, clonetype number is 1; median, clonetype number range from 2 to 10; large, clonetype number more than 10. H) Pseudotime trajectory of T cell clusters colored by clusters generated by Monocle2 (left); Pseudotime trajectory ofT cell clusters colored by pseudotime (dark blue to light blue) generated by Monocle2 (right). I) The overlap of TCR clonotypes between different T cell clusters. The number in each square indicating the overlap of clonotypes scaled to the length of unique clonotypes in the smaller sample. J) Bubble chart showing the cellular interactions involving chemokines between Rgs2^+^CD8^+^ T cell subset and other cell subsets. The sizes of the bubbles indicate the significance of the interactions between different subsets and the color indicated the communication probability calculated by Cellchat. K) Bubble chart showing the cellular ligand‐receptor interactions involving chemokines between Rgs2^+^CD8^+^ T cell subset and other cell subsets. The sizes of the bubbles indicate the significance of the interactions between different subsets and the color indicated the communication probability calculated by Cellchat. L) Kaplan‐Meier curves of 5‐year overall survival for patients with late‐stage CRC from TCGA stratified by median expression level of two‐gene signature (Rgs2 and CD8a). UMAP, uniform manifold approximation and projection. Survival curves were generated using Kaplan‐Meier estimates and tested using the log‐rank test.

To better understand immune status changes in the TME of MSS‐CRLM models after RegoNeo therapy, single‐cell RNA‐seq, and single‐cell V(D)J sequencing were conducted on single cells from tumor tissues treated with PBS Neo‐CRCVAS, regorafenib, and RegoNeo. After quality control, 12417, 9828, 14089, and 100341 cells were kept for each of the four treated groups. For each group, 7726 cells were randomly chosen for further analysis. The Uniform Manifold Approximation and Projection (UMAP) identified 11 cell clusters (**Figure** **5**A). Of these, the B cell cluster, endothelial cell cluster, kupffer cell cluster, NK cell cluster, and T cell cluster were found to be enriched in cells derived from RegoNeo‐treated mice compared to those from PBS‐treated mice (Figure 5A; Figure S5A, Supporting Information). Given the pivotal role of T cells in the antitumor responses, our study subsequently concentrated on the T cell clusters.

In order to more thoroughly analyze the diversity present within the T cell population, the T cell clusters were isolated and subjected to further clustering, yielding 9 distinct T cell clusters (Figure 5B). It was observed that a significant reduction in Tregs_Foxp3 and CD8^+^ Tex_ Lag3 within the RegoNeo group. Notably, the T6 cluster represented a subpopulation of CD8^+^ T cells predominantly found in the RegoNeo‐treated group, with minimal presence in the remaining three groups (Figure 5B; Figure S5B, Supporting Information). The identification of marker genes in the T6 cluster revealed enrichment of Cd8a, Rgs2, Gzmb, Ccl4, Ccl5, and Ifng genes. Based on this, we subsequently referred T6 cluster as Rgs2^+^CD8^+^T cell (Figure 5C). In addition to Gzmb and Ifng, the T6 cluster also exhibited a relatively high expression of Tnf, thereby corroborating the significant contributions of Rgs2^+^CD8^+^ T cells in the immune response against cancer (Figure 5D).

To further explore the variability of TCR clonotypes induced by combination treatment, we initially conducted a comparison of TCR clonotype diversities among different groups. Notably, the group treated with RegoNeo demonstrated the greatest number of TCR clonotypes (Figure 5E; Figure S5C, Supporting Information), especially within the Rgs2^+^CD8^+^ T cell subset, which also exhibited a high frequency of unique clonotypes in comparison to the other groups (Figure 5F; Figure S5D, Supporting Information). Moreover, a majority of TCR clonotypes in Rgs2^+^CD8^+^ T cells from the combination therapy group had a median to large expansion, as indicated by clonotype percent analysis (Figure 5G). Altogether, these population dynamics observed suggested that the emergence of new TCR clonotypes and a substantial expansion of TCR clonotypes following RegoNeo therapy led to a shift in the TCR repertoire, which was potentially associated with efficacy immunotherapy efficacy.

Furthermore, the Monocle 2 algorithm was employed for pseudotime analysis, identifying two primary evolutionary pathways of T cells: one culminating in exhausted T cells (T2_CD8_Lag3_Tex and T7_CD8_Havcr2) and the other leading to T4_CD8_Mki67_Proliferating T (Figure 5H). The trajectory initiated with T5_Naive T cells, succeeded by T1_CD8_Ifit1_T cells. Subsequently, T1_CD8_Ifit1_T cells differentiated into Rgs2^+^CD8^+^ T cells, ultimately resulting in exhausted T cells (T2_CD8_Lag3_Tex and T7_CD8_Havcr2_Tex). Moreover, TCR sharing analysis provided additional evidence that Rgs2^+^CD8^+^ T cells exhibited a high degree of TCR similarity with T1_CD8_Ifit1_T cells and T2_CD8_Lag3_Tex, suggesting a potential evolutionary relationship among the three cell types (Figure 5I; Figure S5D, Supporting Information). Additionally, analysis of cellular interactions involving chemokines revealed that Rgs2^+^CD8^+^ T cells and B cells communicated most frequently through CXCL12/CCR4 (Figure 5J). The further analysis of ligand‐receptor interactions indicated that Rgs2^+^CD8^+^ T cells most frequently communicated through the MIF/CD74 interaction (Figure 5K). Moreover, the Results also showed a relatively higher expression of MIF in Rgs2^+^CD8^+^T cells and CD74 in B cells in RegoNeo groups, thereby corroborating that finding (Figure S6E, Supporting Information). Interestingly, the RegoNeo‐treated group showed a notable enrichment of both Rgs2^+^CD8^+^T cells and B cells, suggesting that the recruitment of B cells by Rgs2^+^CD8^+^T cells via CXCL12/CCR4, followed by interaction through the MIF/CD74 pathway, could potentially enhance the antitumor response. Kaplan‐Meier analysis of the TCGA dataset further validated the prognostic significance of the RGS2_CD8a signature in late‐stage CRC, showing that tumors with high RGS2_CD8a expression had notably better 5‐year OS rates (*p* = 0.0334, Figure 5L).

### 2.6 Validation of the Antitumor Potential of Rgs2^+^CD8^+^ T Cells in MSS‐CRLM Treatment

**Figure 6:**
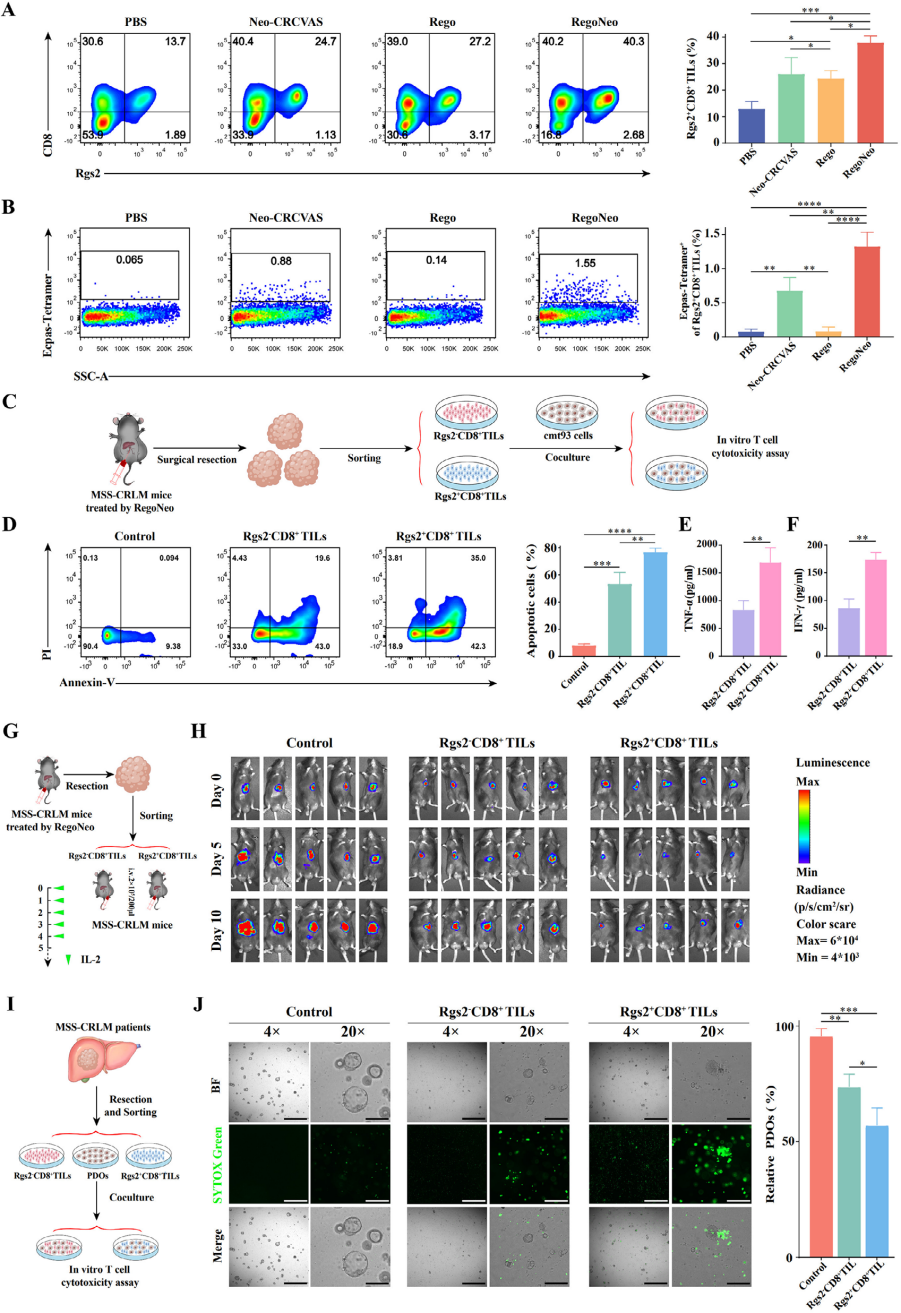
Infiltration and anti‐tumor efficacy of Rgs2^+^CD8^+^ T cells. A) Flow cytometry analysis showing the percentage of Rgs2^+^CD8^+^ TILs in infiltrating CD8^+^ T cells of PBS, Neo‐CRCVAS alone, Regorafenib alone and RegoNeo treated groups (*n* = 3). B) Flow cytometry analysis showing the percentage of Ecpas_L1064F:H‐2K^b^ specific CD8^+^ T cells in infiltrating Rgs2^+^CD8^+^ TILs (*n* = 3). C) Schematic diagram of in vitro tumor‐killing efficacy evaluation of Rgs2^+^CD8^+^ and Rgs2^−^CD8^+^ TILs cells isolated from MSS‐CLRM tumor tissues after combined treatment. D) Flow cytometry showing the percentage of apoptotic cells induced by Rgs2^+^CD8^+^ or Rgs2^−^CD8^+^ TILs (*n* = 3). E) ELISA assay showing secretion of TNF‐α from Rgs2^+^CD8^+^ or Rgs2^−^CD8^+^ TILs (*n* = 3). F) ELISA assay showing secretion of IFN‐γ from Rgs2^+^CD8^+^ or Rgs2^−^CD8^+^ TILs (*n* = 3). G) Schematic diagram of adoptive Rgs2^+^CD8^+^ or Rgs2^−^CD8^+^ TILs therapy in MSS‐CRLM mouse models. H) Tumor burden monitoring of mice after adoptive Rgs2^+^CD8^+^ or Rgs2^−^CD8^+^ TILs therapy by bioluminescence imaging (*n* = 5). I) Schematic diagram of in vitro tumor‐killing efficacy evaluation of Rgs2^+^CD8^+^ or Rgs2^−^CD8^+^ T cells isolated from MSS‐CRLM patient's tumor tissue. J) PDOs death induced by Rgs2^+^CD8^+^ or Rgs2^−^CD8^+^ TILs as determined by SYTOX Green assay ((left)), histograms showing the percentage of relative PDOs in each group (right). The statistical analysis was performed with ANOVA analysis. Results are shown as mean ± SD. *p < 0.05; **p < 0.01; ***p < 0.001; ****p < 0.0001.

Encouraged by the above findings, it was hypothesized that there would be a substantial presence of neoantigen‐specific TILs in Rgs2^+^CD8^+^ T cells following the combinational therapy. Flow cytometry analysis and IF staining of TILs from the treated groups demonstrated an enrichment of Rgs2^+^CD8^+^ T cells in RegoNeo‐treated groups (**Figure** **6**A; Figure S7A, Supporting Information). Moreover, the flow cytometry analysis revealed a significantly higher percentage of Ecpas‐specific CD8^+^ T cells within Rgs2^+^CD8^+^ T cells after combinational therapy compared to the other three groups (Figure 6B; Figure S7B, Supporting Information), thus confirming this hypothesis. These findings provided further evidence of the enhanced infiltration of neoantigen‐specific Rgs2^+^CD8^+^ T cells following the RegoNeo therapy.

Furthermore, Rgs2^+^CD8^+^ T cells and Rgs2^−^CD8^+^ T cells were separated from mice that underwent RegoNeo therapy, using flow cytometry sorting and assessed for tumor‐killing and cytokine secretion ability. Subsequently, these TILs were cocultured with cmt93 cells for 48 h (Figure 6C). Intriguingly, the flow cytometry demonstrated an elevated level of apoptotic cells (Figure 6D) and an increase in IFN‐γ/TNF secretion in the Rgs2^+^CD8^+^ T cells cocultured group compared to the Rgs2^−^CD8^+^ T cells, consistent with the results from ELISA assays. (Figure 6E,F).

Additionally, we assessed the efficacy of Rgs2^+^CD8^+^ T cells in MSS‐CRLM mice model. As demonstrated in Figure 6G, Rgs2^+^CD8^+^ T cells and Rgs2^−^CD8^+^ T cells from RegoNeo‐treated tumors were intravenously injected into MSS‐CRLM mice (*n* = 5), with subsequent IL‐2 administration for five days. The control group consisted of mice treated solely with IL‐2 (*n* = 5). Mice injected with Rgs2^+^CD8^+^ T cells showed a significantly lower tumor burden than those in the control and Rgs2^−^CD8^+^ T cell groups (Figure 6H; Figure S7C, Supporting Information), suggesting the crucial role of Rgs2^+^CD8^+^ T cells in antitumor immunity.

To further validate these findings in MSS‐CRLM patients, Rgs2^+^CD8^+^ T cells and Rgs2^−^CD8^+^ T cells were isolated from freshly excised CRLM tumor tissues and cocultured with autologous PDOs for 48 h (Figure 6I). Of note, SYTOX Green Assay showed significantly more apoptosis of PDOs in Rgs2^+^CD8^+^ T cells cocultured groups (Figure 6J), providing compelling evidence for the critical role of Rgs2^+^CD8^+^ T cells in anti‐tumor immunity.

## 3 Discussion

MSS‐CRLM remains one of the most challenging malignancies for immunotherapy, primarily due to its immunosuppressive TME, characterized by poor immune cell infiltration, low TMB, and resistance to immune checkpoint blockade.^[^ ^7^, ^28^ ^]^ The limited effectiveness of current immunotherapies is attributed to the absence of tumor‐specific TILs, elevated levels of immunosuppressive molecules like PD‐L1, and diminished MHC molecule expression on tumor cells.^[^ ^29^, ^30^ ^]^ Therefore, there is a critical demand for the development of novel strategies that can reprogram its immunosuppressive TME and induce tumor‐specific TILs, enabling a robust and durable antitumor immune response. Neoantigen vaccines have shown clinical benefits by inducing antigen‐specific T‐cell immune responses in serval solid tumors.^[^ ^18^ ^]^ Further research is still needed to explore its effectiveness in MSS‐CRLM.^[^ ^31^ ^]^ In our study, we initially developed a personalized neoantigen vaccine, Neo‐CRCVAS. Following subcutaneous administration of the peptides, antigen‐presenting cells consistently internalized, processed and presented these peptides on MHCI molecules. This approach demonstrated promising therapeutic effects in the MSS‐CRLM mouse model. Nevertheless, we also observed that, in addition to the lack of immune cell infiltration, tumor cells treated with CRC‐NeoVAS alone exhibited a high level of PD‐L1 and a low level of MHC, which was prone to T cell exhaustion and deficiency of tumor antigen presentation function. Remarkably, the combination of CRC‐NeoVAS and regorafenib (RegoNeo) significantly enhanced the neoantigen‐specific immune response by reprogramming TME. This combined therapy has demonstrated excellent synergistic anti‐tumor effects and durable tumor‐specific immunological memory in the preclinical MSS‐CRLM model and metastasis rechallenge. These findings indicate the potential effectiveness of this treatment strategy in clinical practice for MSS‐CRLM.

While CD8^+^ T cells constitute the predominant subset of TILs and play a crucial role in immune responses in solid tumors, only a few subsets have strong anti‐tumor capabilities through targeting tumor antigens, regulating immune cell chemotaxis, and secreting cytotoxic factors.^[^ ^32^, ^33^ ^]^ Our previous study has demonstrated that CD8^+^T_RM_ cells with neoantigen‐specific TCR may be a promising immunotherapeutic target for HCC.^[^ ^27^ ^]^ However, the key subset of MSS‐CRLM still needs to be deeply explored. In this study, we identified a notable enrichment of Rgs2^+^CD8^+^ T cells derived from T1_CD8_Ifit1_T cells in MSS‐CRLM tumor tissue following RegoNeo treatment compared to other treated groups. Particularly noteworthy was the increase in both the diversity and expansion of TCR clonotypes within this subset. Furthermore, our study confirmed that this subset exhibited a higher proportion of neoantigen‐specific T cells and an increased secretion of tumoricidal cytokines compared to other T cell subsets. In both in vitro and in vivo MSS‐CRLM mouse models or PDO models, Rgs2^+^CD8^+^ T cells demonstrated significant tumor‐killing capabilities. Overall, our findings provide compelling evidence for the critical role of Rgs2^+^CD8^+^ T cells in the immune response against MSS‐CRLM.

In addition, G protein signal transduction regulator 2 (Rgs2) serves as a regulator of G protein‐coupled receptors, which could influence the targeted migration of immune cells toward the tumor site and fully activate, as well as secretion of cytokines, thereby enhancing the synergistic tumor‐killing ability.^[^ ^34^ ^]^ Research indicates that Rgs2 is integral to the pathophysiology of various tumors, with its expression levels in colorectal cancer being strongly correlated with patients’ prognosis. Notably, the downregulation of Rgs2 is significantly associated with the recurrence and metastasis of CRC, and diminished expression of Rgs2 serves as a prognostic marker for reduced survival rates.^[^ ^35^ ^]^ However, the extent to which Rgs2^+^CD8^+^ T cells possess these capabilities in MSS‐CRLM remains further investigated. Here, we found that Rgs2^+^CD8^+^ T cells had the potential of cytokine secretion, such as IFN‐γ and TNF, to activate other immune cells involved in the anti‐tumor response. Additionally, Rgs2^+^CD8^+^ T cells also exhibit high expression of various chemokines including CCL4 and CXCL12, which may facilitate the recruitment of B cells to tumor sites and subsequently engage in interactions through the MIF/CD74 pathway to enhance activation and proliferation, thereby augmenting the anti‐tumor immune response to augment the anti‐tumor immune response. Notably, scRNA sequencing data also revealed a significant enrichment of B cells in the RegoNeo group. These findings suggest that Rgs2^+^CD8^+^ T cells may collaborate with other immune cells such as B cells to boost anti‐tumor immunity.

In conclusion, our study showed that combining CRC‐NeoVAS with regorafenib, termed RegoNeo, effectively induced a robust antitumor response and established durable tumor‐specific immune memory in the MSS‐CRLM model by reprogramming the immunosuppressive TME. Furthermore, we observed a significant enrichment of the Rgs2^+^CD8^+^ T cell subset in tumor tissues following the combined treatment, which consisted of a large proportion of neoantigen‐specific T cells and was closely associated with the therapeutic efficacy. Overall, these results suggest a potentially promising strategy to improve the antitumor immune response through increasing the number and activation of Rgs2^+^CD8^+^ T cells in patients with MSS‐CRLM, although further validation is still required in other preclinical models and clinical trials. However, due to the limited MSS models used in this study, the corresponding findings still need to be further validated in other MSS animal models and clinical trials.

## 4 Experimental Section

### Identification and Validation of Neoantigen Immunogenicity

WES and RNA‐sq were conducted on DNA and RNA from Murine CRC cell line cmt93 and C57BL/6 mouse tail tissue. Mutation variants in cmt93 cells were identified using VarScan^[^ ^36^ ^]^ software, utilizing the mouse genome mm10 as a reference. Only mutations with a variant allele frequency (VAF) greater than 10% were included in the analysis. The remaining variants underwent annotation using wANNOVAR^[^ ^37^ ^]^ in order to identify and filter out nonsynonymous mutations.

The immunogenicity of all mutations was assessed by utilizing the NetMHCpan^[^ ^38^ ^]^ binding affinity predictor, with mutations generating 9‐mer mutant peptides exhibiting an IC50 < 500 nM to the H‐2Kb allele being identified as potential neoantigens. Detailed methodology is available in the Supporting Materials and Methods section.

### Validation of Neoantigen Immunogenicity

Male C57BL/6 mice (aged 6–8 weeks) were sourced from China Wushi, Inc. in Shanghai, China, with approval from the Animal Ethics Committee of Fujian Medical University (IACUC FJMU 2023‐Y‐0500). To identify potential neoantigen peptides, 22 mutations from cmt93 cells were selected, with 20 synthesized into long peptides by Genscipt Biotechnology Co., Ltd, China.

To assess neoantigen immunogenicity, 20 neoantigen peptides were split into two pools (10 µg each) and mixed with 50 µg Poly(I:C) (Guangdong South China Pharmaceutical Co., Ltd). The mixture was subcutaneously administered to male C57BL/6 mice at the lateral flank on days 0, 4, and 8. On day 14, the mice were euthanized and splenic T cells were quantified for ELISPOT assay. Additional methodological details are available in the Supporting Materials and Methods section.

### Antitumor Efficacy in Vivo Evaluation

The MSS‐CRLM model was established by intrasplenic injection of 5×10^5^ cmt93‐luc cells into the SMV of mice over a period of 10 days. To assess the antitumor efficacy of the neoantigen vaccine both independently and in combination with other treatments in vivo, the MSS‐CRLM model was categorized into four groups, each containing five subjects. Mice in each group received subcutaneous injections of identified neoantigen peptides (10 µg/peptide) combined with 50 µg Poly(I:C) in a 200 µL volume on days 0, 4, and 8. Regorafenib (Bayer, 73–4506) was given orally every two days starting from day 0, for a total of 7 doses at 10 mg kg^−1^. The formulation included 34% PEG400 (Sigma‐Aldrich), 12% pluronic F68 (Thermo Fisher Scientific), and 20% water, following the manufacturer's guidelines. Mice treated with PBS served as the control group. Tumor burden in the MSS‐CRLM model was assessed at 15‐day intervals using the IVIS Spectrum animal imaging system (PerkinElmer, USA). All mice were re‐injected with 2×10^5^ cmt93 cells via the SMV again 50 days after a successful cure with combination therapy for metastasis rechallenge experiments. Mice with tumor burden treated with PBS underwent surgical tumor resection and served as controls. The tumor burden was subsequently monitored every 15 days. Detailed descriptions of the methods used for assessing therapeutic efficacy, tissue processing, flow cytometry, tetramer staining, sequencing, IF, IHC, and HE staining are provided in the Supporting Materials and Methods section.

### Analysis of Single‐Cell RNA Sequencing (scRNA‐seq) and Bulk RNA‐Sequencing (RNA‐seq) Data

Single‐cell suspensions were collected from tumor tissue in the MSS‐CRLM model following treatment with Neoantigen, regorafenib, or both. The suspension was prepared for scRNA‐seq using the 10×Genomics Chromium Single Cell 5′ Library & Gel Bead Kit and the V(D)J Enrichment Kit. Cell Ranger was used to align scRNA‐seq reads from the 10×Genomics platform to the mm10 reference genome. Seurat^[^ ^39^ ^]^ in R (v4.1.0) was used to filter qualified UMI reads and cells.

After excluding low‐quality cells and probable doublets, 7726 cells were randomly selected from each group for analysis. Gene expression levels underwent normalization via the LogNormalize method. Seurat functions were utilized for t‐SNE dimensionality reduction and cell clustering. Cell clusters were annotated using the SingleR package (v1.6.1),^[^ ^40^ ^]^ utilizing data from the Immgen^[^ ^41^ ^]^ and MouseRNAseq datasets. The Supporting Materials and Methods section provided an in‐depth account of the methodologies used, including single‐cell V(D)J analysis, T‐cell development trajectory analysis, cell‐cell interaction analysis, and Bulk RNA‐seq data analysis.

### In Vitro and In Vivo T Cell Antitumor Efficacy Assay

Rgs2^+^CD8^−^/ Rgs2^+^CD8^+^ T cells were isolated from fresh tumor tissues from MSS‐CRLM mice models and patients. Methods for T cell antitumor efficacy assay in vitro were detailed in the Supporting Materials and Methods section. For the in vivo T cell antitumor efficacy assay, either Rgs2^+^CD8^−^/ Rgs2^+^CD8^+^ T cells (2×10^5^ per mouse) were administered via intravenous injection into the MSS‐CRLM model, with each group consisting of five mice (*n* = 5). MSS‐CRLM mice treated with PBS served as the control group. Subsequently, all treated mice were administered a daily injection of 1×10^3^ IU IL‐2 for 5 consecutive days. The tumor burden was monitored at intervals every 5 days as previously described.

### MSS‐CRLM Patient‐Derived Organoids (PDOs) Generation

Fresh tumor tissue samples were rinsed with Cancer Organoid Basal Medium (#B213152, bioGenous) until the supernatant was clear, then minced into small fragments, digested with Tumor Tissue Digestion Solution (#K601003, bioGenous) in SmartOrgan Dissociator (bioGenous) at 37 °C for 5–10 min. The digestion was terminated by adding 2%–4% fetal bovine serum (FBS; #B118‐500, Nobimpex). The supernatant was collected, passed through a 70 µm filter (#CSS013070, BIOFIL), and centrifuged. The erythrocytes were lysed with Red Blood Cell Lysis Solution (#E238010, bioGenous) and then centrifuged. The cell pellet was suspended in Organoid Culture ECM (#M315066, bioGenous) and plated into 24 well plates (#H803002, BDBIO). The organoids were overlaid with the Human Colorectal Cancer Organoid Medium (#K2103‐CR, bioGenous). The medium was changed every 2 days. The organoids can be passaged by Organoid Dissociation Solution (#E238001, bioGenous) or cryopreserved by Organoid Cryopreservation Medium (#E238023, bioGenous).

### In Vitro T Cell Cytotoxicity Assay on PDOs

In order to evaluate the cytotoxic effects of Rgs2^+^CD8^+^ T cells on PDOs, Rgs2^−^CD8^+^ T /Rgs2^+^CD8^+^ T cells were isolated from fresh CRLM tumor samples obtained from patients. Low‐adherence 96‐well plates(#30096L, BDBIO) were coated with 20% ECM (30 µL/well, 37 °C, 30 min). Organoids were harvested using an ice‐cold Cancer Organoid Basal Medium. Subsequently, Rgs2^−^CD8^+^ T /Rgs2^+^CD8^+^ T cells (5 × 10^4^ cells per well) and PDOs (1 × 10^4^ cells per well) were co‐cultured for 48 h in a 96‐well plate with IL‐2 (10 ng mL^−1^, R&D systems, MX2918061) and OrganoidpleX Medium (150 µL, #CO1233, bioGenous) at 37 °C in a 5% CO2 environment. Bright‐field imaging was established on day 1 and day 3. On Day 3, SYTOX Green (1:1000; #S7020, invitrogen) was added (50 µL/well, 30 min dark incubation) for fluorescence and bright field images and viability assessment. Finally, photos were taken under a microscope at after fixed.

### Statistical Analysis

The correlation matrices were assessed using Pearson's correlation coefficient. Kaplan‐Meier estimates were utilized to construct survival curves, with significance determined by the log‐rank test. A two‐tailed Student's t‐test or a one‐way ANOVA was used for the statistical comparison of the sample data in GraphPad Prism 8.0, with statistical significance defined as *p < 0.05. **p < 0.01, ***p < 0.001, ****p < 0.001. Data were presented as means ± standard deviation (SD) based on a minimum of three independent biological samples.

### Ethics Approval and Consent to Participate

Written informed consent was obtained from MSS‐CRLM patients to obtain tumor tissues for analysis. All procedures were performed in accordance with the Declaration of Helsinki. This study was approved by the Ethics Review Committee of The First Affiliated Hospital of Fujian Medical University (YAN[2023]110) and the Animal Ethics Committee of Fujian Medical University (IACUC FJMU 2023‐Y‐0500)

### Consent for Publication

This manuscript has not been submitted elsewhere for publication in whole or in part, and all the listed authors have approved the manuscript that is enclosed.

## Conflict of Interest

The authors declare no conflict of interest.

## Author Contributions

H.C., G.G., and J.Z. performed conception and design. H.C., B.C., Y.Y., S.L., H.C., Z.Z., and W.Y. performed development of methodology. H.C., B.C., Y.G., Y.C., X.L., Z.Z., and S.L. performed Acquisition of Data. H.C., B.C., Y.Y., G.G., and J.Z. performed analysis and interpretation of data. H.C., B.C., Y.Y., G.G., and J.Z. performed wrote, reviewed and/or revision of the manuscript. G.G., J.Z. performed Study Supervision. H.K.C., B.C., and Y.F.Y. contributed equally to this work.

## Supporting information



Supporting Information

## Data Availability

The data that support the findings of this study are available from corresponding author upon reasonable request.
