# Supplementary material for: Personalized Neoantigen Vaccine plus Regorafenib Increases Rgs2⁺CD8⁺ T Cells Infiltration and Reprograms the Tumor Microenvironment in Microsatellite Stable Colorectal Cancer Liver Metastases
Source: Adv Sci (Weinh). 2025 Jun 30;12(36):e08040. doi: 10.1002/advs.202508040 (PMC12462951; doi:10.1002/advs.202508040)
Supplement: Supplementary file 1 — Supporting Information [file ADVS-12-e08040-s001.pdf]

ADVANCED SCIENCE
Open Access

Supporting Information

for *Adv. Sci.*, DOI 10.1002/advs.202508040

# Personalized Neoantigen Vaccine plus Regorafenib Increases Rgs2$^+$CD8$^+$ T Cells Infiltration and Reprograms the Tumor Microenvironment in Microsatellite Stable Colorectal Cancer Liver Metastases

*Hengkai Chen, Bin Chen, Yuanfeng Yang, Shoufeng Li, Huajun Cai, Zhicheng Zhuang, Yong Wu, Yuan Gao, Yupeng Chen, Xing Liu, Guoxian Guan* and Jinfu Zhuang**

# Personalized Neoantigen Vaccine plus Regorafenib Increases Rgs2$^+$CD8$^+$ T Cells Infiltration and Reprograms the Tumor Microenvironment in Microsatellite Stable Colorectal Cancer Liver Metastases

Hengkai Chen [1,2]†, Bin Chen [1,2]†, Yuanfeng Yang [1,2]†, Shoufeng Li [1,2], Huajun Cai [1,2], Zhicheng Zhuang [1,2], Yong Wu [1,2], Yuan Gao [1,2], Yupeng Chen[1,2], Xing Liu [1,2], Guoxian Guan [1,2]*, Jinfu Zhuang [1,2]*

[1] Department of Colorectal Surgery, the First Affiliated Hospital of Fujian Medical University, Fuzhou 350005, China.

[2] Department of Colorectal Surgery, National Regional Medical Center, Binhai Campus of the First Affiliated Hospital, Fujian Medical University, Fuzhou 350212, China.

[3] Fujian Abdominal Surgery Research Institute, the First Affiliated Hospital, Fujian Medical University.

## Experimental Methods Section

### Cell line

Murine CRC cell line cmt93 cell was obtained from the American Type Culture Collection (ATCC). To generate Cmt93 cells expressing luciferase (cmt93-luc), the cells were transfected with a lentivirus containing the luciferase reporter gene, sourced from Shanghai Genechem Co., Ltd, for a duration for 48 hours. Following lentiviral transduction, stable expression of the luciferase gene was achieved by selecting cells with 2 μg/mL puromycin. Both CMT93 and CMT93-luc cells were cultured in

Dulbecco's Modified Eagle Medium (DMEM) supplemented with 10% fetal bovine serum (FBS), under conditions of 37°C and 5% CO2.

**Neoantigen identification and immunogenicity validation**

The DNA and RNA from cmt93 cells and C57BL/6 mouse tail tissues followed by whole-exome sequencing and transcriptomic sequencing utilizing the Genomic DNA kit (Tiangen Biotech, China) and the EasyPure RNA kit (Transgen Biotech, China) in accordance with the manufacturer's protocols. Subsequently, the DNA samples underwent DNA library preparation and whole-exome capture using the SureSelect XT Mouse All Exon Kit, while the RNA samples were prepared for sequencing with the VAHTS Stranded mRNA-seq Library Prep Kit. The sequencing of both DNA and RNA libraries was carried out by Berry Genomics (Beijing) on Illumina Novoseq 6000 (paired end, 150bp).

Mutations in cmt93 cells were identified using VarScan software with the mouse genome mm10 serving as the reference. Only mutations with a variant allele frequency (VAF) >10%. Variants were annotated using wANNOVAR to filter for nonsynonymous mutations. Mutations with <20 variant allele reads were excluded, and those with VAF >60% were also discarded also to remove possible germline mutations.

All mutations were confirmed at the RNA level using the bam2R algorithm from the R package DeepSNV (v1.24.0). Mutations with a VAF of ≥ 10% in RNA-seq data, located within genes exhibiting sufficient expression levels (TPM>1), were retained for further analysis.

The immunogenicity potential of these mutations was assessed using the

NetMHCpan binding affinity predictor, with mutations generating 9-mer mutant peptides exhibiting an IC50 < 500nM to the $H2\text{-}K^b$ allele being identified as potential neoantigens. Subsequently, these candidates were evaluated in C57BL/6 mouse tail tissues using DeepSNV, with mutations demonstrating a sequencing depth exceeding 20× and no variant allele detected in all three mouse tail tissues being selected for further analysis in the subsequent investigation.

In order to identify potential neoantigen peptides, 20 neoantigen mutations chosen from cmt93 cells were synthesized into long peptides with high purity (>95%) from Genscipt Biotechnology Co., Ltd in China. For validation of neoantigen immunogenicity, these peptides were randomly divided into 2 pools (10μg/peptide) and combined with 50μg Poly(I:C) from Guangdong South China Pharmaceutical Co., Ltd for subcutaneous immunization of male C57BL/6 mice at the lateral flank on day 0, day 4, and day 8.

To find potential neoantigen peptides, 20 mutations were chosen from Hepa1-6 cells and synthesized into long peptides (17 amino acids) with high purity (>95%). These peptides were then divided into 2 pools and combined with Poly(I:C) for immunization of male C57BL/6 mice on days 0, 4, and 8. Erythrocytes were eliminated using 1 mL of Red Blood Cell Lysis Solution (Gibco), after which the resulting splenic T cells were subjected to two washes with PBS and subsequently enumerated for ELISPOT analysis.

**Enzyme-linked immunospot (ELISPOT) assay**

The secretion of IFN-γ by mouse splenic T cells was assessed using an ELISPOT

kit (Mabtech, 3321-4APT-10). Bone marrow-derived dendritic cells (BMDCs) were obtained through the following procedure: Femurs and tibias from 6-8 week-old naive C57BL/6 mice were isolated, with residual soft tissue and epiphyses removed. Marrow was collected by flushing the canals with PBS, followed by centrifugation at 800g for 5 minutes at room temperature. The precipitated cells were then lysed with 1mL of Red Blood Cell Lysis Solution (Gibco) for 4 minutes, centrifuged at 800g for 5 minutes, and washed twice with PBS. On day 0, 2 million cells were added to each well of a 6-well plate and cultured with 2 mL of RPMI-1640 medium containing 10 ng/mL IL-4 (404-ML-010/CF) and 20 ng/mL mGM-CSF (R&D systems, 415-ML-020/CF) to generate BMDCs at 37°C with 5% CO2. Half of the medium was replaced on day 3. On day 6, BMDCs were exposed to a neoantigen peptide pool (4μg total, 0.55μg per peptide) or individual peptides (4μg each) for 48 hours. For the ELISPOT assay, $3\times10^4$ BMDCs (previously exposed to the neoantigen peptide) were co-cultured with $3\times10^5$ splenic T cells in a multiscreen 96-well filtration plate (Mabtech, 3321-4APT-2) at 37°C with 5% CO2 for another 48h. The plates were washed and then incubated with detection antibody (R4-6A2-biotin,1 μg/mL, 100μl/well) for 2 hours. After another wash, they were incubated with Streptavidin ALP (1:1000 dilution, 100μl per well) for 1 hour. Then, 3, 3', 5, 5'-Tetramethylbenzidine (TMB) substrate solution was added and incubated before stopping the reaction with deionized water. IFN-γ spot-forming cells were imaged and analyzed using an ELISPOT Analysis System (AT-Spot-2200, Beijing Antai Yongxin Medical Technology Co., Ltd).

**In vivo antitumor efficacy evaluation**

To establish MSS-CRLM mouse model, C57BL/6 mice were intraperitoneally anesthetized with 50mg/kg of pentobarbital. Subsequently, the skin was aseptically prepared and midline laparotomy was conducted following abdominal shaving. Subsequent to these procedures, $3\times10^5$ cmt93-luc cells were injected via the superior mesenteric vein (SMV). Ultimately, the abdominal muscle and skin were meticulously sutured in multiple layers.

For evaluating the antitumor efficacy of neoantigen vaccine alone and combinational treatment in vivo, the MSS-CRLM model was divided into four groups (n=5). The mice in each group were subcutaneously injected with identified neoantigen peptides (10μg/peptide) mixed with 50μg Poly(I:C) in a 200μl volume on days 0, 4, and 8. Regorafenib (Bayer, 73-4506) was administered by oral gavage every two days from day 0, a total of 7 times at a dose of 10 mg/kg in 34% PEG400 (Sigma-Aldrich), 12% pluronic F68 (Thermmo Fischer Scientific, Waltham, Massachusetts, USA), and 20% water per manufacturer's recommendation. Mice treated with PBS served as the control group. Tumor burden in the MSS-CRLM model was assessed at 15-day intervals using the IVIS Spectrum animal imaging system (PerkinElmer, USA) after intraperitoneal injection of fluorescent substrates (D-luciferin potassium salt, XenolightTM) about 5 min, then the mice were exposed for 2s to obtain bioluminescence images. The mice were sacrificed 15 days after initial treatment for further analysis.

**Immunofluorescence**

The tumor isolated from mice was fixed in formalin and embedded in paraffin, as described previously. Sections measuring 4 μm in thickness were prepared on slides

and allowed to air dry at room temperature. Subsequently, the slides were subjected to blocking before being incubated with primary antibodies (rabbit anti-mouse CD4 mAb, Servicebio, GB13064-1; rabbit anti-mouse CD8 mAb, Servicebio, GB13068) overnight at 4°C, followed by secondary antibodies (Wuhan servicebio technology CO., Ltd.) for 30 minutes at room temperature. The resulting immuno-stained slides (5 slides per mouse; 3 mice) were digitally scanned at 10/40 magnification and cropped images were obtained by fluorescent microscope (NIKON ECLIPSE C1).

**Immunohistochemistry**

Tumors from each mouse were fixed in formalin and embedded in paraffin. Sections of 4-μm thickness were then prepared on slides and allowed to air dry at room temperature. Following blocking, the slides were incubated overnight at 4°C with a primary antibody (rabbit anti-mouse IFN-γ mAb, ThermoFisher, PA5-95560) and then for 30 minutes at room temperature with a secondary antibody (Fuzhou Maixin Biothech CO., Ltd). The resulting immuno-stained slides (5 slides per mouse; 3 mice) were digitally scanned at ×10/40 magnification and cropped images were obtained.

**HE staining**

HE staining was performed following standard procedures. Briefly, after deparaffinization and rehydration, tissue sections of the heart, liver, spleen, lung, and kidney were stained with hematoxylin solution for 5 minutes, followed by 5 dips in 1% acid ethanol and rinsing in distilled water. Subsequently, the sections were stained with eosin solution (Servicebio, G1001) for 3 minutes, dehydrated with graded alcohol, and cleared in xylene. The HE-stained slides (5 slides per mouse; 3 mice) were digitized

and cropped images were obtained.

**Tissue processing for flow cytometry**

Tumor tissues obtained from mice were sectioned and subsequently minced using surgical scissors on day 15 following the initiation of treatment. The minced specimens were then subjected to enzymatic digestion in 5 mL of RPMI-1640 medium, supplemented with 1mg/mL collagenase type II (Sigma, C6885) and 2 mg/mL DNase I (Sigma, DN25), at 37 °C for 1 hour with gentle agitation. Following digestion, an equivalent volume of cold flow buffer (PBS with 5% BSA) was introduced. The resulting mixture was filtered through a 40μm cell strainer, and the resulting single-cell suspension was centrifuged at 800g for 20 minutes before being resuspended in flow buffer at a concentration of $1\times10^7$ cells per 1mL. Tissue and mononuclear cells were isolated through the application of Ficoll density gradient centrifugation.

For spleen cells isolation, spleens were excised and mechanically disrupted with a 40μm syringe plunger. Mononuclear cells were subsequently separated using Ficoll density gradient centrifugation, followed by centrifugation at 800g for 5 minutes to obtain a single-cell suspension. The cells were then resuspended in flow buffer at a concentration of $1\times10^7$ cells per 1mL.

To isolate lymphocytes from the lymph nodes, the lymph nodes were mechanically disrupted using a 40μm cell strainer and a syringe plunger. The resulting single-cell suspension was then centrifuged at 800g for 5 minutes and subsequently resuspended in flow buffer at a concentration of $1\times10^7$ cells per 1mL.

**Flow cytometry and tetramer staining**

For flow cytometry analysis, single-cell suspensions from tumor (as described above), spleen and lymph nodes were stained in PBS (0.5% BSA) for 30 min with following antibodies for 45 min, rotating in the dark, including anti-mouse Rgs2 mAb (Invitrogen™, PA5-102486) plus APC-conjugated secondary antibody (Invitrogen™, A-865), anti-mouse CD3-APC/FITC mAb (eBioscience™, 17-0032-82/11-0037-42), anti-mouse CD8-PE/FITC mAb (eBioscience™, 12-0081-82/11-0081-82), anti-mouse CD137-APC mAb (eBioscience™, 17-1371-82), anti-mouse CD279-PE-Cyanine7 mAb (eBioscience™, 25-9985-82),and anti-mouse CD274-PE mAb (eBioscience™, 12-5983-42), anti-mouse MHC Class I (H-2$K^b$) - APC mAb (eBioscience™, 17-5958-82). Flow cytometry was performed on a flow cytometer (BD FACSVerseTM, USA) and data were analyzed using FlowJo v.10.

The anti-mouse peptide-specific tetramer-PE was prepared using the QuickSwithTM Quant Tetramer Kit (MBL International, TB-7400-K1) in accordance with the manufacturer's instructions. In brief, 50μl of Tetramer (50μg/mL) was combined with 1μl of peptide solution (10nM) and 1μl of proprietary Peptide Exchange Factor, and then incubated for 4 hours at room temperature in the dark. The efficiency of the exchange process was assessed using a flow cytometer (BD FACSVerse$^{TM}$, USA) as outlined in the accompanying manual, and validated peptide-MHC I-tetramers were stored at 4°C until needed. For tetramer staining, single-cell suspensions from tumors were blocked with mouse Cohn fraction IgG (20μl per sample) for 5 minutes and then stained with 20μl of anti-mouse Tetramer-PE for epitopes (Ecpas_L1064F: H-2$K^b$) at room temperature for 30 minutes. Following tetramer preincubation, cells were stained

with anti-mouse CD8-FITC monoclonal antibody (eBioscience™, 12-0081-82) for 20 minutes in the dark with rotation. Flow cytometry analysis was conducted using a BD FACSVerse™ flow cytometer in the USA, and data were analyzed using FlowJo v.10 software.

**Metastasis rechallenge experiments**

To investigate the long-term immune memory efficacy of combinational therapy, MSS-CRLM mice model was constructed as described above. Following treatment with CRC-NeoVAS plus regorafenib, mice were re-injected via SMV with cmt93 cells 45 days later. Tumor-burden mice treated with PBS underwent surgical resection of the tumor and served as controls. Tumor burden was monitored using the IVIS Spectrum animal imaging system.

**Flow cytometric sorting**

Single-cell suspensions (as described above) underwent Fc block with RPMI-1640 containing 5% BSA for 10 min on ice. Samples were then stained with 1:100 dilutions of anti-mouse CD3-FITC mAb, (eBioscience™, 17-0032-82), anti-mouse CD8-PE mAb (eBioscience™, 12-0081-82) for ELISPOT assay to test which T cell subset was activated by each neoantigen peptide, anti-human/anti-mouse CD3-FITC mAb, (eBioscience™, 11-0037-42/17-0032-82), anti-human/anti-mouse CD8-PE mAb (eBioscience™, 12-0088-42/12-0081-82) and anti-human/anti-mouse Rgs2 mAb (Invitrogen™, PA5-102486) plus APC-conjugated secondary antibody (Invitrogen™, A-865) for in vivo T cell cytotoxicity assay and adoptive cell therapy. Samples were stained in the dark for 30 min on ice, washed twice with flow buffer and resuspended

in PBS with 0.5% BSA. Sorting was performed on a Fusion cell sorter (BD FACSAriaTM, USA).

Single-cell suspensions were subjected to Fc block with RPMI-1640 containing 5% BSA for 10 minutes on ice, as outlined above. Subsequently, the samples were stained with 1:100 dilutions of anti-mouse CD3-FITC mAb (eBioscience™, 17-0032-82) and anti-mouse CD8-PE mAb (eBioscience™, 12-0081-82) for ELISPOT assay to determine the activated T cell subset for each neoantigen peptide. Additionally, staining with anti-human/anti-mouse CD3-FITC mAb (eBioscience™, 11-0037-42/17-0032-82), anti-human/anti-mouse CD8-PE mAb (eBioscience™, 12-0088-42/12-0081-82), and anti-human/anti-mouse Rgs2 mAb (Invitrogen™, PA5-102486) plus APC-conjugated second antibody (Invitrogen™, A-865) was performed for in vivo T cell cytotoxicity assay and adoptive cell therapy.

**Single-cell RNA sequencing (scRNA-seq) library construction and data analysis**

Tumor tissues obtained from the MSS-CRLM model following treatment with CRC-NeoVAS and/or regorafenib were processed for single-cell RNA sequencing (scRNA-seq) library preparation using the 10×Genomics Chromium Single Cell 5' Library & Gel Bead reagent kit and Chromium Single Cell V(D)J Enrichment Kit. The scRNA-seq libraries were generated using the 10×Genomics Chromium single cell 5' v2 reagent kit. Subsequently, the scRNA-seq reads obtained from the 10×Genomics platform were aligned to the reference genome (mm10) using Cell Ranger (v5.0.1, 10×Genomics) with default parameters. Following mapping, UMI reads and cells meeting specified criteria were subjected to additional filtration using Seurat (v4.0.3)

in R (v4.1.0). These criteria included: genes detected in fewer than 3 cells; cells with fewer than 500 detected genes; cells with more than 5000 detected genes; cells with over 5% of UMI reads mapped to mitochondrial genes; and cells with the highest expressed gene count corresponding to more than 20% of UMI reads.

Following the removal of low-quality cells and potential doublets, a total of 12417, 9828, 14089, and 10341 cells were selected for analysis in the context of combinational therapy, neoantigen vaccine treatment alone, regorafenib treatment alone, and a control group. To facilitate a comparative and visual analysis, 7726 cells from each group were randomly chosen for subsequent examination. Subsequently, gene expression levels were normalized utilizing the NormalizeData function employing the LogNormalize method. UMAP dimensionality reduction was performed, and Seurat functions FindNeighbors and FindClusters were utilized for cell clustering. The cell clusters were annotated utilizing the SingleR package (v1.6.1) with annotations derived from the combination of the Immgen and MouseRNAseq datasets. Subsequently, to investigate the heterogeneity of T cells, T cell cluster was isolated and subjected to re-clustering using the UMAP algorithm in Seurat.

**Single-cell V(D)J analysis**

The T-cell library underwent analysis using Cell Ranger (v5.0.1, 10× Genomics). The "vdj" command was employed to produce sequence annotations with default parameters utilizing the reference refdata-cellranger-vdj-GRCm38-alts-ensembl-5.0.0 obtained from the 10× Genomics website. Subsequently, the "filtered_contig_annotations.csv" file for each sample generated by Cell Ranger was

imported and reanalyzed using the R package scRepertoire (v1.3.2).

**T cell developmental trajectory**

The T cell lineage trajectory and the corresponding trajectory plot were determined using Monocle2. Following the extraction of T cell clusters, genes with expression detected in fewer than 10 cells were excluded. Subsequently, genes with a q-value < 0.01 in the 'differentialGeneTest' function were utilized to sequence the cells in pseudotime analysis.

**Cell-cell interaction analysis**

In order to ascertain potential cell-cell interactions between $Rgs2^{+}CD8^{+}$ T cell and other T cell clusters, we utilized Cellchat to identify statistically significant ligand-receptor pairs. Ligand-receptor pairs with a p-value less than 0.05 were deemed to have significant interactions between the two cell types.

**Prognostic assessment using TCGA data**

RNA-seq data and clinical information of patients with late-stage CRC from The Cancer Genome Atlas (TCGA) were obtained from the Genomic Data Commons (GDC) data portal. A total of 165 patients with transcriptome data for tumor samples were included in the analysis. Patients were categorized into two groups based on the median expression level of the Rgs2_CD8A two-gene signature, as evaluated by single-sample gene set enrichment analysis (ssGSEA). Survival analysis was conducted using Kaplan-Meier curves, and the significance of differences between the two groups was assessed using the log-rank test.

**In vitro T cell cytotoxicity assay on cmt93 cells.**

In order to evaluate the cytotoxic potential of $Rgs2^{+}CD8^{+}$ T cells in vitro, MSS-CRLM mice were treated with RegoNeo and sacrificed 15 days post-treatment. $Rgs2^{-}CD8^{+}$/$Rgs2^{+}CD8^{+}$ T cells were isolated from fresh tumor excisions using single-cell sorting. Subsequently, $Rgs2^{-}CD8^{+}$/$Rgs2^{+}CD8^{+}$ T cells ($3\times10^{4}$ cells per well) and cmt93 cells ($1\times10^{4}$ cells per well) were co-cultured in a 96-well plate with IL-2 (10ng/ml, R&D systems, MX2918061) at 37°C with 5% CO2 for 48 hours. The cells were harvested and co-stained with anti-mouse CD3-APC mAb (eBioscience™, 17-0031-82), annexin V-FITC, and Propidium Iodide at a concentration of 1.5 μg/mL to assess apoptosis in CD3-negative cells using a flow cytometer (BD FACSVerseTM, USA). The culture medium was filtered and collected for ELISA analysis of TNF-α (Boster, EK0527) and IFN-γ (Boster, EK0375) following the manufacturer's instructions.

**In vivo T cell therapy experiment**

In the in vivo T cell therapy experiment, an orthotopic HCC model was established according to the aforementioned protocol. Subsequently, tumor-bearing mice were administered with FACS-sorted $Rgs2^{+}CD8^{+}$ T cells and $Rgs2^{-}CD8^{+}$ T cells ($2\times10^{5}$ per mouse), while mice in the control group were treated with PBS. The T cells were isolated from tumor-bearing mice subjected to combinational therapy as previously outlined. Control mice receiving PBS treatment were included for comparison. All treated animals were given daily injections of $1\times10^{3}$ IU IL-2 infusion for a duration of 5 days. Tumor measurements were conducted in a double-blinded manner by an impartial investigator. The tumor burden was monitored by the IVIS Spectrum animal imaging system (PerkinElmer, USA).

**MSS-CRLM patient derived organoids (PDOs) generation**

Fresh tumor tissue samples were rinsed with Cancer Organoid Basal Medium (#B213152, bioGenous) until the supernatant was clear, then minced into small fragments, digested with Tumor Tissue Digestion Solution (#K601003, bioGenous) in SmartOrgan Dissociator (bioGenous) at 37 ℃ for 5~10 min. The digestion was terminated by adding 2~4% fetal bovine serum (FBS; #B118-500, Nobimpex). The supernatant was collected, passed through a 70 μm filter (#CSS013070, BIOFIL) and centrifuged. The erythrocytes were lysed with Red Blood Cell Lysis Solution (#E238010, bioGenous) and then centrifuged. The cell pellet was suspended in Organoid Culture ECM (#M315066, bioGenous) and plated into 24 well plates (#H803002, BDBIO). The organoids were overlaid with Human Colorectal Cancer Organoid Medium (#K2103-CR, bioGenous). Medium were changed every 2 days. The organoids can be passaged by Organoid Dissociation Solution (#E238001, bioGenous) or cryopreserved by Organoid Cryopreservation Medium (#E238023, bioGenous).

**In vitro T cell cytotoxicity assay on PDOs**

In order to evaluate the cytotoxic effects of $Rgs2^{+}CD8^{+}$ T cells on PDOs, $Rgs2^{-}CD8^{+}$ T /$Rgs2^{+}CD8^{+}$ T cells were isolated from fresh CRLM tumor samples obtained from patients. Low-adherence 96-well plates(#30096L, BDBIO) were coated with 20% ECM (30 μL/well, 37°C, 30 min). Organoids were harvested using ice-cold Cancer Organoid Basal Medium. Subsequently, $Rgs2^{-}CD8^{+}$ T /$Rgs2^{+}CD8^{+}$ T cells ($5\times10^{4}$ cells per well) and PDOs ($1\times10^{4}$ cells per well) were co-cultured for 48 hours in a 96-well plate with IL-2 (10ng/mL, R&D systems, MX2918061) and OrganoidpleX Medium

(150μL, #CO1233, bioGenous) at 37°C in a 5% $CO_2$ environment. Bright-field imaging were established on day 1 and day 3. On Day 3, SYTOX™ Green (1:1000; #S7020, invitrogen) was added (50 μL/well, 30 min dark incubation) for fluorescence and bright field images and viability assessment. Finally, photos were taken under a microscope at after fixed.

Written informed consent from MSS-CRLM patients was obtained in accordance with institutional review board approval for analysis. All studies involving human samples were conducted in compliance with ethical regulations and were preapproved by the Ethics Review Committee of the First Affiliated Hospital of Fujian Medical University (YAN[2023]110).

**Figure legends:**

**Figure S1. Immune response evaluation for Neoantigen peptides vaccine.** A. Tumor growth curves of each group (n=5) treated with PBS, neoantigen peptides alone, Poly(I:C) and Neo-CRCVAS. B. The histogram of ELISPOT assay demonstrating neoantigen-specific reactivity of splenic T cells against the pool of 7 neoantigen peptides in mice treated with PBS, Neoantigen peptides, Poly(I:C) and Neo-CRCVAS. Results are presented as mean ± SD. *p<0.05; **p<0.01; ***p<0.001; ****p<0.0001.

**Figure S2. Immune status changes of TME induced by Regorabenib.** A. The effect on PDOs' Apoptosis treated by regorafenib (5μM) with or without IFN-γ exposure (1ng/ml) for 48 hours. B. Flow cytometry analysis showing the percentage of tumor cells expressing PD-L1 caused by regorafenib with or without IFN-γ exposure for 24 hours in each group (n=3). C. Flow cytometry analysis showing the percentage of MHC1 expression on tumor cells caused by regorafenib with or without IFN-γ exposure for 24 hours in each group (n=3). The statistical analysis was performed with ANOVA analysis. Results are presented as mean ± SD. *p<0.05; **p<0.01; ***p<0.001; ****p<0.0001.

**Figure S3. Tumor therapeutic effect of RegoNeo treatment in MSS CRLKM mice model.** A. Tumor growth curves of each group (n=5) treated with PBS, Neo-CRCVAS alone, regorafineb alone and RegoNeo. B. Tumor weight of each group treated with PBS, Neo-CRCVAS alone, regorafineb alone and RegoNeo at the end of the experiment or when mice died spontaneously (n=5). B. ELISPOT assay quantifying the neoantigen-specific IFN-γ secretion by splenic T cells from each group against 7 neoantigen

peptides (n=3). C. The absolute number of Ecpas_L1064F:H-$2K^b$ specific $CD8^+$ T cells in infiltrating $CD8^+$ T cells in the different treatment groups. The statistical analysis was performed with ANOVA analysis. Results are shown as mean ± SD. *p<0.05; **p<0.01; ***p<0.001; ****p<0.0001.

**Figure S4. In vivo safety evaluation of RegoNeo treatment.** A-M. Blood plasma analysis for ALB (albumin), TBIL (total bilirubin), ALT (alanine aminotransferase), AST (aspartate aminotransferase), γ-GGT (Gamma-glutamyltransferase), AKP (alkaline phosphatase), CK (creatine kinase), Urea, SCr (serum creatinine), Glu (glucose), TG (triglyceride), TCHO (total cholesterol) during treatment for each group (n=5), N. HE staining for heart, liver, spleen, lung and kidney from mice treated with RegoNeo, with scare bars of 100μm (10×) and 25μm (40×). O. Body weight changes during treatment for all groups (n=5). The statistical analysis was performed using ANOVA analysis, Results are presented as mean ± SD.

**Figure S5. Long-term immune memory induced by RegoNeo treatment.** A. The representative immunofluorescence image of $CD4^+$ and $CD8^+$ T cell infiltration in tumor tissues at each treated group, Scare bars, 100μm (10×), 50μm (20×). B. The histogram of absolute number of Ecpas_L1064F:H-$2K^b$ specific $CD8^+$ T cells in CRLM tumor tissue blood at 30 days after metastasis rechallenge. C. ELISPOT analysis showing the neoantigen specific-reactivity of splenic T cells against neoantigen pools from RegoNeo therapy treated mice at the 50th day after treatment. The statistical analysis was performed with ANOVA analysis, n=3 for all the groups. Results are shown as mean ± SD. *p<0.05; **p<0.01; ***p<0.001; ****p<0.0001.

**Figure S6. Tumor immune microenvironment landscape profiling via scRNA-seq data.** A. The histogram showing the proportion of different of clusters within each treatment group. B. The histogram showing the proportion of all T clusters within each group. C. TCR Clonetype diversity Estimation of each T clusters using the inverse Simpson index. D. The histogram showing the number of shared TCR clonotypes in all T clusters. E. The histogram showing the expression of MIF in Rgs2$^+$CD8$^+$ T cells (left) and CD74 in B cells (right). The statistical analysis was performed with ANOVA analysis. Results are presented as mean ± SD. *p<0.05; **p<0.01; ***p<0.001; ****p<0.0001.

**Figure S7. Antitumor potential of Rgs2$^+$CD8$^+$ T cells in MSS-CRLM.** A. The infiltration of Rgs2$^+$ T cell in tumor tissues assessed by immunofluorescence stain at each treated group, Scare bars, 100μm (10×), 50μm (20 × ). B. The histograms of absolute number of Ecpas_L1064F:H-2K$^b$ specific CD8$^+$ T cells in infiltrating Rgs2$^+$CD8$^+$ TILs (n=3) after different treatments. C. Tumor growth curves of each group (n=5) treated with control, Rgs2$^+$CD8$^+$ TILs and Rgs2$^+$CD8$^+$ TILs. The statistical analysis was performed with ANOVA analysis. Results are presented as mean ± SD.

## Figure S1

**A**

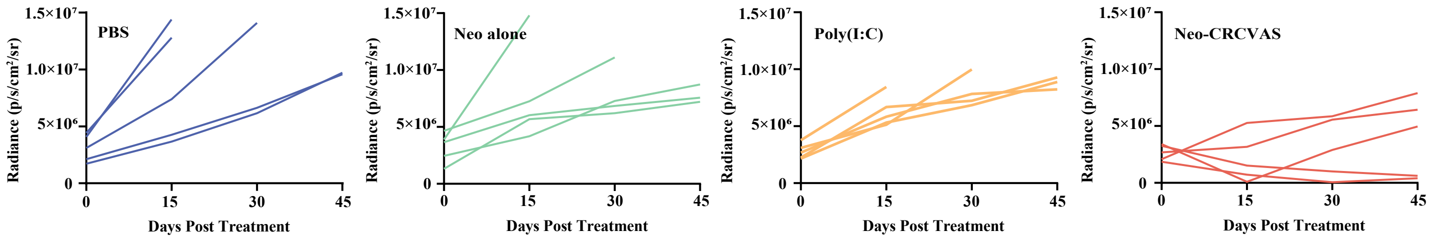
PBS
Neo alone
Poly(I:C)
Neo-CRCVAS
Radiance (p/s/cm²/sr)
1.5×10⁷
1.0×10⁷
5×10⁶
0
0
15
30
45
Days Post Treatment

**B**

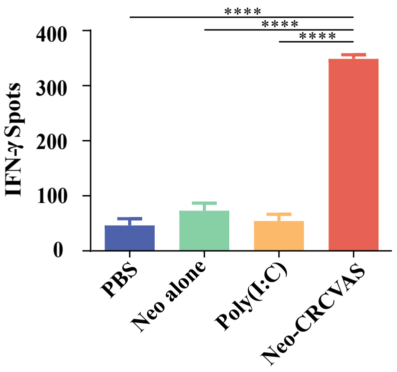
****
****
****
IFN-γ Spots
400
300
200
100
0
PBS
Neo alone
Poly(I:C)
Neo-CRCVAS

# Figure S2

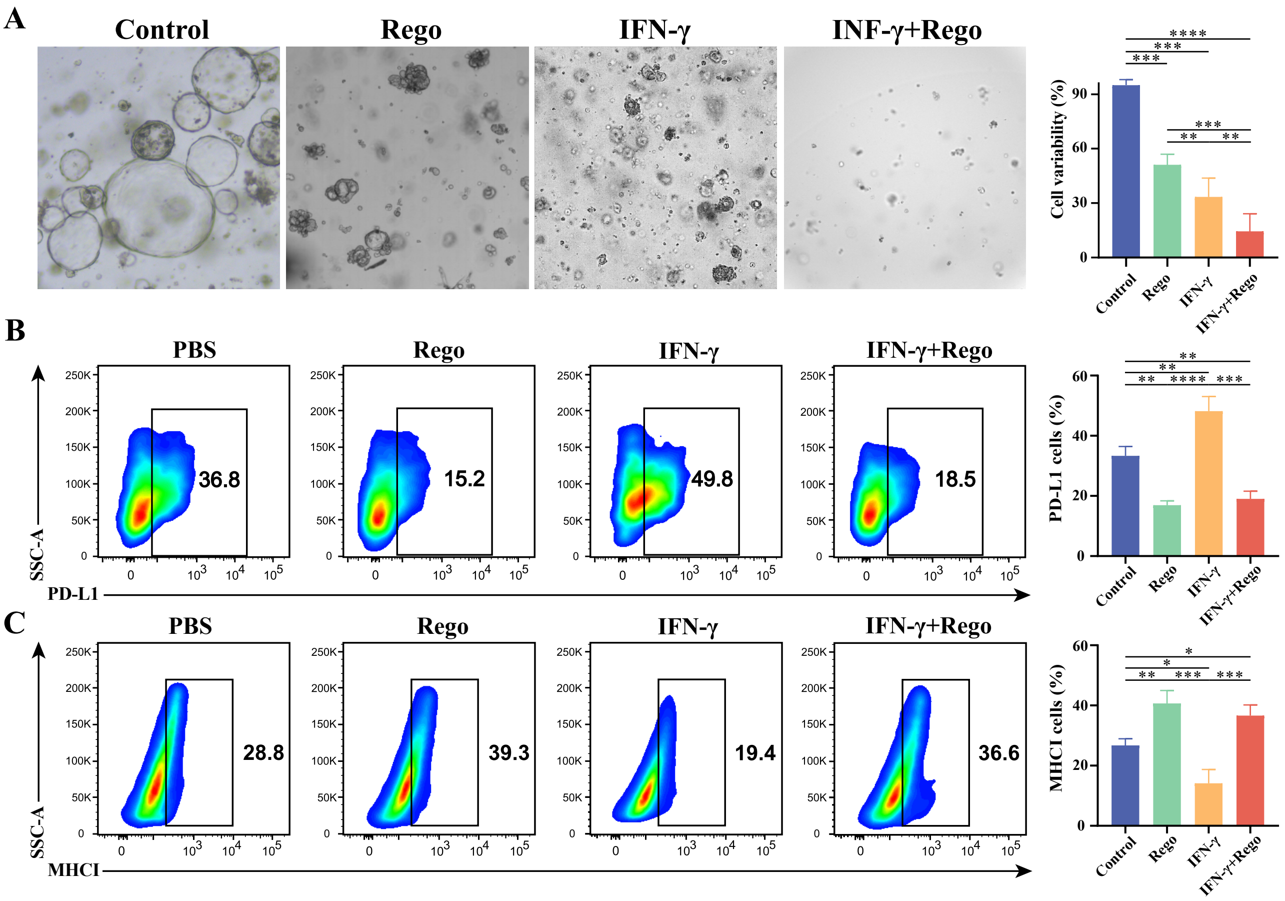
A
Control
Rego
IFN-γ
INF-γ+Rego
Cell variability (%)
Control
Rego
IFN-γ
IFN-γ+Rego
B
PBS
Rego
IFN-γ
IFN-γ+Rego
36.8
15.2
49.8
18.5
SSC-A
PD-L1
PD-L1 cells (%)
C
PBS
Rego
IFN-γ
IFN-γ+Rego
28.8
39.3
19.4
36.6
SSC-A
MHCI
MHCI cells (%)

# Figure S3

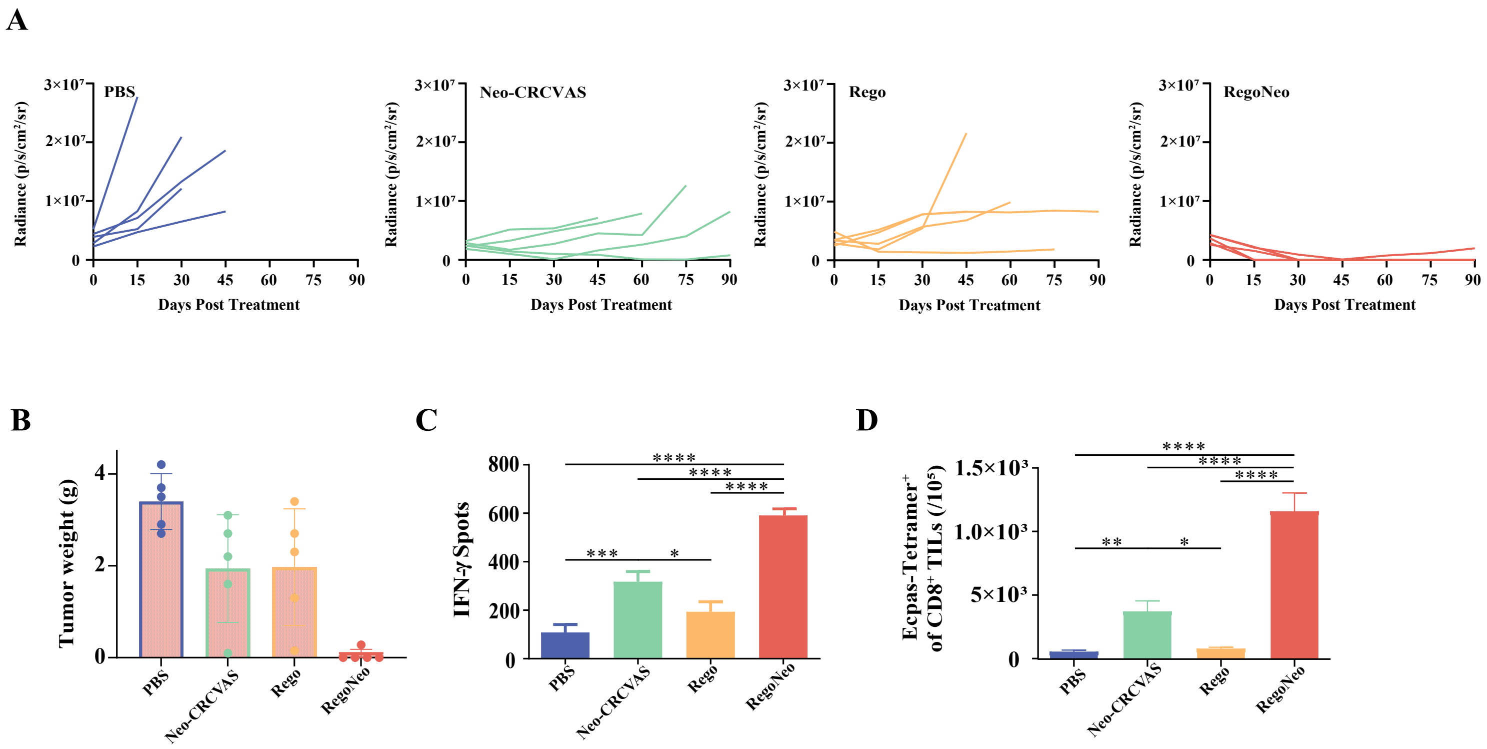
A
PBS
Neo-CRCVAS
Rego
RegoNeo
Radiance (p/s/cm²/sr)
Days Post Treatment
B
Tumor weight (g)
C
IFN-γ Spots
D
Ecpas-Tetramer+ of CD8+ TILs (/10⁵)
PBS
Neo-CRCVAS
Rego
RegoNeo

# Figure S4

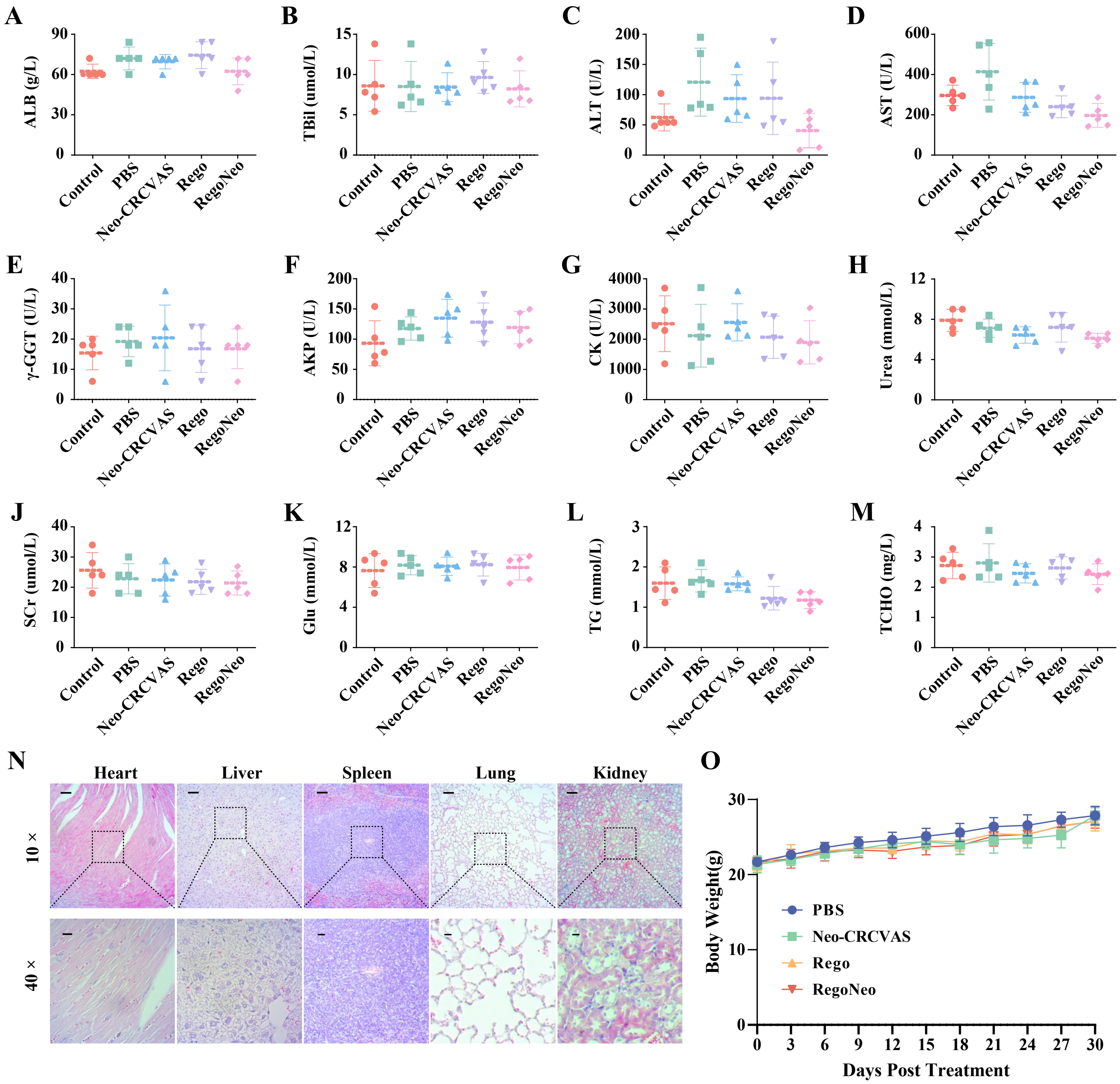
A
ALB (g/L)
B
TBil (umol/L)
C
ALT (U/L)
D
AST (U/L)
E
γ-GGT (U/L)
F
AKP (U/L)
G
CK (U/L)
H
Urea (mmol/L)
J
SCr (umol/L)
K
Glu (mmol/L)
L
TG (mmol/L)
M
TCHO (mg/L)
Control
PBS
Neo-CRCVAS
Rego
RegoNeo
N
Heart
Liver
Spleen
Lung
Kidney
10 ×
40 ×
O
Body Weight(g)
Days Post Treatment
PBS
Neo-CRCVAS
Rego
RegoNeo

# Figure S5

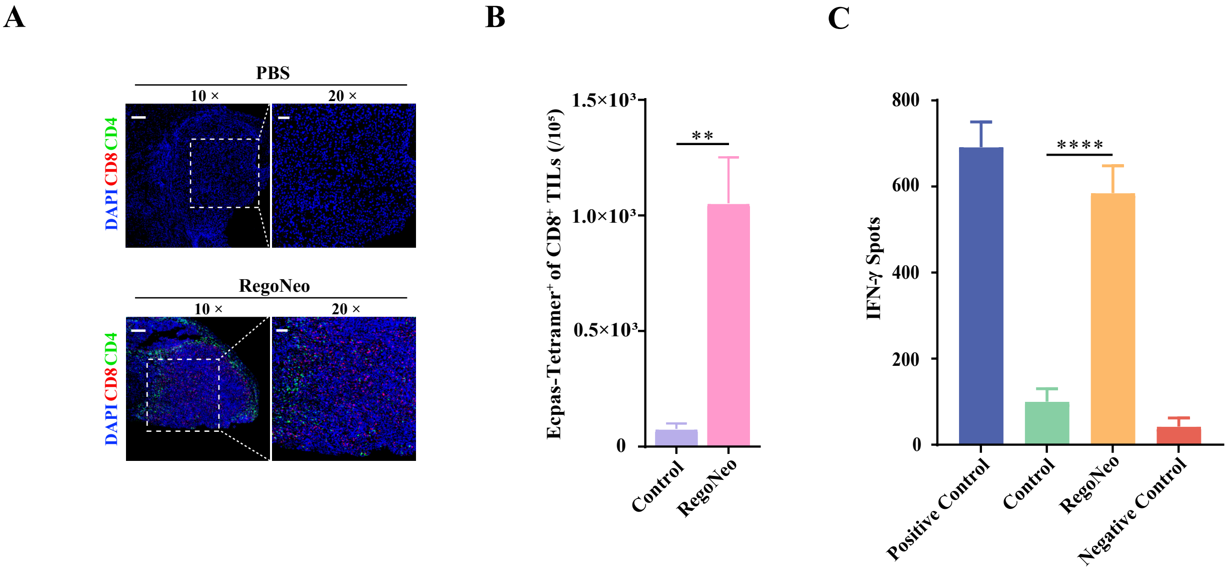
A
PBS
10 ×
20 ×
DAPI CD8 CD4
RegoNeo
10 ×
20 ×
DAPI CD8 CD4
B
Ecpas-Tetramer+ of CD8+ TILs (/10⁵)
1.5×10³
1.0×10³
0.5×10³
0
**
Control
RegoNeo
C
IFN-γ Spots
800
600
400
200
0
****
Positive Control
Control
RegoNeo
Negative Control

# Figure S6

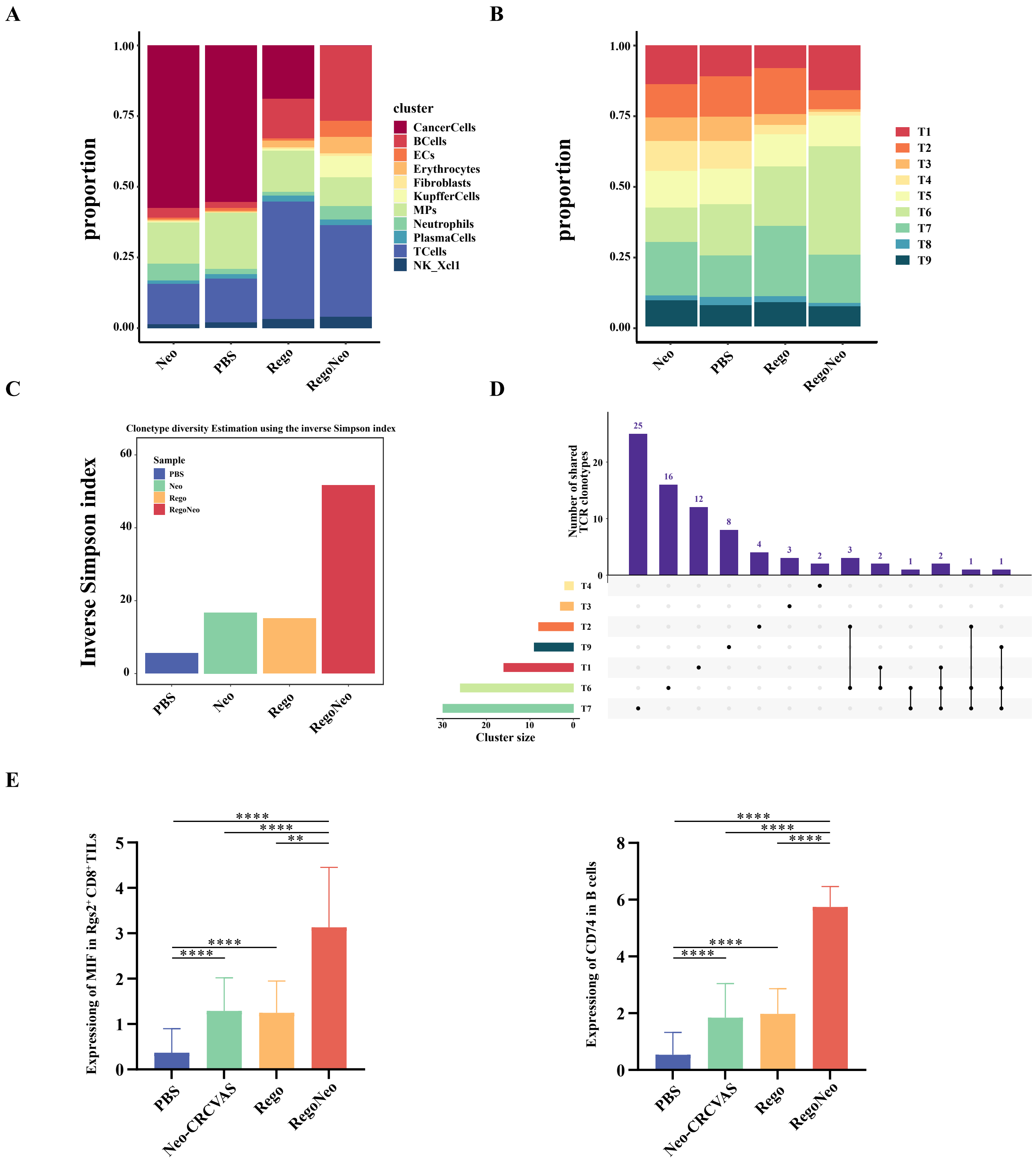
A
B
C
D
E
proportion
cluster
CancerCells
BCells
ECs
Erythrocytes
Fibroblasts
KupfferCells
MPs
Neutrophils
PlasmaCells
TCells
NK_Xcl1
Neo
PBS
Rego
RegoNeo
T1
T2
T3
T4
T5
T6
T7
T8
T9
Clonetype diversity Estimation using the inverse Simpson index
Inverse Simpson index
Sample
Number of shared TCR clonotypes
Cluster size
Expressiong of MIF in Rgs2+CD8+ TILs
Expressiong of CD74 in B cells
Neo-CRCVAS
****
**

# Figure S7

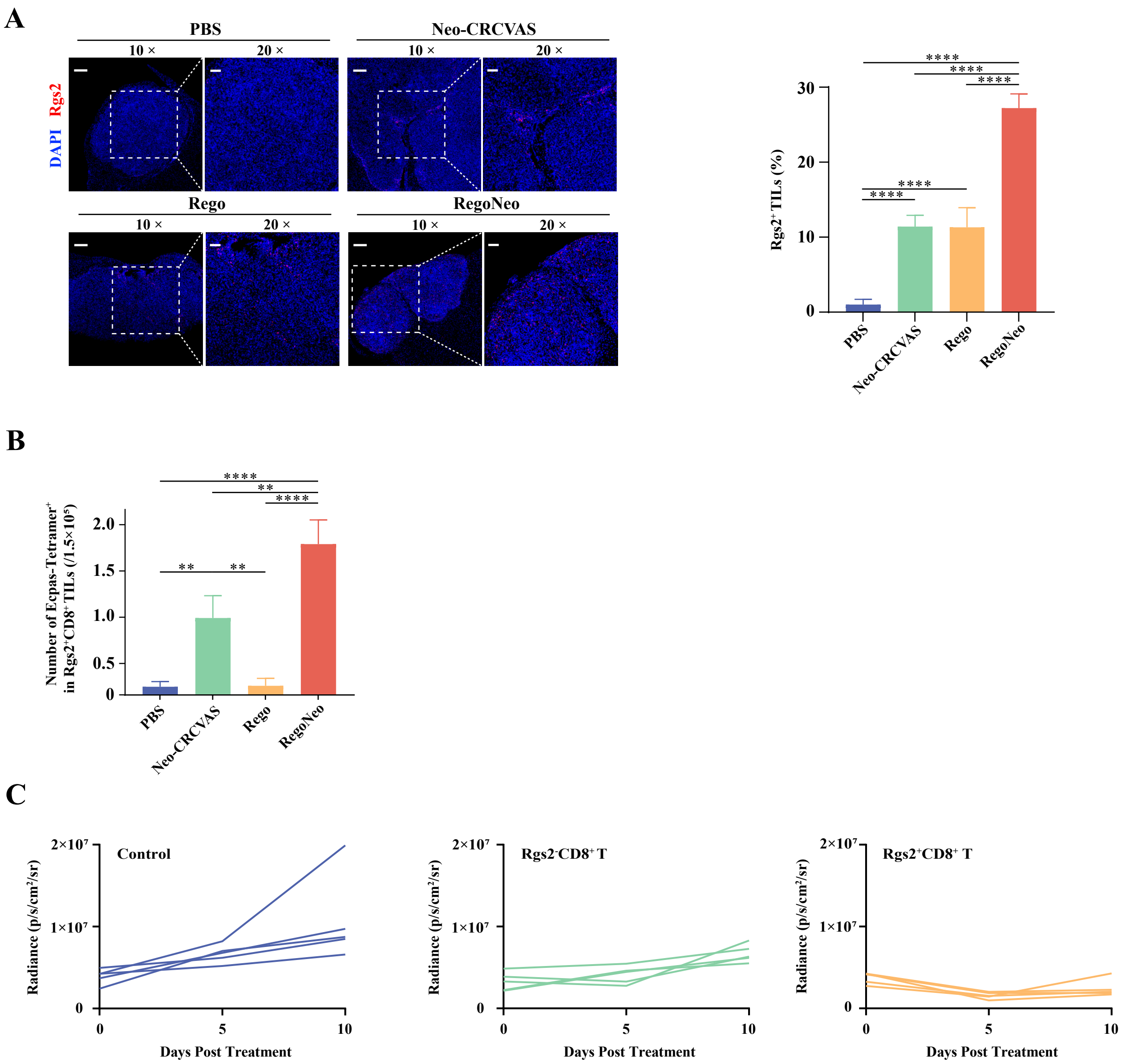

A
PBS
Neo-CRCVAS
Rego
RegoNeo
10 ×
20 ×
DAPI Rgs2
Rgs2⁺ TILs (%)
****
PBS
Neo-CRCVAS
Rego
RegoNeo
B
Number of Ecpas-Tetramer⁺ in Rgs2⁺CD8⁺ TILs (/1.5×10⁵)
****
**
C
Control
Rgs2⁻CD8⁺ T
Rgs2⁺CD8⁺ T
Radiance (p/s/cm²/sr)
2×10⁷
1×10⁷
Days Post Treatment

**Table S1. All exonic variants of cmt93 cells and C57BL/6 mouse tail**

| Chromosome | Position | Gene | Reference | Mutation | type |
|---|---|---|---|---|---|
| 1 | 9623925 | Adhfe1 | A | T | missense_variant |
| 1 | 10789863 | Cpa6 | G | A | synonymous_variant |
| 1 | 22635572 | Rims1 | G | T | synonymous_variant |
| 1 | 24224130 | Col9a1 | C | T | missense_variant |
| 1 | 26726352 | 31408C20R | TG | CT | missense_variant |
| 1 | 53231397 | Pms1 | G | A | synonymous_variant |
| 1 | 53952523 | Hecw2 | G | A | splice_region_variant,synonymous_variant |
| 1 | 58042076 | Sgo2a | G | C | synonymous_variant |
| 1 | 63190791 | Ndufs1 | A | G | missense_variant |
| 1 | 63584628 | Adam23 | G | A | synonymous_variant |
| 1 | 66657332 | Unc80 | G | A | synonymous_variant |
| 1 | 71340189 | Abca12 | G | A | missense_variant |
| 1 | 74172349 | Rufy4 | G | A | synonymous_variant |
| 1 | 74615336 | Zfp142 | G | A | synonymous_variant |
| 1 | 81275751 | Nyap2 | A | G | missense_variant |
| 1 | 88174911 | Mroh2a | G | A | missense_variant |
| 1 | 90888833 | Rab17 | C | T | splice_region_variant,synonymous_variant |
| 1 | 105931859 | Zcchc2 | G | A | missense_variant |
| 1 | 110847347 | Cdh19 | T | C | missense_variant |
| 1 | 127410348 | Mgat5 | C | T | synonymous_variant |
| 1 | 128118443 | R3hdm1 | C | A | synonymous_variant |
| 1 | 133016104 | Pik3c2b | C | T | synonymous_variant |
| 1 | 144016534 | Rgs13 | A | G | missense_variant |
| 1 | 151565311 | Niban1 | G | A | missense_variant |
| 1 | 153328205 | Shcbp1l | G | A | missense_variant |
| 1 | 154274226 | Cacna1e | C | T | synonymous_variant |
| 1 | 156144421 | Nphs2 | G | A | missense_variant |
| 1 | 159722665 | Tnr | T | G | missense_variant |
| 1 | 160777795 | Rc3h1 | C | T | missense_variant |
| 1 | 162873593 | Mroh9 | C | T | missense_variant |
| 1 | 165926571 | Dusp27 | C | A | stop_gained |
| 1 | 169351509 | Nuf2 | C | T | synonymous_variant |
| 1 | 171002011 | Pcp4l1 | G | A | missense_variant |
| 1 | 171230902 | Arhgap30 | C | T | missense_variant |
| 1 | 171956242 | Pex19 | CGACCAT | - | inframe_deletion |
| 1 | 172196845 | Kcnj10 | C | T | missense_variant |
| 1 | 172312397 | Igsf9 | G | A | missense_variant |
| 1 | 188582261 | Ush2a | G | C | missense_variant |
| 1 | 188596789 | Ush2a | G | A | missense_variant |
| 1 | 188596815 | Ush2a | G | A | missense_variant |
| 1 | 188643481 | Ush2a | G | A | missense_variant |
| 1 | 188643494 | Ush2a | T | C | synonymous_variant |
| 1 | 188643676 | Ush2a | T | C | missense_variant |
| 1 | 188643717 | Ush2a | A | G | missense_variant |
| 1 | 188644265 | Ush2a | T | A | synonymous_variant |
| 1 | 188644377 | Ush2a | T | A | missense_variant |
| 1 | 188679322 | Ush2a | C | T | synonymous_variant |
| 1 | 188694982 | Ush2a | C | T | missense_variant |
| 2 | 13480880 | Cubn | G | A | missense_variant |
| 2 | 25468911 | Ajm1 | G | A | synonymous_variant |
| 2 | 36887113 | Olfr357 | G | C | missense_variant |
| 2 | 59650623 | Tanc1 | G | A | missense_variant |
| 2 | 60142125 | Ly75 | A | C | missense_variant |
| 2 | 69655345 | Klhl23 | G | A | splice_donor_variant |

| | | | | | |
|---|---|---|---|---|---|
| 2 | 75506949 | Nfe2l2 | G | A | missense_variant |
| 2 | 75767009 | Ttc30b | T | C | synonymous_variant |
| 2 | 75807650 | Ttc30a2 | T | C | synonymous_variant |
| 2 | 75808360 | Ttc30a2 | GC | AT | synonymous_variant |
| 2 | 75811791 | Ttc30a1 | T | C | synonymous_variant |
| 2 | 75812034 | Ttc30a1 | C | T | synonymous_variant |
| 2 | 75877134 | Pde11a | C | T | synonymous_variant |
| 2 | 75988645 | Pde11a | C | T | missense_variant |
| 2 | 76045707 | Pde11a | A | G | synonymous_variant |
| 2 | 76045743 | Pde11a | T | C | synonymous_variant |
| 2 | 76168250 | Pde11a | G | A | synonymous_variant |
| 2 | 76209354 | Rbm45 | A | T | synonymous_variant |
| 2 | 76210844 | Rbm45 | G | A | synonymous_variant |
| 2 | 76601350 | Ttn | C | A | missense_variant |
| 2 | 76641021 | Ttn | G | C | missense_variant |
| 2 | 79172771 | Cerkl | A | G | missense_variant |
| 2 | 79469619 | Itprid2 | A | G | synonymous_variant |
| 2 | 79474997 | Itprid2 | A | G | missense_variant |
| 2 | 79475146 | Itprid2 | C | T | synonymous_variant |
| 2 | 79487934 | Itprid2 | C | A | synonymous_variant |
| 2 | 79490639 | Itprid2 | C | T | synonymous_variant |
| 2 | 79492048 | Itprid2 | C | G | splice_region_variant,synonymous_variant |
| 2 | 79492667 | Itprid2 | T | G | missense_variant |
| 2 | 79492781 | Itprid2 | A | G | synonymous_variant |
| 2 | 79492799 | Itprid2 | C | T | synonymous_variant |
| 2 | 79492994 | Itprid2 | A | G | synonymous_variant |
| 2 | 80161651 | Dnajc10 | C | G | synonymous_variant |
| 2 | 80243712 | Frzb | G | T | synonymous_variant |
| 2 | 80243716 | Frzb | G | A | missense_variant |
| 2 | 80248718 | Frzb | A | G | synonymous_variant |
| 2 | 80461364 | Dusp19 | GG | CA | missense_variant |
| 2 | 80476803 | Nup35 | A | G | synonymous_variant |
| 2 | 82087192 | Zfp804a | G | A | synonymous_variant |
| 2 | 82088517 | Zfp804a | A | G | missense_variant |
| 2 | 82815175 | Fsip2 | G | A | missense_variant |
| 2 | 83490551 | Zc3h15 | T | C | synonymous_variant |
| 2 | 83492609 | Zc3h15 | C | T | synonymous_variant |
| 2 | 83619377 | Itgav | T | G | synonymous_variant |
| 2 | 83622264 | Itgav | C | T | synonymous_variant |
| 2 | 83622312 | Itgav | C | T | synonymous_variant |
| 2 | 83622345 | Itgav | A | G | synonymous_variant |
| 2 | 83622354 | Itgav | T | C | synonymous_variant |
| 2 | 83622969 | Itgav | G | A | synonymous_variant |
| 2 | 83624589 | Itgav | T | C | synonymous_variant |
| 2 | 83625272 | Itgav | T | C | synonymous_variant |
| 2 | 83625287 | Itgav | T | C | synonymous_variant |
| 2 | 83627717 | Itgav | C | G | missense_variant |
| 2 | 83632180 | Itgav | G | T | missense_variant |
| 2 | 83688695 | Fam171b | G | A | synonymous_variant |
| 2 | 83709532 | Fam171b | G | A | synonymous_variant |
| 2 | 83709541 | Fam171b | T | C | synonymous_variant |
| 2 | 83709631 | Fam171b | G | A | synonymous_variant |
| 2 | 83710009 | Fam171b | G | A | synonymous_variant |
| 2 | 83710315 | Fam171b | G | A | synonymous_variant |
| 2 | 83710447 | Fam171b | C | T | synonymous_variant |
| 2 | 83710463 | Fam171b | A | C | synonymous_variant |

| | | | | | |
|---|---|---|---|---|---|
| 2 | 83710627 | Fam171b | C | T | synonymous_variant |
| 2 | 83710763 | Fam171b | C | A | synonymous_variant |
| 2 | 83745576 | Zswim2 | C | T | missense_variant |
| 2 | 83745581 | Zswim2 | T | C | missense_variant |
| 2 | 83745667 | Zswim2 | C | T | synonymous_variant |
| 2 | 83745753 | Zswim2 | T | C | missense_variant |
| 2 | 83745817 | Zswim2 | T | C | synonymous_variant |
| 2 | 84264596 | Tfpi | T | C | synonymous_variant |
| 2 | 84284313 | Tfpi | T | C | synonymous_variant |
| 2 | 84447182 | Ctnnd1 | T | G | synonymous_variant |
| 2 | 84521485 | Zdhhc5 | A | G | synonymous_variant |
| 2 | 84523728 | Zdhhc5 | G | A | synonymous_variant |
| 2 | 84595939 | Serping1 | A | T | synonymous_variant |
| 2 | 84600470 | Serping1 | A | G | missense_variant |
| 2 | 84633180 | Ube2l6 | G | T | missense_variant |
| 2 | 84645669 | Smtnl1 | G | A | missense_variant |
| 2 | 84646008 | Smtnl1 | G | A | synonymous_variant |
| 2 | 84647502 | Smtnl1 | T | C | synonymous_variant |
| 2 | 84649164 | Smtnl1 | A | G | missense_variant |
| 2 | 84660155 | Timm10 | G | C | synonymous_variant |
| 2 | 84660267 | Timm10 | T | C | synonymous_variant |
| 2 | 84888725 | Tnks1bp1 | C | G | missense_variant |
| 2 | 84889530 | Tnks1bp1 | C | T | missense_variant |
| 2 | 84892895 | Tnks1bp1 | A | G | missense_variant |
| 2 | 84893857 | Tnks1bp1 | C | T | synonymous_variant |
| 2 | 84967990 | Aplnr | T | C | synonymous_variant |
| 2 | 85410288 | Olfr996 | G | A | missense_variant |
| 2 | 85421621 | Olfr998 | T | G | missense_variant |
| 2 | 85438508 | Olfr1000 | C | A | missense_variant |
| 2 | 85439158 | Olfr1000 | A | - | frameshift_variant |
| 2 | 85478000 | Olfr1002 | C | T | missense_variant |
| 2 | 85493838 | Olfr154 | A | C | missense_variant |
| 2 | 85494192 | Olfr154 | T | A | missense_variant |
| 2 | 85494317 | Olfr154 | C | T | missense_variant |
| 2 | 85505340 | Olfr1006 | C | T | missense_variant |
| 2 | 85520138 | Olfr1008 | T | C | missense_variant |
| 2 | 85520211 | Olfr1008 | A | G | missense_variant |
| 2 | 85520424 | Olfr1008 | T | C | missense_variant |
| 2 | 85520491 | Olfr1008 | T | A | synonymous_variant |
| 2 | 85551912 | Olfr1009 | C | T | synonymous_variant |
| 2 | 85551999 | Olfr1009 | A | G | synonymous_variant |
| 2 | 85552044 | Olfr1009 | A | C | synonymous_variant |
| 2 | 85552215 | Olfr1009 | T | C | synonymous_variant |
| 2 | 85552575 | Olfr1009 | T | C | synonymous_variant |
| 2 | 85589963 | Olfr1012 | G | A | synonymous_variant |
| 2 | 85590125 | Olfr1012 | C | G | synonymous_variant |
| 2 | 85590626 | Olfr1012 | G | A | synonymous_variant |
| 2 | 85600580 | Olfr1013 | T | C | missense_variant |
| 2 | 85600596 | Olfr1013 | T | A | synonymous_variant |
| 2 | 85601019 | Olfr1013 | A | G | synonymous_variant |
| 2 | 85607008 | Olfr1014 | C | T | missense_variant |
| 2 | 85607707 | Olfr1014 | C | T | missense_variant |
| 2 | 85615889 | Olfr1015 | G | A | synonymous_variant |
| 2 | 85616225 | Olfr1015 | C | T | synonymous_variant |
| 2 | 85616286 | Olfr1015 | T | C | synonymous_variant |
| 2 | 85616615 | Olfr1015 | T | C | synonymous_variant |

| | | | | | |
|---|---|---|---|---|---|
| 2 | 85616692 | Olfr1015 | C | T | missense_variant |
| 2 | 85630027 | Olfr1016 | T | C | missense_variant |
| 2 | 85630127 | Olfr1016 | A | C | synonymous_variant |
| 2 | 85630268 | Olfr1016 | A | C | synonymous_variant |
| 2 | 85630601 | Olfr1016 | T | G | synonymous_variant |
| 2 | 85653333 | Olfr1018 | C | T | missense_variant |
| 2 | 85653454 | Olfr1018 | T | C | synonymous_variant |
| 2 | 85653508 | Olfr1018 | T | C | synonymous_variant |
| 2 | 85653603 | Olfr1018 | C | T | missense_variant |
| 2 | 85653743 | Olfr1018 | A | G | missense_variant |
| 2 | 85654033 | Olfr1018 | C | G | synonymous_variant |
| 2 | 85654072 | Olfr1018 | C | T | synonymous_variant |
| 2 | 85654132 | Olfr1018 | G | A | synonymous_variant |
| 2 | 85654251 | Olfr1018 | A | - | frameshift_variant,stop_lost |
| 2 | 85699302 | Olfr1022 | A | G | missense_variant |
| 2 | 85699695 | Olfr1022 | A | G | missense_variant |
| 2 | 85717181 | Olfr1023 | T | C | synonymous_variant |
| 2 | 85717188 | Olfr1023 | G | A | missense_variant |
| 2 | 85717325 | Olfr1023 | T | C | synonymous_variant |
| 2 | 85717829 | Olfr1023 | C | T | synonymous_variant |
| 2 | 85718027 | Olfr1023 | C | T | synonymous_variant |
| 2 | 85734432 | Olfr1024 | A | G | missense_variant |
| 2 | 85734668 | Olfr1024 | T | G | missense_variant |
| 2 | 85734976 | Olfr1024 | A | T | missense_variant |
| 2 | 85735016 | Olfr1024 | C | T | synonymous_variant |
| 2 | 85735094 | Olfr1024 | C | T | synonymous_variant |
| 2 | 85735371 | Olfr1024 | C | T | missense_variant |
| 2 | 85753977 | Olfr1026 | C | T | missense_variant |
| 2 | 85754267 | Olfr1026 | T | C | synonymous_variant |
| 2 | 85782314 | Olfr1028 | G | A | synonymous_variant |
| 2 | 85805703 | Olfr1029 | G | C | missense_variant |
| 2 | 85814491 | Olfr1030 | G | A | synonymous_variant |
| 2 | 85814620 | Olfr1030 | T | G | synonymous_variant |
| 2 | 85822483 | Olfr1031 | C | A | synonymous_variant |
| 2 | 85838923 | Olfr1032 | C | A | missense_variant |
| 2 | 85877410 | Olfr1034 | T | C | missense_variant |
| 2 | 85877607 | Olfr1034 | A | G | synonymous_variant |
| 2 | 85905449 | Olfr1036 | G | A | missense_variant |
| 2 | 85915724 | Olfr1037 | A | G | synonymous_variant |
| 2 | 85961578 | Olfr1039 | T | C | missense_variant |
| 2 | 85961742 | Olfr1039 | T | G | missense_variant |
| 2 | 85990134 | Olfr1042 | A | G | synonymous_variant |
| 2 | 85990277 | Olfr1042 | T | C | missense_variant |
| 2 | 85992779 | Olfr1043 | G | C | synonymous_variant |
| 2 | 86001488 | Olfr1044 | A | G | synonymous_variant |
| 2 | 86028175 | Olfr1045 | C | T | missense_variant |
| 2 | 86028198 | Olfr1045 | G | A | synonymous_variant |
| 2 | 86028333 | Olfr1045 | C | T | synonymous_variant |
| 2 | 86028630 | Olfr1045 | T | A | synonymous_variant |
| 2 | 86028657 | Olfr1045 | C | G | synonymous_variant |
| 2 | 86047111 | Olfr1046 | G | A | synonymous_variant |
| 2 | 86047526 | Olfr1046 | A | G | missense_variant |
| 2 | 86085402 | Olfr1049 | C | G | missense_variant |
| 2 | 86085519 | Olfr1049 | T | C | missense_variant |
| 2 | 86085702 | Olfr1049 | G | A | missense_variant |
| 2 | 86106196 | Olfr1051 | C | G | missense_variant |

| | | | | | |
|---|---|---|---|---|---|
| 2 | 86106251 | Olfr1051 | C | T | synonymous_variant |
| 2 | 86106771 | Olfr1051 | C | G | missense_variant |
| 2 | 86128623 | Olfr1052 | T | A | synonymous_variant |
| 2 | 86128819 | Olfr1052 | C | T | missense_variant |
| 2 | 86145068 | Olfr1053 | A | C | missense_variant |
| 2 | 86177583 | Olfr1055 | G | A | missense_variant |
| 2 | 86177825 | Olfr1055 | A | T | missense_variant |
| 2 | 86186142 | Olfr1056 | T | C | missense_variant |
| 2 | 86205311 | Olfr1057 | A | T | synonymous_variant |
| 2 | 86215976 | Olfr1058 | G | C | missense_variant |
| 2 | 86216336 | Olfr1058 | A | C | missense_variant |
| 2 | 86216464 | Olfr1058 | G | A | synonymous_variant |
| 2 | 86216638 | Olfr1058 | G | T | synonymous_variant |
| 2 | 86216674 | Olfr1058 | T | A | synonymous_variant |
| 2 | 86216749 | Olfr1058 | A | G | synonymous_variant |
| 2 | 86243639 | Olfr1061 | G | A | synonymous_variant |
| 2 | 86243644 | Olfr1061 | A | G | missense_variant |
| 2 | 86243901 | Olfr1061 | G | A | missense_variant |
| 2 | 86244183 | Olfr1061 | A | T | missense_variant |
| 2 | 86244239 | Olfr1061 | A | T | missense_variant |
| 2 | 86275734 | Olfr1065 | G | C | synonymous_variant |
| 2 | 86275968 | Olfr1065 | G | A | synonymous_variant |
| 2 | 86276115 | Olfr1065 | G | A | synonymous_variant |
| 2 | 86276136 | Olfr1065 | A | G | synonymous_variant |
| 2 | 86276277 | Olfr1065 | G | A | synonymous_variant |
| 2 | 86285721 | Olfr1066 | G | T | missense_variant |
| 2 | 86285756 | Olfr1066 | G | A | synonymous_variant |
| 2 | 86285796 | Olfr1066 | C | A | missense_variant |
| 2 | 86285802 | Olfr1066 | C | A | missense_variant |
| 2 | 86285809 | Olfr1066 | C | T | missense_variant |
| 2 | 86286139 | Olfr1066 | T | C | missense_variant |
| 2 | 86286142 | Olfr1066 | T | G | missense_variant |
| 2 | 86339168 | Olfr1076 | T | C | missense_variant |
| 2 | 86339209 | Olfr1076 | T | A | synonymous_variant |
| 2 | 86339216 | Olfr1076 | A | C | missense_variant |
| 2 | 86339596 | Olfr1076 | A | C | synonymous_variant |
| 2 | 86424342 | Olfr1082 | G | A | missense_variant |
| 2 | 86424386 | Olfr1082 | G | A | missense_variant |
| 2 | 86424393 | Olfr1082 | C | T | missense_variant |
| 2 | 86424608 | Olfr1082 | A | G | missense_variant |
| 2 | 86469186 | Olfr1084 | T | C | missense_variant |
| 2 | 86469401 | Olfr1084 | G | T | missense_variant |
| 2 | 86469406 | Olfr1084 | A | C | synonymous_variant |
| 2 | 86469526 | Olfr1084 | A | G | synonymous_variant |
| 2 | 86469645 | Olfr1084 | C | T | missense_variant |
| 2 | 86488098 | Olfr1085 | A | G | missense_variant |
| 2 | 86488651 | Olfr1085 | A | G | synonymous_variant |
| 2 | 86507448 | Olfr1086 | G | T | synonymous_variant |
| 2 | 86520979 | Olfr1087 | C | A | missense_variant |
| 2 | 86563236 | Olfr1089 | G | T | missense_variant |
| 2 | 86563335 | Olfr1089 | A | G | missense_variant |
| 2 | 86563590 | Olfr1089 | T | C | missense_variant |
| 2 | 86616264 | Olfr1093 | G | T | synonymous_variant |
| 2 | 86659586 | Olfr1094 | A | T | synonymous_variant |
| 2 | 86659589 | Olfr1094 | G | A | synonymous_variant |
| 2 | 86659787 | Olfr1094 | C | T | synonymous_variant |

| | | | | | |
|---|---|---|---|---|---|
| 2 | 86681576 | Olfr1095 | A | G | synonymous_variant |
| 2 | 86681587 | Olfr1095 | A | C | missense_variant |
| 2 | 86681711 | Olfr1095 | A | G | synonymous_variant |
| 2 | 86681764 | Olfr1095 | G | A | missense_variant |
| 2 | 86721050 | Olfr1097 | A | G | synonymous_variant |
| 2 | 86721502 | Olfr1097 | C | G | missense_variant |
| 2 | 86752932 | Olfr1098 | T | C | missense_variant |
| 2 | 86753056 | Olfr1098 | T | A | synonymous_variant |
| 2 | 86753111 | Olfr1098 | A | G | missense_variant |
| 2 | 86753824 | Olfr1098 | C | T | synonymous_variant |
| 2 | 86753827 | Olfr1098 | T | C | synonymous_variant |
| 2 | 86818824 | Olfr1101 | T | G | missense_variant |
| 2 | 86819142 | Olfr1101 | G | A | missense_variant |
| 2 | 86832329 | Olfr1102 | G | A | synonymous_variant |
| 2 | 86832350 | Olfr1102 | T | C | synonymous_variant |
| 2 | 86832542 | Olfr1102 | T | G | synonymous_variant |
| 2 | 86832598 | Olfr1102 | T | C | missense_variant |
| 2 | 86832665 | Olfr1102 | A | T | synonymous_variant |
| 2 | 86832719 | Olfr1102 | T | C | synonymous_variant |
| 2 | 86832969 | Olfr1102 | G | A | missense_variant |
| 2 | 86833115 | Olfr1102 | C | T | synonymous_variant |
| 2 | 86852643 | Olfr1104 | C | G | missense_variant |
| 2 | 86863629 | Olfr1105 | T | C | missense_variant |
| 2 | 86863921 | Olfr1105 | C | T | missense_variant |
| 2 | 86864243 | Olfr1105 | G | A | synonymous_variant |
| 2 | 86864279 | Olfr1105 | A | G | synonymous_variant |
| 2 | 86878814 | Olfr1106 | T | G | synonymous_variant |
| 2 | 86879186 | Olfr1106 | A | G | synonymous_variant |
| 2 | 86879313 | Olfr1106 | C | T | missense_variant |
| 2 | 86901896 | Olfr1107 | T | A | missense_variant |
| 2 | 86922831 | Olfr1109 | C | T | synonymous_variant |
| 2 | 86922951 | Olfr1109 | G | A | synonymous_variant |
| 2 | 86922966 | Olfr1109 | A | G | synonymous_variant |
| 2 | 86922984 | Olfr1109 | A | G | synonymous_variant |
| 2 | 86923017 | Olfr1109 | G | A | synonymous_variant |
| 2 | 86923044 | Olfr1109 | C | T | synonymous_variant |
| 2 | 86923083 | Olfr1109 | A | G | synonymous_variant |
| 2 | 86923212 | Olfr1109 | G | A | synonymous_variant |
| 2 | 86923223 | Olfr1109 | C | T | missense_variant |
| 2 | 86923360 | Olfr1109 | A | G | missense_variant |
| 2 | 86923609 | Olfr1109 | CC | AG | missense_variant |
| 2 | 86937800 | Olfr259 | A | G | synonymous_variant |
| 2 | 86937929 | Olfr259 | G | A | synonymous_variant |
| 2 | 86938028 | Olfr259 | C | T | synonymous_variant |
| 2 | 86938044 | Olfr259 | C | T | missense_variant |
| 2 | 86938149 | Olfr259 | A | T | missense_variant |
| 2 | 86938247 | Olfr259 | T | A | synonymous_variant |
| 2 | 86938390 | Olfr259 | G | A | missense_variant |
| 2 | 86938454 | Olfr259 | C | T | synonymous_variant |
| 2 | 86938589 | Olfr259 | A | G | synonymous_variant |
| 2 | 86938720 | Olfr259 | G | A | missense_variant |
| 2 | 86965881 | Olfr1110 | T | C | synonymous_variant |
| 2 | 86966538 | Olfr1110 | G | A | synonymous_variant |
| 2 | 86980170 | Olfr1111 | G | A | synonymous_variant |
| 2 | 86980545 | Olfr1111 | G | A | synonymous_variant |
| 2 | 86980574 | Olfr1111 | A | G | missense_variant |

| | | | | | |
|---|---|---|---|---|---|
| 2 | 86980937 | Olfr1111 | A | T | missense_variant |
| 2 | 87022903 | Olfr1112 | C | A | missense_variant |
| 2 | 87043431 | Olfr1113 | A | G | missense_variant |
| 2 | 87083144 | Olfr1115 | C | T | missense_variant |
| 2 | 87139482 | Olfr1118 | T | G | synonymous_variant |
| 2 | 87139625 | Olfr1118 | A | G | missense_variant |
| 2 | 87140044 | Olfr1118 | A | G | missense_variant |
| 2 | 88625622 | Olfr1201 | T | A | missense_variant |
| 2 | 89000643 | Olfr1225 | C | G | synonymous_variant |
| 2 | 89001060 | Olfr1225 | C | A | synonymous_variant |
| 2 | 89001159 | Olfr1225 | A | G | synonymous_variant |
| 2 | 89001303 | Olfr1225 | G | T | missense_variant |
| 2 | 89023667 | Olfr1226 | G | A | missense_variant |
| 2 | 89023786 | Olfr1226 | T | C | synonymous_variant |
| 2 | 89079129 | Olfr1228 | T | A | missense_variant |
| 2 | 89127271 | Olfr1230 | G | A | synonymous_variant |
| 2 | 89133201 | Olfr1231 | C | A | missense_variant |
| 2 | 89170213 | Olfr1233 | T | A | synonymous_variant |
| 2 | 89170639 | Olfr1233 | A | G | synonymous_variant |
| 2 | 89193619 | Olfr1234 | G | C | missense_variant |
| 2 | 89248108 | Olfr1239 | G | A | synonymous_variant |
| 2 | 89358095 | Olfr1243 | G | A | synonymous_variant |
| 2 | 89358402 | Olfr1243 | G | C | synonymous_variant |
| 2 | 89405156 | Olfr1245 | A | G | missense_variant |
| 2 | 89405451 | Olfr1245 | A | G | synonymous_variant |
| 2 | 89405775 | Olfr1245 | C | T | synonymous_variant |
| 2 | 89420624 | Olfr1246 | G | A | synonymous_variant |
| 2 | 89420721 | Olfr1246 | G | A | missense_variant |
| 2 | 89420796 | Olfr1246 | T | C | missense_variant |
| 2 | 89421321 | Olfr1246 | A | G | missense_variant |
| 2 | 89439998 | Olfr1247 | C | T | synonymous_variant |
| 2 | 89440002 | Olfr1247 | A | C | missense_variant |
| 2 | 89440214 | Olfr1247 | G | C | synonymous_variant |
| 2 | 89460576 | Olfr1249 | A | G | missense_variant |
| 2 | 89460686 | Olfr1249 | A | G | synonymous_variant |
| 2 | 89487265 | Olfr1250 | A | G | synonymous_variant |
| 2 | 89487358 | Olfr1250 | C | A | missense_variant |
| 2 | 89487379 | Olfr1250 | A | G | synonymous_variant |
| 2 | 89487622 | Olfr1250 | G | T | synonymous_variant |
| 2 | 89487719 | Olfr1250 | T | G | missense_variant |
| 2 | 89551943 | Olfr1252 | C | T | missense_variant |
| 2 | 89582759 | Olfr1253 | C | A | missense_variant |
| 2 | 89646809 | Olfr1255 | C | T | synonymous_variant |
| 2 | 89666208 | Olfr1256 | A | G | missense_variant |
| 2 | 89674631 | Olfr48 | A | G | missense_variant |
| 2 | 89711754 | Olfr1257 | A | C | missense_variant |
| 2 | 89760168 | Olfr1258 | G | A | missense_variant |
| 2 | 89760172 | Olfr1258 | T | C | synonymous_variant |
| 2 | 89760226 | Olfr1258 | C | T | synonymous_variant |
| 2 | 89760311 | Olfr1258 | C | T | synonymous_variant |
| 2 | 89760406 | Olfr1258 | C | T | synonymous_variant |
| 2 | 89760544 | Olfr1258 | T | C | synonymous_variant |
| 2 | 89760642 | Olfr1258 | A | G | missense_variant |
| 2 | 89773813 | Olfr1259 | G | C | synonymous_variant |
| 2 | 89808212 | Olfr1260 | C | T | missense_variant |
| 2 | 89808726 | Olfr1260 | C | T | synonymous_variant |

| | | | | | |
|---|---|---|---|---|---|
| 2 | 89808981 | Olfr1260 | C | T | synonymous_variant |
| 2 | 89823932 | Olfr1261 | A | T | missense_variant |
| 2 | 89832967 | Olfr1262 | CA | AG | missense_variant |
| 2 | 89845869 | Olfr1263 | C | T | synonymous_variant |
| 2 | 89845938 | Olfr1263 | A | G | synonymous_variant |
| 2 | 89852051 | Olfr1264 | G | A | missense_variant |
| 2 | 89867710 | Olfr1265 | G | A | missense_variant |
| 2 | 89882103 | Olfr140 | A | G | synonymous_variant |
| 2 | 89882529 | Olfr140 | C | A | synonymous_variant |
| 2 | 89882568 | Olfr140 | G | A | synonymous_variant |
| 2 | 89949326 | Olfr1269 | C | T | synonymous_variant |
| 2 | 89968618 | Olfr32 | A | G | synonymous_variant |
| 2 | 89969398 | Olfr32 | G | A | synonymous_variant |
| 2 | 90082789 | Olfr142 | T | G | missense_variant |
| 2 | 90082856 | Olfr142 | T | C | missense_variant |
| 2 | 90083210 | Olfr142 | T | C | missense_variant |
| 2 | 90096692 | Olfr1271 | C | G | synonymous_variant |
| 2 | 90096755 | Olfr1271 | G | A | synonymous_variant |
| 2 | 90112645 | Olfr1272 | G | A | synonymous_variant |
| 2 | 90112648 | Olfr1272 | G | A | synonymous_variant |
| 2 | 90280162 | Ptprj | C | T | synonymous_variant |
| 2 | 90283397 | Ptprj | G | A | synonymous_variant |
| 2 | 90581887 | Fnbp4 | C | T | missense_variant |
| 2 | 90598951 | Fnbp4 | A | C | missense_variant |
| 2 | 90736551 | Kbtbd4 | G | A | synonymous_variant |
| 2 | 90739544 | Kbtbd4 | A | G | missense_variant |
| 2 | 90872294 | Rapsn | G | A | synonymous_variant |
| 2 | 90884617 | Psmc3 | A | G | missense_variant |
| 2 | 90888417 | Psmc3 | A | G | synonymous_variant |
| 2 | 90943685 | Spi1 | T | C | synonymous_variant |
| 2 | 90953257 | Mybpc3 | A | G | synonymous_variant |
| 2 | 90953263 | Mybpc3 | G | A | synonymous_variant |
| 2 | 90954283 | Mybpc3 | T | C | synonymous_variant |
| 2 | 90954304 | Mybpc3 | T | A | synonymous_variant |
| 2 | 90954316 | Mybpc3 | C | T | synonymous_variant |
| 2 | 90959360 | Mybpc3 | A | G | missense_variant |
| 2 | 90961206 | Mybpc3 | A | G | synonymous_variant |
| 2 | 90961251 | Mybpc3 | C | T | synonymous_variant |
| 2 | 90968672 | Madd | A | G | synonymous_variant |
| 2 | 90982841 | Madd | G | A | synonymous_variant |
| 2 | 91014762 | Nr1h3 | A | G | synonymous_variant |
| 2 | 91015056 | Nr1h3 | G | A | synonymous_variant |
| 2 | 91036195 | Acp2 | A | G | missense_variant |
| 2 | 91105487 | Arfgap2 | A | G | missense_variant |
| 2 | 91318882 | Lrp4 | T | C | synonymous_variant |
| 2 | 91324411 | Lrp4 | G | C | missense_variant |
| 2 | 91325202 | Lrp4 | G | A | synonymous_variant |
| 2 | 91325362 | Lrp4 | C | G | missense_variant |
| 2 | 91325385 | Lrp4 | T | C | synonymous_variant |
| 2 | 91338862 | Lrp4 | T | C | synonymous_variant |
| 2 | 91342034 | Lrp4 | G | A | synonymous_variant |
| 2 | 91342073 | Lrp4 | C | T | synonymous_variant |
| 2 | 92161106 | Phf21a | C | G | missense_variant |
| 2 | 92196854 | Large2 | A | G | synonymous_variant |
| 2 | 92199980 | Large2 | T | C | synonymous_variant |
| 2 | 92213884 | 700029I15R | G | A | missense_variant |

| | | | | | |
|---|---|---|---|---|---|
| 2 | 92217400 | Mapk8ip1 | G | C | synonymous_variant |
| 2 | 93642278 | Ext2 | G | T | missense_variant |
| 2 | 119124448 | Vps18 | C | T | synonymous_variant |
| 2 | 127547817 | Mall | G | A | synonymous_variant |
| 2 | 129305513 | :30045P16R | C | T | missense_variant |
| 2 | 131021669 | Cenpb | C | T | missense_variant |
| 2 | 132758011 | Fermt1 | C | T | missense_variant |
| 2 | 136379203 | Ankef1 | C | A | missense_variant |
| 2 | 140009984 | Esf1 | T | - | frameshift_variant |
| 2 | 146285929 | Ralgapa2 | A | G | splice_region_variant,synonymous_variant |
| 2 | 150081042 | Zfp937 | T | G | missense_variant |
| 2 | 150432040 | Apmap | G | A | synonymous_variant |
| 2 | 150480452 | Acss1 | G | T | stop_gained |
| 2 | 150594984 | Entpd6 | G | A | missense_variant |
| 2 | 151548914 | Tmem74b | C | A | missense_variant |
| 2 | 154219987 | Snta1 | G | A | missense_variant |
| 2 | 154391505 | '00003F12R | G | A | missense_variant |
| 2 | 162808938 | L3mbtl1 | G | A | missense_variant |
| 2 | 165356549 | Slc2a10 | G | A | missense_variant |
| 2 | 180657252 | Col20a1 | G | A | synonymous_variant |
| 2 | 180863776 | Fndc11 | G | A | missense_variant |
| 3 | 5307852 | Zfhx4 | C | T | synonymous_variant |
| 3 | 20369799 | Agtr1b | C | G | missense_variant |
| 3 | 33008575 | Pex5l | C | T | synonymous_variant |
| 3 | 36644809 | Bbs7 | C | T | missense_variant |
| 3 | 40888107 | Abhd18 | C | T | missense_variant |
| 3 | 45335642 | Pcdh10 | G | A | missense_variant |
| 3 | 49709412 | Pcdh18 | C | T | synonymous_variant |
| 3 | 54714692 | Rfxap | C | T | synonymous_variant |
| 3 | 74979023 | Zbbx | A | C | missense_variant |
| 3 | 74979030 | Zbbx | C | T | synonymous_variant |
| 3 | 74979061 | Zbbx | A | G | missense_variant |
| 3 | 75019507 | Zbbx | T | C | missense_variant |
| 3 | 75165077 | Serpini2 | A | G | missense_variant |
| 3 | 75521921 | Serpini1 | C | T | synonymous_variant |
| 3 | 75810617 | Golim4 | T | G | missense_variant |
| 3 | 76536283 | Fstl5 | C | G | missense_variant |
| 3 | 76615459 | Fstl5 | C | T | missense_variant |
| 3 | 78976341 | Rapgef2 | T | C | synonymous_variant |
| 3 | 78990630 | Rapgef2 | C | T | splice_acceptor_variant |
| 3 | 79005545 | Rapgef2 | A | G | synonymous_variant |
| 3 | 79388105 | Fnip2 | C | T | synonymous_variant |
| 3 | 79793584 | Gask1b | T | C | synonymous_variant |
| 3 | 81082162 | Pdgfc | T | C | synonymous_variant |
| 3 | 81868816 | Tdo2 | C | T | synonymous_variant |
| 3 | 81875542 | Tdo2 | T | A | synonymous_variant |
| 3 | 81882720 | Tdo2 | C | A | missense_variant |
| 3 | 82002010 | Gucy1a1 | T | C | missense_variant |
| 3 | 82013306 | Gucy1a1 | C | T | synonymous_variant |
| 3 | 82278537 | Map9 | A | G | missense_variant |
| 3 | 82281497 | Map9 | T | C | missense_variant |
| 3 | 82281504 | Map9 | A | G | missense_variant |
| 3 | 82771930 | Rbm46 | A | G | synonymous_variant |
| 3 | 82915257 | Fgg | C | G | missense_variant |
| 3 | 82917419 | Fgg | A | G | synonymous_variant |
| 3 | 82921488 | Fgg | C | T | synonymous_variant |

| | | | | | |
|---|---|---|---|---|---|
| 3 | 82935879 | Fga | A | G | synonymous_variant |
| 3 | 82936535 | Fga | C | T | synonymous_variant |
| 3 | 82938899 | Fga | G | A | missense_variant |
| 3 | 82951106 | Fgb | CGC | TGA | missense_variant |
| 3 | 82951118 | Fgb | G | A | synonymous_variant |
| 3 | 82951133 | Fgb | G | A | synonymous_variant |
| 3 | 82951563 | Fgb | C | T | synonymous_variant |
| 3 | 82954154 | Fgb | G | A | missense_variant,splice_region_variant |
| 3 | 83035133 | Gm10710 | C | G | synonymous_variant |
| 3 | 83744831 | Tlr2 | A | G | synonymous_variant |
| 3 | 83745836 | Tlr2 | C | A | missense_variant |
| 3 | 83840152 | Tmem131l | A | C | missense_variant |
| 3 | 84099487 | Trim2 | A | G | synonymous_variant |
| 3 | 84115622 | Trim2 | A | G | synonymous_variant |
| 3 | 84362363 | Fhdc1 | G | A | splice_region_variant,synonymous_variant |
| 3 | 84426874 | Arfip1 | G | T | stop_gained |
| 3 | 84426891 | Arfip1 | T | C | synonymous_variant |
| 3 | 84427002 | Arfip1 | C | T | synonymous_variant |
| 3 | 84501351 | Tigd4 | A | G | synonymous_variant |
| 3 | 84811063 | Fbxw7 | A | G | missense_variant |
| 3 | 84862211 | Fbxw7 | A | G | synonymous_variant |
| 3 | 85901334 | Prss48 | C | T | missense_variant |
| 3 | 85907812 | Prss48 | C | T | missense_variant |
| 3 | 86045359 | Rps3a1 | G | A | synonymous_variant |
| 3 | 86046376 | Rps3a1 | A | G | synonymous_variant |
| 3 | 86048576 | Rps3a1 | G | A | synonymous_variant |
| 3 | 86132670 | Lrba | A | G | synonymous_variant |
| 3 | 86211640 | Lrba | C | T | missense_variant |
| 3 | 86217798 | Lrba | T | C | synonymous_variant |
| 3 | 86231093 | Lrba | G | A | synonymous_variant |
| 3 | 86255503 | Lrba | A | G | synonymous_variant |
| 3 | 86256111 | Lrba | AGCACCT | - | inframe_deletion |
| 3 | 86267180 | Lrba | C | A | synonymous_variant |
| 3 | 86827337 | Dclk2 | G | A | synonymous_variant |
| 3 | 87159999 | Fcrls | T | C | synonymous_variant |
| 3 | 87160002 | Fcrls | A | C | synonymous_variant |
| 3 | 87164640 | Fcrls | A | G | synonymous_variant |
| 3 | 87164696 | Fcrls | T | C | missense_variant |
| 3 | 87164790 | Fcrls | T | C | synonymous_variant |
| 3 | 87164802 | Fcrls | A | G | synonymous_variant |
| 3 | 87164826 | Fcrls | A | C | synonymous_variant |
| 3 | 87164880 | Fcrls | T | C | synonymous_variant |
| 3 | 87164895 | Fcrls | C | T | synonymous_variant |
| 3 | 87166770 | Fcrls | G | A | synonymous_variant |
| 3 | 87166872 | Fcrls | C | T | synonymous_variant |
| 3 | 87275052 | Cd5l | A | G | missense_variant |
| 3 | 87275817 | Cd5l | A | G | synonymous_variant |
| 3 | 87275838 | Cd5l | T | C | synonymous_variant |
| 3 | 87292146 | Fcrl1 | C | G | missense_variant |
| 3 | 87293039 | Fcrl1 | T | C | missense_variant |
| 3 | 87294060 | Fcrl1 | A | G | synonymous_variant |
| 3 | 87296680 | Fcrl1 | C | G | synonymous_variant |
| 3 | 87296690 | Fcrl1 | C | T | missense_variant |
| 3 | 87350973 | Fcrl5 | C | A | missense_variant |
| 3 | 87350980 | Fcrl5 | TTC | - | inframe_deletion |
| 3 | 87350987 | Fcrl5 | T | C | missense_variant |

| | | | | | |
|---|---|---|---|---|---|
| 3 | 87350991 | Fcrl5 | A | C | missense_variant |
| 3 | 87351000 | Fcrl5 | G | A | missense_variant |
| 3 | 87351087 | Fcrl5 | T | G | missense_variant |
| 3 | 87351533 | Fcrl5 | G | A | synonymous_variant |
| 3 | 87351571 | Fcrl5 | A | G | missense_variant |
| 3 | 87351614 | Fcrl5 | T | A | missense_variant |
| 3 | 87351638 | Fcrl5 | T | C | synonymous_variant |
| 3 | 87353692 | Fcrl5 | A | G | missense_variant |
| 3 | 87353770 | Fcrl5 | T | C | missense_variant |
| 3 | 87353783 | Fcrl5 | A | C | missense_variant |
| 3 | 87364415 | Fcrl5 | G | A | splice_region_variant,synonymous_variant |
| 3 | 87443647 | Etv3 | G | T | missense_variant |
| 3 | 87443778 | Etv3 | T | C | missense_variant |
| 3 | 87462258 | Etv3l | T | G | synonymous_variant |
| 3 | 87586980 | Gm6570 | G | T | missense_variant |
| 3 | 87587060 | Gm6570 | G | A | synonymous_variant |
| 3 | 87587156 | Gm6570 | G | A | synonymous_variant |
| 3 | 87587268 | Gm6570 | A | G | missense_variant |
| 3 | 87595229 | Arhgef11 | T | C | missense_variant |
| 3 | 87633783 | Arhgef11 | T | G | missense_variant |
| 3 | 87634766 | Arhgef11 | A | G | synonymous_variant |
| 3 | 87635275 | Arhgef11 | C | T | synonymous_variant |
| 3 | 87635837 | Arhgef11 | T | C | missense_variant |
| 3 | 87635839 | Arhgef11 | T | C | synonymous_variant |
| 3 | 87648327 | Lrrc71 | T | C | synonymous_variant |
| 3 | 87648336 | Lrrc71 | T | A | synonymous_variant |
| 3 | 87649934 | Lrrc71 | T | C | synonymous_variant |
| 3 | 87650643 | Lrrc71 | T | C | synonymous_variant |
| 3 | 87658451 | Pear1 | A | G | synonymous_variant |
| 3 | 87658553 | Pear1 | G | A | synonymous_variant |
| 3 | 87658766 | Pear1 | C | T | synonymous_variant |
| 3 | 87659173 | Pear1 | T | C | synonymous_variant |
| 3 | 87661883 | Pear1 | G | A | synonymous_variant |
| 3 | 87663237 | Pear1 | G | A | synonymous_variant |
| 3 | 87663422 | Pear1 | C | T | synonymous_variant |
| 3 | 87663434 | Pear1 | G | A | synonymous_variant |
| 3 | 87663966 | Pear1 | A | G | synonymous_variant |
| 3 | 87665347 | Pear1 | G | A | synonymous_variant |
| 3 | 87665394 | Pear1 | T | C | missense_variant |
| 3 | 87777184 | Prcc | C | T | synonymous_variant |
| 3 | 87821391 | Hdgf | C | T | synonymous_variant |
| 3 | 87830311 | Mrpl24 | C | G | synonymous_variant |
| 3 | 87839018 | Isg20l2 | - | AAG | inframe_insertion |
| 3 | 87839059 | Isg20l2 | A | G | missense_variant |
| 3 | 87839305 | Isg20l2 | C | T | missense_variant |
| 3 | 87839447 | Isg20l2 | A | G | synonymous_variant |
| 3 | 87839459 | Isg20l2 | T | C | synonymous_variant |
| 3 | 87878677 | Nes | C | G | synonymous_variant |
| 3 | 87879225 | Nes | G | T | missense_variant |
| 3 | 87882152 | Nes | A | G | missense_variant |
| 3 | 87882804 | Nes | A | G | synonymous_variant |
| 3 | 87882975 | Nes | C | T | synonymous_variant |
| 3 | 87883035 | Nes | A | G | synonymous_variant |
| 3 | 87883114 | Nes | A | T | missense_variant |
| 3 | 87883346 | Nes | T | A | missense_variant |
| 3 | 87883372 | Nes | G | A | missense_variant |

| | | | | | |
|---|---|---|---|---|---|
| 3 | 87883414 | Nes | G | A | missense_variant |
| 3 | 87883451 | Nes | C | T | missense_variant |
| 3 | 87883502 | Nes | T | C | missense_variant |
| 3 | 87883659 | Nes | G | A | synonymous_variant |
| 3 | 87883761 | Nes | G | A | missense_variant |
| 3 | 87884007 | Nes | C | A | missense_variant |
| 3 | 87884294 | Nes | TG | CA | missense_variant |
| 3 | 87884306 | Nes | A | G | missense_variant |
| 3 | 87884442 | Nes | A | C | missense_variant |
| 3 | 87884764 | Nes | A | G | missense_variant |
| 3 | 87885318 | Nes | G | A | synonymous_variant |
| 3 | 87885465 | Nes | - | GTG | inframe_insertion |
| 3 | 87885723 | Nes | A | G | synonymous_variant |
| 3 | 87885786 | Nes | G | A | synonymous_variant |
| 3 | 87885952 | Nes | G | A | missense_variant |
| 3 | 87885970 | Nes | G | A | missense_variant |
| 3 | 87886186 | Nes | C | T | missense_variant |
| 3 | 87886514 | Nes | C | T | missense_variant |
| 3 | 87886671 | Nes | C | A | synonymous_variant |
| 3 | 87887001 | Nes | T | A | synonymous_variant |
| 3 | 87887204 | Nes | G | A | missense_variant |
| 3 | 88154820 | Rhbg | A | G | missense_variant |
| 3 | 88543004 | Arhgef2 | A | C | synonymous_variant |
| 3 | 88543367 | Arhgef2 | T | C | synonymous_variant |
| 3 | 88545603 | Arhgef2 | T | C | synonymous_variant |
| 3 | 88545648 | Arhgef2 | G | A | synonymous_variant |
| 3 | 88546737 | Arhgef2 | G | A | synonymous_variant |
| 3 | 88546743 | Arhgef2 | A | G | synonymous_variant |
| 3 | 88549866 | Arhgef2 | C | T | synonymous_variant |
| 3 | 88550279 | Arhgef2 | T | A | synonymous_variant |
| 3 | 88550281 | Arhgef2 | T | C | missense_variant |
| 3 | 88550675 | Arhgef2 | A | G | synonymous_variant |
| 3 | 88669846 | Syt11 | C | T | synonymous_variant |
| 3 | 88761987 | Gon4l | C | T | missense_variant |
| 3 | 88802574 | Gon4l | G | T | missense_variant |
| 3 | 88820058 | Msto1 | A | T | missense_variant |
| 3 | 88873369 | Ash1l | C | T | missense_variant |
| 3 | 88873611 | Ash1l | G | A | synonymous_variant |
| 3 | 88888572 | Ash1l | C | T | synonymous_variant |
| 3 | 88909107 | Ash1l | C | T | missense_variant |
| 3 | 88914488 | Ash1l | C | G | missense_variant |
| 3 | 88950435 | Ash1l | T | C | synonymous_variant |
| 3 | 88950444 | Ash1l | T | C | synonymous_variant |
| 3 | 89077611 | Gm45927 | A | G | missense_variant |
| 3 | 89156433 | Krtcap2 | T | C | synonymous_variant |
| 3 | 89354710 | Pbxip1 | T | C | missense_variant |
| 3 | 89570024 | Kcnn3 | G | T | synonymous_variant |
| 3 | 89668421 | Chrnb2 | C | G | missense_variant |
| 3 | 89793246 | Il6ra | G | A | synonymous_variant |
| 3 | 89794474 | Il6ra | A | G | synonymous_variant |
| 3 | 89848526 | Atp8b2 | C | A | synonymous_variant |
| 3 | 89848667 | Atp8b2 | T | C | synonymous_variant |
| 3 | 89851158 | Atp8b2 | C | G | synonymous_variant |
| 3 | 89853488 | Atp8b2 | C | G | splice_region_variant,synonymous_variant |
| 3 | 89864346 | Atp8b2 | T | C | synonymous_variant |
| 3 | 90011528 | Nup210l | G | A | missense_variant |

| | | | | | |
|---|---|---|---|---|---|
| 3 | 90011538 | Nup210l | T | A | missense_variant |
| 3 | 90087478 | Nup210l | A | G | missense_variant |
| 3 | 90089261 | Nup210l | G | A | synonymous_variant |
| 3 | 90105436 | Nup210l | A | G | missense_variant |
| 3 | 90105462 | Nup210l | A | G | missense_variant |
| 3 | 90111053 | Nup210l | A | G | missense_variant |
| 3 | 90114659 | Nup210l | G | A | missense_variant |
| 3 | 90114685 | Nup210l | C | T | synonymous_variant |
| 3 | 90114712 | Nup210l | C | G | synonymous_variant |
| 3 | 90117698 | Nup210l | C | A | synonymous_variant |
| 3 | 90119120 | Nup210l | C | T | missense_variant |
| 3 | 90132869 | Rab13 | G | C | missense_variant |
| 3 | 90142741 | Jtb | T | C | synonymous_variant |
| 3 | 90142823 | Jtb | A | C | synonymous_variant |
| 3 | 90145197 | Creb3l4 | G | A | synonymous_variant |
| 3 | 90249226 | Gatad2b | ACA | - | inframe_deletion |
| 3 | 90363257 | Npr1 | A | G | synonymous_variant |
| 3 | 90372274 | Npr1 | A | C | synonymous_variant |
| 3 | 90372433 | Npr1 | C | T | synonymous_variant |
| 3 | 90372535 | Npr1 | A | G | synonymous_variant |
| 3 | 90435125 | S100a14 | G | A | synonymous_variant |
| 3 | 90521082 | S100a6 | C | T | synonymous_variant |
| 3 | 92626402 | Lce1f | C | T | missense_variant |
| 3 | 92626554 | Lce1f | C | T | synonymous_variant |
| 3 | 92696605 | Lce1j | A | C | missense_variant |
| 3 | 92731596 | Kprp | A | G | missense_variant |
| 3 | 92731601 | Kprp | CGT | TAC | missense_variant |
| 3 | 92731643 | Kprp | TA | CG | missense_variant |
| 3 | 92731648 | Kprp | A | G | synonymous_variant |
| 3 | 92732768 | Kprp | T | C | missense_variant |
| 3 | 92732919 | Kprp | T | G | missense_variant |
| 3 | 92900465 | Lce3f | G | C | synonymous_variant |
| 3 | 93109554 | Flg2 | T | C | synonymous_variant |
| 3 | 93110584 | Flg2 | T | A | missense_variant |
| 3 | 93110592 | Flg2 | C | T | synonymous_variant |
| 3 | 93110643 | Flg2 | A | G | synonymous_variant |
| 3 | 93110690 | Flg2 | C | G | missense_variant |
| 3 | 93111150 | Flg2 | C | A | synonymous_variant |
| 3 | 93121271 | Flg2 | T | C | missense_variant |
| 3 | 93121658 | Flg2 | - | CGGTCA | inframe_insertion |
| 3 | 93123465 | Flg2 | C | T | synonymous_variant |
| 3 | 93127221 | Flg2 | A | G | synonymous_variant |
| 3 | 93230139 | Hrnr | T | A | missense_variant |
| 3 | 93238921 | Hrnr | A | G | missense_variant |
| 3 | 93350721 | Tchh | A | G | missense_variant |
| 3 | 93378476 | Tchhl1 | A | G | synonymous_variant |
| 3 | 93378524 | Tchhl1 | T | C | synonymous_variant |
| 3 | 93378848 | Tchhl1 | G | T | missense_variant |
| 3 | 93558894 | Tdpoz2 | C | T | synonymous_variant |
| 3 | 93559315 | Tdpoz2 | G | T | missense_variant |
| 3 | 93559571 | Tdpoz2 | G | T | synonymous_variant |
| 3 | 93559639 | Tdpoz2 | G | T | missense_variant |
| 3 | 93559657 | Tdpoz2 | G | A | missense_variant |
| 3 | 93559785 | Tdpoz2 | G | A | synonymous_variant |
| 3 | 93559865 | Tdpoz2 | T | C | missense_variant |
| 3 | 93559917 | Tdpoz2 | C | T | synonymous_variant |

| | | | | | |
|---|---|---|---|---|---|
| 3 | 93577934 | Tdpoz1 | A | G | synonymous_variant |
| 3 | 93578090 | Tdpoz1 | A | G | synonymous_variant |
| 3 | 93704095 | Tdpoz4 | T | C | synonymous_variant |
| 3 | 93704330 | Tdpoz4 | G | A | missense_variant |
| 3 | 93704442 | Tdpoz4 | C | T | synonymous_variant |
| 3 | 93704525 | Tdpoz4 | A | G | missense_variant |
| 3 | 93704628 | Tdpoz4 | G | A | synonymous_variant |
| 3 | 93704638 | Tdpoz4 | C | T | synonymous_variant |
| 3 | 93704742 | Tdpoz4 | A | G | synonymous_variant |
| 3 | 93733331 | Tdpoz3 | C | T | missense_variant |
| 3 | 93733403 | Tdpoz3 | T | C | missense_variant |
| 3 | 93733472 | Tdpoz3 | C | T | missense_variant |
| 3 | 93733477 | Tdpoz3 | A | G | missense_variant |
| 3 | 94082863 | Spopfm2 | G | A | missense_variant |
| 3 | 94083800 | Spopfm2 | C | A | missense_variant |
| 3 | 94224741 | Them4 | C | T | synonymous_variant |
| 3 | 94249661 | Them5 | A | G | synonymous_variant |
| 3 | 94270870 | C2cd4d | C | G | synonymous_variant |
| 3 | 94270908 | C2cd4d | GG | CA | missense_variant |
| 3 | 94270961 | C2cd4d | C | A | missense_variant |
| 3 | 94271480 | C2cd4d | A | G | missense_variant |
| 3 | 94395507 | Celf3 | T | C | synonymous_variant |
| 3 | 94541819 | Tuft1 | T | G | synonymous_variant |
| 3 | 94542800 | Tuft1 | A | C | splice_region_variant,synonymous_variant |
| 3 | 94546327 | Tuft1 | T | G | synonymous_variant |
| 3 | 94606955 | Selenbp2 | C | T | synonymous_variant |
| 3 | 94611511 | Selenbp2 | T | C | synonymous_variant |
| 3 | 94669908 | Cgn | C | A | missense_variant |
| 3 | 94674505 | Cgn | A | G | synonymous_variant |
| 3 | 94677936 | Cgn | T | C | missense_variant |
| 3 | 94686589 | Cgn | T | A | missense_variant |
| 3 | 94781972 | Pogz | C | G | synonymous_variant |
| 3 | 94784643 | Pogz | A | C | synonymous_variant |
| 3 | 94786182 | Pogz | C | A | synonymous_variant |
| 3 | 94844258 | Selenbp1 | T | C | synonymous_variant |
| 3 | 94847456 | Selenbp1 | C | A | synonymous_variant |
| 3 | 94851849 | Selenbp1 | G | A | synonymous_variant |
| 3 | 94866408 | Rfx5 | G | A | missense_variant |
| 3 | 94892086 | Pi4kb | G | C | synonymous_variant |
| 3 | 95025984 | Vps72 | C | T | missense_variant |
| 3 | 95029722 | Vps72 | C | T | missense_variant |
| 3 | 95590622 | Adamtsl4 | G | A | synonymous_variant |
| 3 | 95591234 | Adamtsl4 | A | G | synonymous_variant |
| 3 | 95591405 | Adamtsl4 | A | G | synonymous_variant |
| 3 | 96488959 | Polr3gl | C | T | splice_donor_variant |
| 3 | 96761873 | Pdzk1 | A | G | missense_variant |
| 3 | 101822635 | Slc22a15 | A | T | missense_variant |
| 3 | 104562867 | Slc16a1 | C | T | synonymous_variant |
| 3 | 106057091 | Chil3 | A | G | synonymous_variant |
| 3 | 106480973 | Dram2 | C | A | missense_variant |
| 3 | 107462145 | Ubl4b | C | T | synonymous_variant |
| 3 | 108729182 | Stxbp3 | G | A | synonymous_variant |
| 3 | 122572610 | Pde5a | C | T | synonymous_variant |
| 3 | 126158632 | Arsj | C | T | synonymous_variant |
| 3 | 131337496 | Papss1 | G | T | synonymous_variant |
| 3 | 133191295 | Tet2 | G | T | synonymous_variant |

| | | | | | |
|---|---|---|---|---|---|
| 3 | 135027693 | Slc9b2 | C | T | synonymous_variant |
| 3 | 154533175 | Tnni3k | C | T | missense_variant |
| 4 | 8862489 | Chd7 | G | A | splice_acceptor_variant |
| 4 | 11234659 | Ints8 | C | T | missense_variant |
| 4 | 21873684 | Pnisr | C | G | missense_variant |
| 4 | 25213768 | Fhl5 | C | T | missense_variant |
| 4 | 32640741 | Casp8ap2 | C | T | synonymous_variant |
| 4 | 40688162 | Aptx | T | C | missense_variant |
| 4 | 43654802 | Hint2 | C | T | missense_variant |
| 4 | 45399785 | Slc25a51 | T | G | missense_variant |
| 4 | 45423999 | Shb | G | A | missense_variant |
| 4 | 46144793 | Ncbp1 | C | T | missense_variant |
| 4 | 48398182 | Invs | G | A | missense_variant |
| 4 | 58824790 | Ecpas | G | A | missense_variant |
| 4 | 63089660 | Kif12 | - | CTCCACC | inframe_insertion |
| 4 | 68680627 | Brinp1 | C | T | synonymous_variant |
| 4 | 74253008 | Kdm4c | G | A | missense_variant |
| 4 | 82228612 | Nfib | C | A | missense_variant |
| 4 | 86135213 | Adamtsl1 | T | C | missense_variant |
| 4 | 86150923 | Adamtsl1 | A | G | synonymous_variant |
| 4 | 86195354 | Adamtsl1 | G | A | synonymous_variant |
| 4 | 86208002 | Adamtsl1 | A | G | synonymous_variant |
| 4 | 86406129 | Saxo1 | C | T | synonymous_variant |
| 4 | 86494473 | Rraga | C | T | synonymous_variant |
| 4 | 86580213 | Plin2 | G | T | missense_variant |
| 4 | 86692722 | Dennd4c | T | C | synonymous_variant |
| 4 | 86709681 | Dennd4c | A | G | synonymous_variant |
| 4 | 86755646 | Dennd4c | G | A | missense_variant |
| 4 | 86774125 | Rps6 | T | G | synonymous_variant |
| 4 | 86909599 | Slc24a2 | C | A | synonymous_variant |
| 4 | 87758992 | Mllt3 | A | G | missense_variant |
| 4 | 88047236 | Focad | T | C | synonymous_variant |
| 4 | 88092917 | Focad | C | T | synonymous_variant |
| 4 | 88092991 | Focad | G | A | synonymous_variant |
| 4 | 88100898 | Focad | A | G | synonymous_variant |
| 4 | 88115089 | Focad | A | G | missense_variant |
| 4 | 88115189 | Focad | T | G | missense_variant |
| 4 | 88215297 | Focad | T | A | missense_variant |
| 4 | 88215328 | Focad | G | A | synonymous_variant |
| 4 | 88262839 | Focad | A | G | synonymous_variant |
| 4 | 88273665 | Focad | G | A | synonymous_variant |
| 4 | 88315263 | Focad | G | A | missense_variant |
| 4 | 88319322 | Focad | C | T | synonymous_variant |
| 4 | 88321629 | Focad | A | T | missense_variant |
| 4 | 88332502 | Hacd4 | C | T | stop_retained_variant |
| 4 | 88332522 | Hacd4 | A | G | synonymous_variant |
| 4 | 88353233 | Hacd4 | T | C | synonymous_variant |
| 4 | 94409316 | Caap1 | A | C | missense_variant |
| 4 | 95956499 | Cyp2j13 | G | A | synonymous_variant |
| 4 | 98393949 | Patj | G | C | missense_variant |
| 4 | 98409058 | Patj | G | A | synonymous_variant |
| 4 | 98409070 | Patj | A | G | synonymous_variant |
| 4 | 98555855 | Patj | G | A | missense_variant |
| 4 | 98625370 | L1td1 | G | A | missense_variant |
| 4 | 99632019 | Alg6 | G | A | synonymous_variant |
| 4 | 99632738 | Alg6 | T | C | missense_variant |

| | | | | | |
|---|---|---|---|---|---|
| 4 | 99632826 | Alg6 | T | A | synonymous_variant |
| 4 | 99670199 | Itgb3bp | C | T | synonymous_variant |
| 4 | 99818685 | Pgm1 | A | C | synonymous_variant |
| 4 | 99818706 | Pgm1 | T | C | synonymous_variant |
| 4 | 99818730 | Pgm1 | T | C | synonymous_variant |
| 4 | 99836066 | Pgm1 | G | A | synonymous_variant |
| 4 | 99836072 | Pgm1 | T | C | synonymous_variant |
| 4 | 99841274 | Pgm1 | G | A | missense_variant |
| 4 | 100343860 | Ube2u | T | C | synonymous_variant |
| 4 | 100343901 | Ube2u | A | G | missense_variant |
| 4 | 100809269 | Cachd1 | C | T | synonymous_variant |
| 4 | 100809272 | Cachd1 | A | G | synonymous_variant |
| 4 | 100821813 | Cachd1 | T | C | synonymous_variant |
| 4 | 100960123 | Raver2 | C | T | missense_variant |
| 4 | 100960223 | Raver2 | G | A | synonymous_variant |
| 4 | 100988504 | Raver2 | A | C | synonymous_variant |
| 4 | 100991066 | Raver2 | G | C | synonymous_variant |
| 4 | 101513488 | Leprot | A | G | missense_variant |
| 4 | 101602807 | Lepr | G | A | synonymous_variant |
| 4 | 101622110 | Lepr | T | C | synonymous_variant |
| 4 | 101622144 | Lepr | A | G | missense_variant |
| 4 | 101630038 | Lepr | T | C | synonymous_variant |
| 4 | 101646523 | Lepr | G | A | missense_variant |
| 4 | 101797647 | Pramel19 | A | C | missense_variant |
| 4 | 101798416 | Pramel19 | T | G | missense_variant |
| 4 | 101798518 | Pramel19 | G | C | missense_variant |
| 4 | 101845514 | Gm12789 | A | G | missense_variant |
| 4 | 102727771 | Sgip1 | C | T | synonymous_variant |
| 4 | 102778467 | Sgip1 | A | C | missense_variant |
| 4 | 102849704 | Tctex1d1 | C | T | synonymous_variant |
| 4 | 102849740 | Tctex1d1 | A | T | missense_variant |
| 4 | 102883724 | Insl5 | A | C | missense_variant |
| 4 | 102883746 | Insl5 | T | C | synonymous_variant |
| 4 | 102883803 | Insl5 | A | G | synonymous_variant |
| 4 | 102884039 | Insl5 | T | G | start_lost |
| 4 | 102986555 | Mier1 | C | A | synonymous_variant |
| 4 | 103019624 | Mier1 | C | G | synonymous_variant |
| 4 | 103088417 | 21539E11R | C | T | missense_variant |
| 4 | 103088450 | 21539E11R | C | T | missense_variant |
| 4 | 103088570 | 21539E11R | A | G | missense_variant |
| 4 | 103092933 | 21539E11R | T | C | synonymous_variant |
| 4 | 103100037 | 21539E11R | C | T | synonymous_variant |
| 4 | 103112771 | 21539E11R | G | A | splice_region_variant,synonymous_variant |
| 4 | 108129466 | Zyg11b | C | T | missense_variant |
| 4 | 108412759 | Tut4 | C | T | missense_variant |
| 4 | 108430068 | Prpf38a | C | T | missense_variant |
| 4 | 108482993 | Cc2d1b | C | T | missense_variant |
| 4 | 109181264 | Eps15 | G | A | splice_acceptor_variant |
| 4 | 113685059 | Skint5 | G | A | synonymous_variant |
| 4 | 114764630 | Foxd2 | G | A | synonymous_variant |
| 4 | 114782968 | Foxe3 | G | C | synonymous_variant |
| 4 | 114822209 | Cmpk1 | G | A | synonymous_variant |
| 4 | 114844250 | Cmpk1 | G | A | synonymous_variant |
| 4 | 114844258 | Cmpk1 | G | A | missense_variant |
| 4 | 114844263 | Cmpk1 | G | C | missense_variant |
| 4 | 114866557 | Stil | A | G | synonymous_variant |

| | | | | | |
|---|---|---|---|---|---|
| 4 | 114878685 | Stil | T | G | missense_variant |
| 4 | 114880792 | Stil | A | G | synonymous_variant |
| 4 | 114881514 | Stil | T | C | missense_variant |
| 4 | 114887126 | Stil | G | A | missense_variant |
| 4 | 114887151 | Stil | A | G | missense_variant |
| 4 | 114895571 | Stil | T | A | synonymous_variant |
| 4 | 114896341 | Stil | G | A | missense_variant |
| 4 | 117761602 | Ipo13 | C | T | synonymous_variant |
| 4 | 119953321 | Hivep3 | C | T | missense_variant |
| 4 | 123082829 | Hpcal4 | T | C | synonymous_variant |
| 4 | 123082862 | Hpcal4 | C | T | synonymous_variant |
| 4 | 123082994 | Hpcal4 | C | T | synonymous_variant |
| 4 | 123106084 | Nt5c1a | C | A | synonymous_variant |
| 4 | 123109742 | Nt5c1a | A | C | synonymous_variant |
| 4 | 123140011 | Heyl | A | G | missense_variant |
| 4 | 123191549 | Pabpc4 | G | A | synonymous_variant |
| 4 | 123215964 | Oxct2a | T | C | synonymous_variant |
| 4 | 124599677 | Fhl3 | G | A | missense_variant,splice_region_variant |
| 4 | 126060775 | Thrap3 | C | T | synonymous_variant |
| 4 | 126615851 | Tfap2e | G | A | synonymous_variant |
| 4 | 128605991 | Phc2 | G | A | missense_variant |
| 4 | 128642037 | Phc2 | G | A | stop_gained |
| 4 | 129117503 | C77080 | C | T | missense_variant |
| 4 | 132065537 | Rcc1 | G | A | missense_variant |
| 4 | 135706191 | Pithd1 | C | T | missense_variant |
| 4 | 135991845 | Tcea3 | G | - | frameshift_variant |
| 4 | 137296076 | Hspg2 | C | T | synonymous_variant |
| 4 | 138166806 | Mul1 | C | T | missense_variant |
| 4 | 141048529 | Epha2 | A | C | splice_region_variant,synonymous_variant |
| 4 | 144950989 | Tnfrsf1b | G | A | missense_variant |
| 4 | 154425331 | Prdm16 | C | T | missense_variant |
| 4 | 154613244 | Prdm16 | G | A | missense_variant |
| 4 | 155507044 | Cfap74 | G | C | missense_variant |
| 4 | 155857493 | Vwa1 | G | A | missense_variant |
| 5 | 4119068 | Akap9 | A | - | frameshift_variant |
| 5 | 4119075 | Akap9 | GAACA | - | frameshift_variant |
| 5 | 8735390 | Abcb1a | G | A | missense_variant |
| 5 | 20807400 | Magi2 | G | T | missense_variant |
| 5 | 22016306 | Slc26a5 | A | C | missense_variant |
| 5 | 31235018 | Cad | G | T | missense_variant |
| 5 | 31411999 | Ift172 | T | A | stop_gained,splice_region_variant |
| 5 | 44630677 | Ldb2 | G | A | missense_variant |
| 5 | 64421746 | Tbc1d1 | G | A | synonymous_variant |
| 5 | 73179974 | Fryl | G | A | missense_variant |
| 5 | 87611938 | Ugt2a1 | G | A | missense_variant |
| 5 | 92807628 | Ccdc158 | A | G | synonymous_variant |
| 5 | 93088033 | Shroom3 | G | A | missense_variant |
| 5 | 96227942 | Cnot6l | G | A | synonymous_variant |
| 5 | 96707764 | Fras1 | A | C | missense_variant |
| 5 | 96906001 | Fras1 | T | C | missense_variant |
| 5 | 100939735 | Helq | AC | - | frameshift_variant |
| 5 | 103137589 | Mapk10 | T | C | synonymous_variant |
| 5 | 107647913 | Btbd8 | G | A | missense_variant |
| 5 | 109445346 | Vmn2r15 | G | A | missense_variant |
| 5 | 110478828 | Pole | C | T | synonymous_variant |
| 5 | 110831107 | Ep400 | C | T | synonymous_variant |

| | | | | | |
|---|---|---|---|---|---|
| 5 | 112539154 | Asphd2 | G | A | synonymous_variant |
| 5 | 121486509 | Hectd4 | C | T | missense_variant |
| 5 | 124715091 | Eif2b1 | G | A | synonymous_variant |
| 5 | 124802257 | Dnah10 | C | T | synonymous_variant |
| 5 | 125106222 | Ncor2 | G | A | missense_variant |
| 5 | 127640333 | Tmem132c | C | G | missense_variant |
| 5 | 134739982 | Eln | C | A | missense_variant |
| 5 | 137359444 | Ephb4 | C | T | missense_variant |
| 5 | 137392781 | Zan | G | C | missense_variant |
| 5 | 137640261 | Sap25 | G | A | missense_variant |
| 5 | 137642920 | Irs3 | C | T | missense_variant |
| 5 | 137796160 | Zcwpw1 | G | C | missense_variant,splice_region_variant |
| 5 | 138280206 | Stag3 | C | T | stop_gained |
| 5 | 142031952 | Sdk1 | T | C | missense_variant |
| 5 | 144149391 | Tecpr1 | C | T | splice_region_variant,synonymous_variant |
| 6 | 3373048 | Samd9l | - | T | frameshift_variant |
| 6 | 8022295 | Col28a1 | C | T | synonymous_variant |
| 6 | 8079750 | Col28a1 | C | A | missense_variant |
| 6 | 8155400 | Col28a1 | T | G | missense_variant |
| 6 | 8175216 | Col28a1 | C | T | missense_variant |
| 6 | 8175266 | Col28a1 | A | G | missense_variant |
| 6 | 8175433 | Col28a1 | G | A | synonymous_variant |
| 6 | 13630676 | Bmt2 | T | C | synonymous_variant |
| 6 | 22961610 | Ptprz1 | C | T | missense_variant |
| 6 | 23077165 | Aass | G | A | synonymous_variant |
| 6 | 24454251 | Iqub | A | G | missense_variant |
| 6 | 24505603 | Iqub | C | T | missense_variant |
| 6 | 24734530 | Hyal6 | C | T | synonymous_variant |
| 6 | 28420287 | Gcc1 | C | T | synonymous_variant |
| 6 | 29169831 | Prrt4 | C | T | missense_variant |
| 6 | 29380113 | Opn1sw | C | T | missense_variant |
| 6 | 30585027 | Cpa4 | C | T | synonymous_variant |
| 6 | 30742758 | Mest | A | G | missense_variant |
| 6 | 38604604 | Klrg2 | C | T | missense_variant |
| 6 | 39377390 | Mkrn1 | T | A | missense_variant |
| 6 | 42652113 | Tcaf1 | C | T | missense_variant |
| 6 | 56068417 | Pde1c | C | T | splice_acceptor_variant |
| 6 | 65673091 | Ndnf | C | T | missense_variant |
| 6 | 73446139 | 31417E11R | T | C | missense_variant |
| 6 | 83126500 | Rtkn | G | A | missense_variant |
| 6 | 88888860 | Tpra1 | C | T | synonymous_variant |
| 6 | 106502432 | Cntn4 | C | T | synonymous_variant |
| 6 | 112292587 | Lmcd1 | C | T | synonymous_variant |
| 6 | 121310338 | Slc6a13 | C | G | missense_variant |
| 6 | 141416850 | Pde3a | T | C | missense_variant |
| 6 | 148700144 | Ipo8 | C | A | missense_variant |
| 6 | 148956478 | Dennd5b | G | C | missense_variant |
| 7 | 3618515 | Oscar | C | T | stop_gained,splice_region_variant |
| 7 | 5199131 | Vmn1r56 | C | T | missense_variant |
| 7 | 12039513 | Vmn1r82 | G | A | missense_variant |
| 7 | 16888727 | Ceacam3 | T | C | synonymous_variant |
| 7 | 18263703 | Psg25 | G | A | missense_variant |
| 7 | 19621704 | Ceacam19 | G | A | synonymous_variant |
| 7 | 26891031 | Snrpa | G | A | missense_variant,splice_region_variant |
| 7 | 29492534 | Zfp30 | C | T | synonymous_variant |
| 7 | 44541287 | Med25 | G | A | synonymous_variant |

| | | | | | |
|---|---|---|---|---|---|
| 7 | 44613340 | Cpt1c | C | T | synonymous_variant |
| 7 | 44959775 | Trpm4 | G | A | synonymous_variant |
| 7 | 45635914 | Abcc6 | G | A | synonymous_variant |
| 7 | 45715641 | Nomo1 | G | A | missense_variant |
| 7 | 48096888 | Mrgprb1 | T | A | synonymous_variant |
| 7 | 55781040 | Herc2 | G | A | missense_variant |
| 7 | 78761070 | Acan | T | C | synonymous_variant |
| 7 | 78761102 | Acan | A | C | missense_variant |
| 7 | 79335553 | Ticrr | C | T | synonymous_variant |
| 7 | 86421025 | Folh1 | C | T | synonymous_variant |
| 7 | 97066264 | Thrsp | A | G | synonymous_variant |
| 7 | 97145045 | Ints4 | C | G | missense_variant |
| 7 | 97184448 | Ints4 | C | T | missense_variant |
| 7 | 101435892 | Clpb | G | A | missense_variant |
| 7 | 102184288 | Olfr547 | C | T | synonymous_variant |
| 7 | 102328260 | Trim68 | G | A | missense_variant |
| 7 | 102363490 | Olfr33 | C | T | synonymous_variant |
| 7 | 103320128 | Olfr624 | C | A | stop_gained |
| 7 | 103461922 | Hbb-bt | C | T | missense_variant |
| 7 | 103542460 | Olfr64 | G | C | missense_variant |
| 7 | 104229582 | Olfr653 | C | T | missense_variant |
| 7 | 104489574 | Usp17lb | A | C | missense_variant |
| 7 | 106491294 | Olfr707 | G | A | synonymous_variant |
| 7 | 107567321 | Olfr476 | C | T | missense_variant |
| 7 | 108064578 | Olfr498 | T | A | missense_variant |
| 7 | 108940648 | Stk33 | C | T | missense_variant |
| 7 | 114105498 | Pde3b | C | T | synonymous_variant |
| 7 | 115779704 | Plekha7 | G | C | missense_variant |
| 7 | 122600840 | Rbbp6 | C | T | missense_variant |
| 7 | 122781531 | Tnrc6a | G | A | synonymous_variant |
| 7 | 126092473 | Atxn2l | C | T | missense_variant |
| 7 | 127074601 | Prr14 | C | T | missense_variant |
| 7 | 127525640 | Prss8 | A | G | missense_variant |
| 7 | 127843657 | Armc5 | G | T | missense_variant |
| 7 | 139584880 | Tubgcp2 | G | A | synonymous_variant |
| 7 | 139876257 | 30411N06R | G | A | synonymous_variant |
| 7 | 141226186 | Muc6 | - | T | frameshift_variant |
| 7 | 141363481 | Muc5ac | C | T | missense_variant |
| 7 | 142736890 | Kcnq1 | G | A | missense_variant |
| 7 | 143391549 | Dhcr7 | C | T | missense_variant |
| 8 | 4186825 | Cd209g | - | ATCGGCC | frameshift_variant |
| 8 | 4262998 | Prr36 | C | T | synonymous_variant |
| 8 | 10420902 | Myo16 | C | T | synonymous_variant |
| 8 | 10526154 | Myo16 | G | A | missense_variant |
| 8 | 12630944 | Spaca7 | G | A | missense_variant,splice_region_variant |
| 8 | 12691166 | Tubgcp3 | T | C | synonymous_variant |
| 8 | 12705953 | Tubgcp3 | C | T | missense_variant |
| 8 | 13059517 | Mcf2l | T | C | synonymous_variant |
| 8 | 13177770 | Cul4a | A | G | synonymous_variant |
| 8 | 13186361 | Cul4a | A | G | synonymous_variant |
| 8 | 13196624 | Cul4a | A | G | synonymous_variant |
| 8 | 13217916 | Lamp1 | G | A | synonymous_variant |
| 8 | 13446709 | Atp4b | G | A | synonymous_variant |
| 8 | 13463086 | Grk1 | C | T | synonymous_variant |
| 8 | 13466162 | Grk1 | C | T | synonymous_variant |
| 8 | 13636919 | Rasa3 | T | C | synonymous_variant |

| | | | | | |
|---|---|---|---|---|---|
| 8 | 13819392 | Cdc16 | G | A | synonymous_variant |
| 8 | 13887051 | AF366264 | C | T | missense_variant |
| 8 | 13928351 | Champ1 | T | A | missense_variant |
| 8 | 13928573 | Champ1 | C | A | missense_variant |
| 8 | 13929488 | Champ1 | T | C | missense_variant |
| 8 | 13929694 | Champ1 | G | A | synonymous_variant |
| 8 | 13930201 | Champ1 | T | C | synonymous_variant |
| 8 | 14146129 | Dlgap2 | T | C | synonymous_variant |
| 8 | 14777041 | Dlgap2 | G | A | synonymous_variant |
| 8 | 14879869 | Dlgap2 | G | A | missense_variant,splice_region_variant |
| 8 | 14984842 | Arhgef10 | G | A | missense_variant |
| 8 | 14995358 | Arhgef10 | G | C | missense_variant |
| 8 | 15011132 | Arhgef10 | C | T | synonymous_variant |
| 8 | 15028445 | Arhgef10 | T | C | synonymous_variant |
| 8 | 15029889 | Arhgef10 | A | G | synonymous_variant |
| 8 | 15041215 | Arhgef10 | T | C | synonymous_variant |
| 8 | 15049383 | Arhgef10 | G | T | synonymous_variant |
| 8 | 15049722 | Arhgef10 | A | G | synonymous_variant |
| 8 | 15077837 | Kbtbd11 | A | G | synonymous_variant |
| 8 | 15077888 | Kbtbd11 | G | C | synonymous_variant |
| 8 | 15078507 | Kbtbd11 | C | G | missense_variant |
| 8 | 15152506 | Myom2 | G | A | missense_variant |
| 8 | 15152625 | Myom2 | A | G | synonymous_variant |
| 8 | 15182764 | Myom2 | T | C | synonymous_variant |
| 8 | 16039077 | Csmd1 | T | C | missense_variant |
| 8 | 23717158 | Gins4 | C | G | missense_variant |
| 8 | 35936295 | Eri1 | T | A | missense_variant |
| 8 | 48848632 | Tenm3 | C | A | missense_variant |
| 8 | 53966609 | Aga | C | A | missense_variant |
| 8 | 53970806 | Aga | C | T | synonymous_variant |
| 8 | 53970858 | Aga | T | C | missense_variant |
| 8 | 53973302 | Aga | C | T | synonymous_variant |
| 8 | 54040248 | Neil3 | T | C | missense_variant |
| 8 | 54040354 | Neil3 | G | A | synonymous_variant |
| 8 | 54042056 | Neil3 | A | G | synonymous_variant |
| 8 | 54054054 | Neil3 | A | G | missense_variant |
| 8 | 54060410 | Neil3 | A | G | missense_variant |
| 8 | 54076665 | Neil3 | G | T | missense_variant |
| 8 | 54091810 | Neil3 | A | G | missense_variant |
| 8 | 55053925 | Spata4 | C | T | synonymous_variant |
| 8 | 55114284 | Wdr17 | C | T | synonymous_variant |
| 8 | 55118131 | Wdr17 | T | C | missense_variant |
| 8 | 55134381 | Wdr17 | C | T | splice_region_variant,synonymous_variant |
| 8 | 56324495 | Adam29 | T | C | missense_variant |
| 8 | 56324717 | Adam29 | A | T | missense_variant |
| 8 | 56325965 | Adam29 | C | G | missense_variant |
| 8 | 56444209 | Glra3 | A | T | synonymous_variant |
| 8 | 56444230 | Glra3 | G | A | synonymous_variant |
| 8 | 56751446 | Hpgd | T | C | synonymous_variant |
| 8 | 58290284 | Galntl6 | A | G | synonymous_variant |
| 8 | 58411350 | Galntl6 | C | G | synonymous_variant |
| 8 | 69254983 | Ints10 | G | A | missense_variant |
| 8 | 70905205 | Klhl26 | G | A | missense_variant |
| 8 | 71840113 | Ushbp1 | G | A | missense_variant |
| 8 | 73215879 | Cherp | TGGACC | - | inframe_deletion |
| 8 | 79052112 | Ttc29 | C | A | missense_variant |

| | | | | | |
|---|---|---|---|---|---|
| 8 | 81340047 | Frem3 | C | T | missense_variant |
| 8 | 84400318 | Pkn1 | T | A | missense_variant |
| 8 | 85276434 | Cacna1a | G | A | missense_variant |
| 8 | 89318314 | Nkd1 | C | A | missense_variant |
| 8 | 90975480 | Tox3 | G | A | missense_variant |
| 8 | 91660000 | Chd9 | C | T | missense_variant |
| 8 | 92525593 | Irx3 | T | C | missense_variant |
| 8 | 95119117 | Herpud1 | T | C | synonymous_variant |
| 8 | 95119138 | Herpud1 | T | C | synonymous_variant |
| 8 | 95119141 | Herpud1 | A | G | synonymous_variant |
| 8 | 95199639 | Nlrc5 | G | C | missense_variant |
| 8 | 95202618 | Nlrc5 | A | G | synonymous_variant |
| 8 | 95203366 | Nlrc5 | T | G | missense_variant |
| 8 | 95203370 | Nlrc5 | T | C | missense_variant |
| 8 | 95203634 | Nlrc5 | T | C | missense_variant |
| 8 | 95203728 | Nlrc5 | A | G | synonymous_variant |
| 8 | 95203794 | Nlrc5 | G | A | synonymous_variant |
| 8 | 95203839 | Nlrc5 | T | G | missense_variant |
| 8 | 95203899 | Nlrc5 | T | C | synonymous_variant |
| 8 | 95208658 | Nlrc5 | T | C | synonymous_variant |
| 8 | 95210708 | Nlrc5 | G | A | missense_variant |
| 8 | 95213312 | Nlrc5 | G | A | missense_variant |
| 8 | 95215219 | Nlrc5 | A | G | missense_variant |
| 8 | 95218172 | Nlrc5 | G | A | synonymous_variant |
| 8 | 95219672 | Nlrc5 | T | C | synonymous_variant |
| 8 | 95247469 | Nlrc5 | C | T | synonymous_variant |
| 8 | 95281575 | Cpne2 | G | C | synonymous_variant |
| 8 | 95399716 | Arl2bp | G | A | synonymous_variant |
| 8 | 95403926 | Pllp | G | A | synonymous_variant |
| 8 | 95538478 | Ccl17 | A | G | synonymous_variant |
| 8 | 95571528 | Coq9 | T | C | synonymous_variant |
| 8 | 95576815 | Coq9 | G | T | synonymous_variant |
| 8 | 95579775 | Coq9 | C | T | synonymous_variant |
| 8 | 95579838 | Coq9 | G | T | synonymous_variant |
| 8 | 95668355 | Adgrg5 | C | T | synonymous_variant |
| 8 | 95668691 | Adgrg5 | A | G | synonymous_variant |
| 8 | 95668721 | Adgrg5 | G | A | synonymous_variant |
| 8 | 95763517 | Adgrg3 | G | A | missense_variant |
| 8 | 95861396 | Kifc3 | G | A | synonymous_variant |
| 8 | 95994371 | Cngb1 | A | G | synonymous_variant |
| 8 | 96023160 | Cngb1 | G | A | synonymous_variant |
| 8 | 96023278 | Cngb1 | A | G | missense_variant |
| 8 | 96024447 | Cngb1 | G | T | missense_variant |
| 8 | 96030324 | Cngb1 | T | C | missense_variant |
| 8 | 96030342 | Cngb1 | G | A | missense_variant |
| 8 | 96039626 | Tepp | C | G | synonymous_variant |
| 8 | 96039638 | Tepp | T | C | synonymous_variant |
| 8 | 96070594 | Usb1 | C | T | synonymous_variant |
| 8 | 96071932 | Usb1 | G | A | missense_variant |
| 8 | 96071952 | Usb1 | A | G | synonymous_variant |
| 8 | 96098974 | Mmp15 | C | T | synonymous_variant |
| 8 | 96614914 | Got2 | C | T | missense_variant |
| 8 | 103361086 | Cdh11 | C | T | missense_variant |
| 8 | 103377259 | Cdh11 | T | A | missense_variant |
| 8 | 103406295 | Cdh11 | T | C | synonymous_variant |
| 8 | 106108676 | Plekhg4 | G | A | synonymous_variant |

| | | | | | |
|---|---|---|---|---|---|
| 8 | 106111667 | Kctd19 | G | A | synonymous_variant |
| 8 | 106257584 | Atp6v0d1 | C | T | missense_variant |
| 8 | 106575419 | Cenpt | C | T | missense_variant |
| 8 | 107375652 | Cdh1 | T | C | synonymous_variant |
| 8 | 107380258 | Cdh1 | C | T | synonymous_variant |
| 8 | 107380264 | Cdh1 | T | C | synonymous_variant |
| 8 | 107383561 | Cdh1 | CC | GA | missense_variant |
| 8 | 107383577 | Cdh1 | T | C | missense_variant |
| 8 | 107384870 | Cdh1 | T | C | synonymous_variant |
| 8 | 107384945 | Cdh1 | A | G | synonymous_variant |
| 8 | 107386359 | Cdh1 | T | C | synonymous_variant |
| 8 | 107395235 | Cdh1 | T | C | synonymous_variant |
| 8 | 107409833 | Tango6 | A | G | missense_variant |
| 8 | 107468805 | Tango6 | CCAGAGT | - | inframe_deletion |
| 8 | 107508339 | Tango6 | T | C | synonymous_variant |
| 8 | 107545156 | Tango6 | C | T | synonymous_variant |
| 8 | 107545159 | Tango6 | A | G | synonymous_variant |
| 8 | 107545198 | Tango6 | A | G | synonymous_variant |
| 8 | 107600792 | Has3 | C | T | missense_variant |
| 8 | 107612177 | Derpc | T | C | missense_variant |
| 8 | 107771673 | Vps4a | T | G | splice_region_variant,synonymous_variant |
| 8 | 107774897 | Pdf | C | A | missense_variant |
| 8 | 107775529 | Cog8 | T | C | missense_variant |
| 8 | 107775625 | Cog8 | G | T | missense_variant |
| 8 | 107788226 | Tmed6 | - | TAGC | frameshift_variant,stop_retained_variant |
| 8 | 107788280 | Tmed6 | G | A | synonymous_variant |
| 8 | 107788301 | Tmed6 | G | A | synonymous_variant |
| 8 | 107788388 | Tmed6 | T | C | synonymous_variant |
| 8 | 107790390 | Tmed6 | G | A | synonymous_variant |
| 8 | 107803306 | Terf2 | T | C | missense_variant |
| 8 | 108139671 | Nob1 | G | A | missense_variant |
| 8 | 109673739 | Zfhx3 | G | A | synonymous_variant |
| 8 | 110226036 | Pmfbp1 | T | C | synonymous_variant |
| 8 | 110756697 | Chst4 | A | G | missense_variant |
| 8 | 110756952 | Chst4 | T | C | missense_variant |
| 8 | 110757046 | Chst4 | T | C | synonymous_variant |
| 8 | 111053040 | Hydin | A | G | synonymous_variant |
| 8 | 111061417 | Hydin | C | T | synonymous_variant |
| 8 | 111137289 | Hydin | C | T | synonymous_variant |
| 8 | 111139922 | Hydin | G | T | synonymous_variant |
| 8 | 111769424 | Aars | G | A | missense_variant |
| 8 | 112440516 | Bcar1 | C | G | missense_variant |
| 8 | 113478983 | Cntnap4 | G | T | missense_variant |
| 8 | 114436349 | Adamts18 | G | C | missense_variant |
| 8 | 114502026 | Adamts18 | G | T | synonymous_variant |
| 8 | 114571607 | Adamts18 | T | C | synonymous_variant |
| 8 | 114998123 | Vat1l | G | T | synonymous_variant |
| 8 | 115433001 | Wwox | G | A | synonymous_variant |
| 8 | 117234276 | Dynlrb2 | T | C | synonymous_variant |
| 8 | 117234279 | Dynlrb2 | C | T | synonymous_variant |
| 8 | 117234285 | Dynlrb2 | G | A | synonymous_variant |
| 8 | 117234291 | Dynlrb2 | C | T | synonymous_variant |
| 8 | 117234312 | Dynlrb2 | A | G | synonymous_variant |
| 8 | 117661484 | Cenpn | G | A | synonymous_variant |
| 8 | 117683250 | Atmin | A | G | synonymous_variant |
| 8 | 117683295 | Atmin | G | A | synonymous_variant |

| | | | | | |
|---|---|---|---|---|---|
| 8 | 117684001 | Atmin | A | G | missense_variant |
| 8 | 117684034 | Atmin | A | G | missense_variant |
| 8 | 117684183 | Atmin | A | G | synonymous_variant |
| 8 | 117684291 | Atmin | G | A | synonymous_variant |
| 8 | 117697882 | '00030J22R | T | G | synonymous_variant |
| 8 | 117698254 | '00030J22R | G | A | synonymous_variant |
| 8 | 117700326 | '00030J22R | C | G | synonymous_variant |
| 8 | 117726722 | Pkd1l2 | C | A | missense_variant,splice_region_variant |
| 8 | 117755132 | Pkd1l2 | T | C | missense_variant |
| 8 | 117755188 | Pkd1l2 | T | G | missense_variant |
| 8 | 117756313 | Pkd1l2 | G | A | missense_variant |
| 8 | 117756320 | Pkd1l2 | G | A | synonymous_variant |
| 8 | 117757399 | Pkd1l2 | A | G | synonymous_variant |
| 8 | 117772885 | Pkd1l2 | A | C | missense_variant |
| 8 | 117774136 | Pkd1l2 | C | T | missense_variant |
| 8 | 117774140 | Pkd1l2 | G | A | missense_variant |
| 8 | 117774163 | Pkd1l2 | C | G | synonymous_variant |
| 8 | 117774208 | Pkd1l2 | A | G | synonymous_variant |
| 8 | 117774288 | Pkd1l2 | A | G | missense_variant |
| 8 | 117776653 | Pkd1l2 | G | A | missense_variant |
| 8 | 117776676 | Pkd1l2 | C | G | missense_variant |
| 8 | 117776710 | Pkd1l2 | T | C | missense_variant |
| 8 | 117776746 | Pkd1l2 | A | G | missense_variant |
| 8 | 117777796 | Pkd1l2 | C | T | synonymous_variant |
| 8 | 117777820 | Pkd1l2 | C | T | synonymous_variant |
| 8 | 117777855 | Pkd1l2 | G | T | missense_variant |
| 8 | 117778407 | Pkd1l2 | A | C | missense_variant |
| 8 | 117778433 | Pkd1l2 | G | A | synonymous_variant |
| 8 | 117778514 | Pkd1l2 | G | A | synonymous_variant |
| 8 | 117778524 | Pkd1l2 | A | G | missense_variant |
| 8 | 117781643 | Pkd1l2 | C | A | missense_variant |
| 8 | 117784162 | Pkd1l2 | A | G | missense_variant |
| 8 | 117784242 | Pkd1l2 | GG | AA | missense_variant |
| 8 | 117784256 | Pkd1l2 | G | A | missense_variant |
| 8 | 117792444 | Pkd1l2 | T | C | synonymous_variant |
| 8 | 117802867 | Pkd1l2 | T | C | synonymous_variant |
| 8 | 117802879 | Pkd1l2 | C | T | synonymous_variant |
| 8 | 117802897 | Pkd1l2 | AC | GT | missense_variant |
| 8 | 117802939 | Pkd1l2 | C | T | synonymous_variant |
| 8 | 117807483 | Pkd1l2 | T | C | missense_variant |
| 8 | 117807548 | Pkd1l2 | T | G | synonymous_variant |
| 8 | 117807569 | Pkd1l2 | A | G | synonymous_variant |
| 8 | 117808075 | Pkd1l2 | C | T | missense_variant |
| 8 | 117808149 | Pkd1l2 | C | G | missense_variant |
| 8 | 117808168 | Pkd1l2 | C | T | missense_variant |
| 8 | 117809084 | Pkd1l2 | A | G | synonymous_variant |
| 8 | 117822798 | Bco1 | A | T | missense_variant |
| 8 | 117832454 | Bco1 | T | C | synonymous_variant |
| 8 | 117840312 | Bco1 | G | A | missense_variant |
| 8 | 117854246 | Bco1 | C | T | synonymous_variant |
| 8 | 117854258 | Bco1 | T | C | synonymous_variant |
| 8 | 117917460 | Gan | G | A | synonymous_variant |
| 8 | 119963693 | Cdh13 | C | T | synonymous_variant |
| 8 | 120901510 | Cibar2 | C | T | missense_variant |
| 8 | 123128424 | Zc3h18 | G | T | missense_variant |
| 8 | 123957579 | Cdk10 | C | T | missense_variant |

| | | | | | |
|---|---|---|---|---|---|
| 9 | 7102439 | Dync2h1 | C | T | missense_variant |
| 9 | 9673349 | Cntn5 | C | T | missense_variant |
| 9 | 14662891 | Fut4 | C | T | synonymous_variant |
| 9 | 15640021 | Slc36a4 | C | T | missense_variant |
| 9 | 16289551 | Fat3 | C | A | splice_acceptor_variant |
| 9 | 21102786 | Pde4a | T | C | missense_variant |
| 9 | 21143029 | Keap1 | T | C | missense_variant |
| 9 | 21156364 | S1pr5 | A | C | missense_variant |
| 9 | 45827380 | Pcsk7 | G | A | synonymous_variant |
| 9 | 54922990 | Chrna3 | T | C | missense_variant |
| 9 | 56166689 | Peak1 | G | A | missense_variant |
| 9 | 56167712 | Peak1 | G | A | missense_variant |
| 9 | 58897565 | Neo1 | G | A | synonymous_variant |
| 9 | 63431993 | Iqch | G | A | synonymous_variant |
| 9 | 65187413 | Cilp | G | - | frameshift_variant |
| 9 | 66939779 | Tpm1 | G | A | missense_variant |
| 9 | 69281370 | Rora | G | A | synonymous_variant |
| 9 | 78071848 | Cilk1 | G | T | missense_variant |
| 9 | 79681408 | Tmem30a | G | A | missense_variant |
| 9 | 80021197 | Senp6 | A | G | synonymous_variant |
| 9 | 95552177 | Pcolce2 | G | A | synonymous_variant |
| 9 | 95818404 | Atr | C | T | missense_variant |
| 9 | 96706775 | Pxylp1 | G | A | missense_variant |
| 9 | 99523892 | Dzip1l | A | T | missense_variant |
| 9 | 100434899 | Slc35g2 | C | T | synonymous_variant |
| 9 | 100435458 | Slc35g2 | G | A | missense_variant |
| 9 | 103330319 | Bfsp2 | C | T | missense_variant |
| 9 | 104750042 | Cpne4 | G | A | synonymous_variant |
| 9 | 108440646 | Impdh2 | C | A | missense_variant |
| 9 | 108942752 | Plxnb1 | G | A | synonymous_variant |
| 9 | 108974605 | Fbxw21 | T | C | missense_variant |
| 9 | 110325984 | Kif9 | G | A | splice_acceptor_variant |
| 9 | 110851378 | Ltf | G | A | missense_variant |
| 9 | 115835692 | Gadl1 | G | A | synonymous_variant |
| 9 | 118895017 | Vill | G | A | synonymous_variant |
| 9 | 120779675 | Ctnnb1 | G | A | missense_variant |
| 9 | 120986235 | Ulk4 | T | C | synonymous_variant |
| 9 | 120986247 | Ulk4 | G | A | synonymous_variant |
| 9 | 120997345 | Ulk4 | A | G | synonymous_variant |
| 9 | 121037235 | Ulk4 | A | G | synonymous_variant |
| 9 | 121073447 | Ulk4 | A | G | synonymous_variant |
| 9 | 121073498 | Ulk4 | A | C | synonymous_variant |
| 9 | 121080060 | Ulk4 | C | T | synonymous_variant |
| 9 | 121080072 | Ulk4 | A | G | synonymous_variant |
| 9 | 121081729 | Ulk4 | T | C | synonymous_variant |
| 9 | 121086388 | Ulk4 | C | T | synonymous_variant |
| 9 | 121086458 | Ulk4 | G | A | missense_variant |
| 9 | 121091685 | Ulk4 | C | T | missense_variant |
| 9 | 121487180 | Vipr1 | G | A | synonymous_variant |
| 9 | 121493628 | Vipr1 | C | T | synonymous_variant |
| 9 | 121497539 | Vipr1 | T | C | synonymous_variant |
| 9 | 121497605 | Vipr1 | T | C | synonymous_variant |
| 9 | 121498511 | Vipr1 | A | C | synonymous_variant |
| 9 | 121498553 | Vipr1 | A | G | synonymous_variant |
| 9 | 121513889 | Sec22c | C | A | synonymous_variant |
| 9 | 121519376 | Sec22c | G | T | missense_variant |

| | | | | | |
|---|---|---|---|---|---|
| 9 | 121524755 | Sec22c | G | A | synonymous_variant |
| 9 | 121571752 | Nktr | A | G | synonymous_variant |
| 9 | 121571779 | Nktr | A | G | synonymous_variant |
| 9 | 121606973 | Klhl40 | C | T | synonymous_variant |
| 9 | 121678733 | Higd1a | C | T | synonymous_variant |
| 9 | 121744221 | Cyp8b1 | T | C | synonymous_variant |
| 9 | 121744320 | Cyp8b1 | T | G | synonymous_variant |
| 9 | 121744607 | Cyp8b1 | T | C | missense_variant |
| 9 | 121744623 | Cyp8b1 | A | G | synonymous_variant |
| 9 | 121744977 | Cyp8b1 | A | G | synonymous_variant |
| 9 | 121995957 | Snrk | G | C | missense_variant |
| 9 | 122681582 | Zfp445 | C | T | synonymous_variant |
| 9 | 122717677 | Zkscan7 | A | T | missense_variant |
| 9 | 122717876 | Zkscan7 | A | G | synonymous_variant |
| 9 | 122717983 | Zkscan7 | A | G | missense_variant |
| 9 | 122723962 | Zkscan7 | G | A | synonymous_variant |
| 9 | 122724019 | Zkscan7 | G | A | synonymous_variant |
| 9 | 122754288 | Zfp105 | A | C | missense_variant |
| 9 | 122758872 | Zfp105 | G | T | missense_variant |
| 9 | 122789039 | Kif15 | G | A | missense_variant |
| 9 | 122815283 | Kif15 | A | G | synonymous_variant |
| 9 | 122815629 | Kif15 | T | G | missense_variant |
| 9 | 122821037 | Kif15 | G | A | synonymous_variant |
| 9 | 122823083 | Kif15 | C | T | missense_variant |
| 9 | 122825436 | Kif15 | G | C | synonymous_variant |
| 9 | 122828342 | Kif15 | T | C | synonymous_variant |
| 9 | 122890718 | Tgm4 | C | A | missense_variant |
| 9 | 122890887 | Tgm4 | C | T | synonymous_variant |
| 9 | 122891554 | Tgm4 | A | G | synonymous_variant |
| 9 | 122980167 | Clec3b | T | C | missense_variant |
| 9 | 123002610 | Cdcp1 | G | A | synonymous_variant |
| 9 | 123009270 | Cdcp1 | C | T | synonymous_variant |
| 9 | 123014233 | Cdcp1 | A | G | synonymous_variant |
| 9 | 123014302 | Cdcp1 | G | A | synonymous_variant |
| 9 | 123014312 | Cdcp1 | C | G | missense_variant |
| 9 | 123014353 | Cdcp1 | C | T | synonymous_variant |
| 9 | 123019178 | Cdcp1 | G | A | synonymous_variant |
| 9 | 123200986 | Lars2 | A | C | synonymous_variant |
| 9 | 123201001 | Lars2 | A | G | missense_variant |
| 9 | 123221892 | Lars2 | A | G | synonymous_variant |
| 9 | 123260964 | Lars2 | C | T | synonymous_variant |
| 9 | 123265381 | Lars2 | T | C | missense_variant |
| 9 | 123265388 | Lars2 | C | G | synonymous_variant |
| 9 | 123290621 | Lars2 | G | A | missense_variant |
| 9 | 123461230 | Slc6a20b | A | G | synonymous_variant |
| 9 | 123466175 | Slc6a20a | C | T | synonymous_variant |
| 10 | 19588395 | Il20ra | G | A | missense_variant |
| 10 | 28450895 | Ptprk | T | C | synonymous_variant |
| 10 | 40810599 | Wasf1 | G | A | synonymous_variant |
| 10 | 43873307 | Crybg1 | C | T | missense_variant |
| 10 | 55927495 | Tbc1d32 | T | G | missense_variant |
| 10 | 60129152 | Psap | G | A | synonymous_variant |
| 10 | 60130375 | Psap | C | T | synonymous_variant |
| 10 | 67073953 | Jmjd1c | T | C | missense_variant |
| 10 | 70118205 | Slc16a9 | G | A | missense_variant |
| 10 | 75475676 | Susd2 | G | A | missense_variant |

| | | | | | |
|---|---|---|---|---|---|
| 10 | 76211022 | Pcnt | G | A | missense_variant |
| 10 | 76895759 | Col18a1 | G | T | synonymous_variant |
| 10 | 77890861 | Dnmt3l | G | A | synonymous_variant |
| 10 | 80980143 | Zbtb7a | G | A | synonymous_variant |
| 10 | 86572795 | Ttc41 | G | A | synonymous_variant |
| 10 | 115015567 | Tph2 | T | C | synonymous_variant |
| 10 | 115015575 | Tph2 | T | G | synonymous_variant |
| 10 | 115018721 | Tph2 | C | T | synonymous_variant |
| 10 | 115038456 | Tbc1d15 | T | C | missense_variant |
| 10 | 115046167 | Tbc1d15 | T | C | synonymous_variant |
| 10 | 115293754 | Lgr5 | A | G | synonymous_variant |
| 10 | 115298654 | Lgr5 | T | C | synonymous_variant |
| 10 | 115302273 | Lgr5 | C | T | synonymous_variant |
| 10 | 115356527 | Lgr5 | A | G | missense_variant |
| 10 | 115663779 | Tspan8 | T | C | synonymous_variant |
| 10 | 115669131 | Tspan8 | T | C | synonymous_variant |
| 10 | 115669173 | Tspan8 | A | G | synonymous_variant |
| 10 | 115680031 | Tspan8 | C | T | synonymous_variant |
| 10 | 116062088 | Ptprr | G | A | synonymous_variant |
| 10 | 116829123 | Best3 | G | A | synonymous_variant |
| 10 | 116829129 | Best3 | CC | AT | synonymous_variant |
| 10 | 116829138 | Best3 | C | T | synonymous_variant |
| 10 | 116829147 | Best3 | G | C | synonymous_variant |
| 10 | 117608999 | Nup107 | T | C | synonymous_variant |
| 10 | 117620969 | Nup107 | G | T | missense_variant |
| 10 | 117978869 | Mdm1 | G | A | missense_variant,splice_region_variant |
| 10 | 117993171 | Mdm1 | T | C | synonymous_variant |
| 10 | 117994335 | Mdm1 | C | T | missense_variant |
| 10 | 117994437 | Mdm1 | A | G | missense_variant |
| 10 | 117995355 | Mdm1 | G | A | synonymous_variant |
| 10 | 118000189 | Mdm1 | C | T | synonymous_variant |
| 10 | 118000327 | Mdm1 | G | T | synonymous_variant |
| 10 | 118002540 | Mdm1 | C | A | synonymous_variant |
| 10 | 118130046 | Iltifb | C | T | synonymous_variant |
| 10 | 127021159 | Arhgef25 | T | G | missense_variant |
| 10 | 127878879 | Naca | C | T | missense_variant |
| 10 | 129517724 | Olfr802 | C | T | missense_variant |
| 11 | 3087016 | Sfi1 | T | A | missense_variant |
| 11 | 3454714 | Inpp5j | G | A | missense_variant |
| 11 | 3985147 | Sec14l4 | C | T | synonymous_variant |
| 11 | 4734489 | Nf2 | A | G | synonymous_variant |
| 11 | 5151945 | Kremen1 | C | T | missense_variant |
| 11 | 6063346 | Nudcd3 | T | C | missense_variant |
| 11 | 6143212 | Nudcd3 | C | T | missense_variant |
| 11 | 7158429 | Igfbp3 | G | A | missense_variant |
| 11 | 40570918 | Mat2b | G | A | missense_variant |
| 11 | 40570963 | Mat2b | G | T | missense_variant |
| 11 | 40573317 | Mat2b | G | T | synonymous_variant |
| 11 | 40576130 | Mat2b | A | T | synonymous_variant |
| 11 | 40594616 | Hmmr | T | C | synonymous_variant |
| 11 | 40594685 | Hmmr | A | C | synonymous_variant |
| 11 | 40594694 | Hmmr | T | C | synonymous_variant |
| 11 | 40596782 | Hmmr | C | T | synonymous_variant |
| 11 | 40596866 | Hmmr | T | C | synonymous_variant |
| 11 | 40598415 | Hmmr | C | T | synonymous_variant |
| 11 | 40600767 | Hmmr | C | T | synonymous_variant |

| | | | | | |
|---|---|---|---|---|---|
| 11 | 40604829 | Hmmr | G | T | missense_variant |
| 11 | 40604837 | Hmmr | T | C | synonymous_variant |
| 11 | 40604951 | Hmmr | G | A | synonymous_variant |
| 11 | 40605625 | Hmmr | C | T | synonymous_variant |
| 11 | 40606084 | Hmmr | T | G | synonymous_variant |
| 11 | 40614230 | Hmmr | C | A | missense_variant |
| 11 | 40614243 | Hmmr | G | C | missense_variant |
| 11 | 40614327 | Hmmr | G | T | synonymous_variant |
| 11 | 40614406 | Hmmr | G | A | missense_variant |
| 11 | 40614408 | Hmmr | G | A | synonymous_variant |
| 11 | 40642136 | Ccng1 | T | C | synonymous_variant |
| 11 | 40644871 | Ccng1 | A | C | missense_variant |
| 11 | 41811349 | Gabrg2 | G | A | synonymous_variant |
| 11 | 41858437 | Gabrg2 | G | A | synonymous_variant |
| 11 | 41862612 | Gabrg2 | A | G | synonymous_variant |
| 11 | 43042552 | Atp10b | A | G | synonymous_variant |
| 11 | 43044132 | Atp10b | A | G | missense_variant |
| 11 | 43150418 | Atp10b | C | T | missense_variant |
| 11 | 43313733 | Pttg1 | A | G | synonymous_variant |
| 11 | 43313772 | Pttg1 | A | C | synonymous_variant |
| 11 | 43313799 | Pttg1 | A | G | synonymous_variant |
| 11 | 43315588 | Pttg1 | G | A | synonymous_variant |
| 11 | 43316452 | Pttg1 | G | T | missense_variant |
| 11 | 43329094 | Slu7 | C | T | synonymous_variant |
| 11 | 43331446 | Slu7 | G | A | synonymous_variant |
| 11 | 43332302 | Slu7 | C | T | synonymous_variant |
| 11 | 43332733 | Slu7 | G | A | synonymous_variant |
| 11 | 43334140 | Slu7 | C | T | synonymous_variant |
| 11 | 43336022 | Slu7 | G | A | synonymous_variant |
| 11 | 43336036 | Slu7 | G | A | missense_variant |
| 11 | 43336045 | Slu7 | C | G | missense_variant |
| 11 | 43336071 | Slu7 | G | A | missense_variant |
| 11 | 43336077 | Slu7 | C | A | missense_variant |
| 11 | 43336188 | Slu7 | A | G | splice_region_variant,synonymous_variant |
| 11 | 43336985 | Slu7 | C | G | synonymous_variant |
| 11 | 44300991 | Il12b | T | C | missense_variant |
| 11 | 44303354 | Il12b | T | C | missense_variant |
| 11 | 44303440 | Il12b | A | G | synonymous_variant |
| 11 | 44354551 | Ublcp1 | G | A | synonymous_variant |
| 11 | 44356835 | Ublcp1 | T | A | synonymous_variant |
| 11 | 44452399 | Rnf145 | T | C | synonymous_variant |
| 11 | 44452633 | Rnf145 | C | T | synonymous_variant |
| 11 | 44511367 | Ebf1 | G | T | synonymous_variant |
| 11 | 45793057 | Clint1 | T | C | synonymous_variant |
| 11 | 45797257 | Clint1 | A | G | missense_variant |
| 11 | 45882701 | Sox30 | C | T | missense_variant |
| 11 | 46227305 | Itk | G | A | synonymous_variant |
| 11 | 46227359 | Itk | A | G | synonymous_variant |
| 11 | 46233269 | Itk | T | C | synonymous_variant |
| 11 | 46246626 | Itk | T | C | synonymous_variant |
| 11 | 46298041 | Fam71b | A | G | synonymous_variant |
| 11 | 46347094 | Havcr2 | A | G | missense_variant |
| 11 | 46347098 | Havcr2 | C | G | missense_variant |
| 11 | 46347103 | Havcr2 | GT | AA | missense_variant |
| 11 | 46347106 | Havcr2 | T | G | missense_variant |
| 11 | 46347120 | Havcr2 | G | A | synonymous_variant |

| | | | | | |
|---|---|---|---|---|---|
| 11 | 46347151 | Havcr2 | T | C | missense_variant |
| 11 | 46347157 | Havcr2 | C | T | missense_variant |
| 11 | 46347163 | Havcr2 | G | A | missense_variant |
| 11 | 46347219 | Havcr2 | C | T | synonymous_variant |
| 11 | 46347303 | Havcr2 | C | T | synonymous_variant |
| 11 | 46419243 | Gm12169 | C | G | missense_variant |
| 11 | 46419418 | Gm12169 | A | G | missense_variant |
| 11 | 46561740 | Timd2 | G | A | synonymous_variant |
| 11 | 46570487 | Timd2 | GA | TC | missense_variant |
| 11 | 46646977 | Havcr1 | G | C | missense_variant |
| 11 | 48986281 | Ifi47 | G | A | missense_variant |
| 11 | 48986416 | Ifi47 | T | G | missense_variant |
| 11 | 48986664 | Ifi47 | G | A | missense_variant |
| 11 | 48986879 | Ifi47 | A | T | synonymous_variant |
| 11 | 48986925 | Ifi47 | T | C | missense_variant |
| 11 | 48986954 | Ifi47 | G | A | synonymous_variant |
| 11 | 48986958 | Ifi47 | GC | AT | missense_variant |
| 11 | 48986966 | Ifi47 | T | C | synonymous_variant |
| 11 | 48987050 | Ifi47 | G | T | synonymous_variant |
| 11 | 48987095 | Ifi47 | C | T | synonymous_variant |
| 11 | 48987332 | Ifi47 | G | A | synonymous_variant |
| 11 | 49003877 | Olfr1396 | G | A | synonymous_variant |
| 11 | 49003990 | Olfr1396 | A | G | synonymous_variant |
| 11 | 49025927 | Olfr56 | A | C | synonymous_variant |
| 11 | 49039089 | Olfr1395 | G | A | missense_variant |
| 11 | 49039556 | Olfr1395 | G | A | synonymous_variant |
| 11 | 49039862 | Olfr1395 | A | G | synonymous_variant |
| 11 | 49039982 | Olfr1395 | C | G | synonymous_variant |
| 11 | 49039993 | Olfr1395 | G | A | missense_variant |
| 11 | 49051296 | Olfr1394 | A | G | missense_variant |
| 11 | 49051370 | Olfr1394 | G | A | synonymous_variant |
| 11 | 49051412 | Olfr1394 | C | T | synonymous_variant |
| 11 | 49051646 | Olfr1394 | A | G | synonymous_variant |
| 11 | 49060250 | Btnl9 | A | G | synonymous_variant |
| 11 | 49060280 | Btnl9 | G | C | synonymous_variant |
| 11 | 49065679 | Btnl9 | T | C | missense_variant |
| 11 | 49066441 | Btnl9 | G | A | synonymous_variant |
| 11 | 49066448 | Btnl9 | A | G | missense_variant |
| 11 | 49069546 | Btnl9 | A | G | synonymous_variant |
| 11 | 49069618 | Btnl9 | T | C | synonymous_variant |
| 11 | 49069714 | Btnl9 | A | G | synonymous_variant |
| 11 | 49071410 | Btnl9 | C | G | missense_variant |
| 11 | 49071455 | Btnl9 | CT | GG | missense_variant |
| 11 | 49071701 | Btnl9 | A | G | synonymous_variant |
| 11 | 49106278 | Zfp62 | T | C | missense_variant |
| 11 | 49171001 | Olfr1393 | G | A | missense_variant |
| 11 | 49171584 | Olfr1393 | T | C | missense_variant |
| 11 | 49171858 | Olfr1393 | G | T | missense_variant |
| 11 | 49185031 | Olfr1392 | A | T | missense_variant |
| 11 | 49321676 | Olfr1389 | G | T | synonymous_variant |
| 11 | 49334767 | Olfr1388 | G | A | missense_variant |
| 11 | 49335173 | Olfr1388 | T | C | missense_variant |
| 11 | 49335335 | Olfr1388 | A | G | missense_variant |
| 11 | 49350807 | Olfr1387 | G | A | synonymous_variant |
| 11 | 49405405 | Olfr1384 | A | G | synonymous_variant |
| 11 | 49535203 | Flt4 | C | T | synonymous_variant |

| | | | | | |
|---|---|---|---|---|---|
| 11 | 50026751 | Tbc1d9b | T | C | missense_variant |
| 11 | 50043455 | Tbc1d9b | C | G | synonymous_variant |
| 11 | 50050493 | Tbc1d9b | C | A | synonymous_variant |
| 11 | 50187928 | Canx | A | C | missense_variant |
| 11 | 50288492 | Rufy1 | C | G | missense_variant |
| 11 | 50647552 | Adamts2 | G | A | synonymous_variant |
| 11 | 50647576 | Adamts2 | G | A | synonymous_variant |
| 11 | 50672700 | Adamts2 | C | T | synonymous_variant |
| 11 | 50676407 | Adamts2 | T | C | synonymous_variant |
| 11 | 50676488 | Adamts2 | G | C | synonymous_variant |
| 11 | 50678113 | Adamts2 | A | G | synonymous_variant |
| 11 | 50678140 | Adamts2 | G | A | synonymous_variant |
| 11 | 50679580 | Adamts2 | T | C | synonymous_variant |
| 11 | 50682682 | Adamts2 | A | G | synonymous_variant |
| 11 | 50694529 | Adamts2 | C | T | synonymous_variant |
| 11 | 50694602 | Adamts2 | G | C | missense_variant |
| 11 | 50694743 | Adamts2 | CCGTCT | - | inframe_deletion |
| 11 | 50708643 | Zfp354c | G | A | synonymous_variant |
| 11 | 50708709 | Zfp354c | C | T | synonymous_variant |
| 11 | 50742076 | Grm6 | C | A | synonymous_variant |
| 11 | 50742223 | Grm6 | A | G | synonymous_variant |
| 11 | 50744148 | Grm6 | A | C | synonymous_variant |
| 11 | 50744190 | Grm6 | T | C | synonymous_variant |
| 11 | 50746559 | Grm6 | G | A | synonymous_variant |
| 11 | 50747845 | Grm6 | T | C | synonymous_variant |
| 11 | 50750854 | Grm6 | T | C | synonymous_variant |
| 11 | 50750881 | Grm6 | G | A | synonymous_variant |
| 11 | 50814362 | Zfp354b | C | T | missense_variant |
| 11 | 50841781 | Prop1 | - | GGT | inframe_insertion |
| 11 | 50841894 | Prop1 | A | C | missense_variant |
| 11 | 50841941 | Prop1 | C | G | missense_variant |
| 11 | 50860481 | Olfr1378 | C | T | missense_variant |
| 11 | 50860766 | Olfr1378 | G | A | missense_variant |
| 11 | 50876066 | Olfr1377 | C | T | synonymous_variant |
| 11 | 50876216 | Olfr1377 | A | C | synonymous_variant |
| 11 | 50897897 | Olfr51 | T | C | missense_variant |
| 11 | 50898022 | Olfr51 | A | G | synonymous_variant |
| 11 | 50898293 | Olfr51 | C | T | stop_gained |
| 11 | 50898561 | Olfr51 | C | T | missense_variant |
| 11 | 50939232 | Olfr1375 | TCT | CTG | missense_variant |
| 11 | 50939311 | Olfr1375 | G | C | missense_variant |
| 11 | 50939613 | Olfr1375 | G | A | synonymous_variant |
| 11 | 50939796 | Olfr1375 | T | - | frameshift_variant |
| 11 | 50951692 | Zfp354a | GT | CG | missense_variant |
| 11 | 50960205 | Zfp354a | G | A | missense_variant |
| 11 | 50960237 | Zfp354a | A | G | synonymous_variant |
| 11 | 50960241 | Zfp354a | A | G | missense_variant |
| 11 | 50960266 | Zfp354a | G | A | missense_variant |
| 11 | 50960280 | Zfp354a | T | C | missense_variant |
| 11 | 50960297 | Zfp354a | A | G | synonymous_variant |
| 11 | 50960319 | Zfp354a | T | C | synonymous_variant |
| 11 | 51124923 | Gm12569 | T | A | missense_variant |
| 11 | 51125086 | Gm12569 | C | G | synonymous_variant |
| 11 | 51125694 | Gm12569 | C | T | missense_variant |
| 11 | 51449285 | Col23a1 | C | T | synonymous_variant |
| 11 | 51458726 | Col23a1 | G | C | missense_variant |

| | | | | | |
|---|---|---|---|---|---|
| 11 | 51463330 | Col23a1 | T | C | synonymous_variant |
| 11 | 51484751 | Phykpl | A | G | missense_variant |
| 11 | 51484947 | Phykpl | C | T | synonymous_variant |
| 11 | 51495501 | Hnrnpab | G | A | synonymous_variant |
| 11 | 51517578 | Rmnd5b | T | G | synonymous_variant |
| 11 | 51518770 | Rmnd5b | T | C | synonymous_variant |
| 11 | 51536300 | N4bp3 | G | C | synonymous_variant |
| 11 | 51603173 | Sec24a | A | G | synonymous_variant |
| 11 | 58898512 | Obscn | C | A | missense_variant |
| 11 | 62964307 | Tekt3 | T | C | synonymous_variant |
| 11 | 69480467 | Trp53 | - | AGA | inframe_insertion |
| 11 | 72871288 | Atp2a3 | C | T | synonymous_variant |
| 11 | 77864755 | Sez6 | G | C | missense_variant |
| 11 | 93988255 | Spag9 | G | A | synonymous_variant |
| 11 | 99663539 | Gm11595 | G | A | missense_variant |
| 11 | 103111928 | Map3k14 | G | A | stop_gained |
| 11 | 106964996 | Bptf | T | G | missense_variant |
| 11 | 117665141 | Tmc6 | G | A | synonymous_variant |
| 11 | 119219314 | Card14 | G | A | missense_variant |
| 12 | 4030473 | Efr3b | G | T | missense_variant |
| 12 | 31351088 | Lamb1 | G | A | stop_gained |
| 12 | 35155170 | Snx13 | G | A | synonymous_variant |
| 12 | 36169096 | Bzw2 | G | A | missense_variant |
| 12 | 37448726 | Agmo | A | C | missense_variant |
| 12 | 51702219 | Strn3 | G | A | synonymous_variant |
| 12 | 61886900 | Lrfn5 | C | A | missense_variant |
| 12 | 79113956 | Plekhh1 | C | A | missense_variant |
| 12 | 80624202 | Galnt16 | G | A | synonymous_variant |
| 12 | 98826318 | Eml5 | G | A | missense_variant |
| 12 | 102453464 | Golga5 | C | T | missense_variant |
| 12 | 104071546 | Serpina5 | A | C | missense_variant |
| 12 | 106029054 | Vrk1 | G | A | missense_variant |
| 12 | 108327907 | Cyp46a1 | G | A | synonymous_variant |
| 12 | 110774691 | Mok | T | G | missense_variant |
| 12 | 112461442 | 30016L24F | G | A | missense_variant |
| 12 | 113025346 | Pacs2 | C | T | synonymous_variant |
| 13 | 8747743 | Adarb2 | T | A | missense_variant |
| 13 | 9583373 | Dip2c | C | T | synonymous_variant |
| 13 | 13823429 | Lyst | G | A | missense_variant |
| 13 | 21652473 | Nkapl | C | G | missense_variant |
| 13 | 21663362 | Zkscan4 | C | T | missense_variant |
| 13 | 23036267 | Vmn1r211 | C | T | synonymous_variant |
| 13 | 32311011 | Gmds | A | G | synonymous_variant |
| 13 | 32311059 | Gmds | A | G | synonymous_variant |
| 13 | 33190624 | Serpinb9 | G | A | synonymous_variant |
| 13 | 33213469 | Serpinb9b | G | A | missense_variant |
| 13 | 33222050 | Serpinb9b | T | C | synonymous_variant |
| 13 | 33222101 | Serpinb9b | C | T | synonymous_variant |
| 13 | 33340448 | Serpinb9c | G | A | missense_variant |
| 13 | 34212109 | Ripk1 | C | T | missense_variant |
| 13 | 34234320 | Bphl | G | A | synonymous_variant |
| 13 | 34258637 | Tubb2a | C | T | synonymous_variant |
| 13 | 34311631 | Tubb2b | A | G | synonymous_variant |
| 13 | 34381805 | Slc22a23 | A | G | synonymous_variant |
| 13 | 35140863 | Eci3 | T | C | synonymous_variant |
| 13 | 35143905 | Eci3 | T | C | synonymous_variant |

| | | | | | |
|---|---|---|---|---|---|
| 13 | 36040875 | Cdyl | G | A | synonymous_variant |
| 13 | 36042073 | Cdyl | G | A | synonymous_variant |
| 13 | 36042187 | Cdyl | A | G | synonymous_variant |
| 13 | 36080779 | Rpp40 | A | G | synonymous_variant |
| 13 | 36080839 | Rpp40 | G | A | synonymous_variant |
| 13 | 36085294 | Rpp40 | G | T | missense_variant |
| 13 | 36086048 | Rpp40 | G | A | synonymous_variant |
| 13 | 36086072 | Rpp40 | T | C | synonymous_variant |
| 13 | 36088427 | Rpp40 | C | T | synonymous_variant |
| 13 | 36088497 | Rpp40 | C | T | missense_variant |
| 13 | 36088517 | Rpp40 | A | C | synonymous_variant |
| 13 | 36090289 | Rpp40 | C | T | synonymous_variant |
| 13 | 36152722 | Ppp1r3g | T | C | synonymous_variant |
| 13 | 36152738 | Ppp1r3g | C | A | synonymous_variant |
| 13 | 36153110 | Ppp1r3g | C | T | missense_variant |
| 13 | 36153118 | Ppp1r3g | T | C | synonymous_variant |
| 13 | 36153265 | Ppp1r3g | A | G | synonymous_variant |
| 13 | 36153277 | Ppp1r3g | C | T | synonymous_variant |
| 13 | 36153346 | Ppp1r3g | T | C | synonymous_variant |
| 13 | 36153365 | Ppp1r3g | G | C | missense_variant |
| 13 | 36153433 | Ppp1r3g | C | G | synonymous_variant |
| 13 | 36721314 | Fars2 | C | G | synonymous_variant |
| 13 | 37172868 | F13a1 | G | T | missense_variant |
| 13 | 38171535 | Ssr1 | T | C | synonymous_variant |
| 13 | 38207025 | Cage1 | G | A | synonymous_variant |
| 13 | 38207298 | Cage1 | C | T | synonymous_variant |
| 13 | 38207303 | Cage1 | T | C | missense_variant |
| 13 | 38207346 | Cage1 | G | A | synonymous_variant |
| 13 | 38209430 | Cage1 | C | G | synonymous_variant |
| 13 | 38209463 | Cage1 | T | C | synonymous_variant |
| 13 | 38209592 | Cage1 | T | C | synonymous_variant |
| 13 | 38209598 | Cage1 | G | A | synonymous_variant |
| 13 | 38209725 | Cage1 | C | T | missense_variant |
| 13 | 38212113 | Cage1 | C | T | synonymous_variant |
| 13 | 38212122 | Cage1 | C | T | synonymous_variant |
| 13 | 38212142 | Cage1 | C | T | missense_variant |
| 13 | 38212149 | Cage1 | C | T | synonymous_variant |
| 13 | 38224109 | Riok1 | C | T | synonymous_variant |
| 13 | 38234880 | Riok1 | C | T | synonymous_variant |
| 13 | 38244053 | Riok1 | T | C | synonymous_variant |
| 13 | 38244057 | Riok1 | A | G | missense_variant |
| 13 | 38352648 | Dsp | A | G | synonymous_variant |
| 13 | 38369630 | Dsp | A | G | synonymous_variant |
| 13 | 38375712 | Dsp | A | G | missense_variant |
| 13 | 38376110 | Dsp | G | A | synonymous_variant |
| 13 | 38376980 | Dsp | G | A | synonymous_variant |
| 13 | 38377079 | Dsp | C | T | synonymous_variant |
| 13 | 38378905 | Dsp | T | C | synonymous_variant |
| 13 | 38380552 | Dsp | A | T | synonymous_variant |
| 13 | 38380847 | Dsp | T | C | synonymous_variant |
| 13 | 38381140 | Dsp | A | G | synonymous_variant |
| 13 | 38381446 | Dsp | T | G | synonymous_variant |
| 13 | 40157528 | Ofcc1 | A | G | missense_variant |
| 13 | 40296266 | Ofcc1 | C | T | missense_variant |
| 13 | 40408944 | Ofcc1 | A | G | synonymous_variant |
| 13 | 40408971 | Ofcc1 | G | A | synonymous_variant |

| | | | | | |
|---|---|---|---|---|---|
| 13 | 40408987 | Ofcc1 | G | A | missense_variant |
| 13 | 40434008 | Ofcc1 | G | T | missense_variant |
| 13 | 40874767 | Tfap2a | G | A | synonymous_variant |
| 13 | 41014240 | Gcnt2 | A | C | missense_variant |
| 13 | 41256233 | Gcm2 | C | T | stop_retained_variant |
| 13 | 41256435 | Gcm2 | A | G | missense_variant |
| 13 | 41256550 | Gcm2 | C | T | missense_variant |
| 13 | 41256575 | Gcm2 | G | A | synonymous_variant |
| 13 | 41256621 | Gcm2 | C | G | missense_variant |
| 13 | 41256678 | Gcm2 | A | G | missense_variant |
| 13 | 41256858 | Gcm2 | T | A | missense_variant |
| 13 | 41257019 | Gcm2 | A | G | synonymous_variant |
| 13 | 41257027 | Gcm2 | AG | GA | missense_variant |
| 13 | 41258055 | Gcm2 | C | T | missense_variant |
| 13 | 41258974 | Gcm2 | T | C | synonymous_variant |
| 13 | 41259207 | Gcm2 | A | G | synonymous_variant |
| 13 | 41288637 | Sycp2l | A | G | missense_variant |
| 13 | 41296974 | Sycp2l | A | G | missense_variant |
| 13 | 49066553 | Fam120a | C | T | missense_variant |
| 13 | 54733696 | Nop16 | G | A | synonymous_variant |
| 13 | 55599147 | Grk6 | C | T | missense_variant |
| 13 | 59863260 | Spata31d1b | A | G | missense_variant |
| 13 | 69648079 | Tent4a | G | A | missense_variant |
| 13 | 69958152 | Med10 | G | A | missense_variant |
| 13 | 73710479 | Slc6a3 | T | C | missense_variant |
| 13 | 73944888 | Slc12a7 | C | T | missense_variant,splice_region_variant |
| 13 | 92712102 | Spz1 | G | A | synonymous_variant |
| 13 | 93200087 | Cmya5 | C | G | missense_variant |
| 13 | 94576825 | Ap3b1 | G | A | synonymous_variant |
| 13 | 100298417 | Naip2 | A | T | missense_variant |
| 14 | 6191862 | Ngly1 | C | T | missense_variant |
| 14 | 10327267 | Ptprg | C | T | missense_variant |
| 14 | 14550541 | Flnb | C | T | missense_variant |
| 14 | 20406480 | Fam149b | C | T | missense_variant |
| 14 | 20716090 | Synpo2l | G | A | missense_variant |
| 14 | 24215387 | Dlg5 | C | T | synonymous_variant |
| 14 | 26159833 | Slmap | C | T | splice_donor_variant |
| 14 | 30617098 | Itih4 | G | A | synonymous_variant |
| 14 | 30947890 | Sema3g | G | A | synonymous_variant |
| 14 | 31000040 | Dnah1 | C | T | synonymous_variant |
| 14 | 31336196 | Hacl1 | C | T | stop_gained |
| 14 | 32871527 | Wdfy4 | C | T | missense_variant |
| 14 | 33677751 | Rbp3 | C | T | synonymous_variant |
| 14 | 33959492 | Shld2 | G | A | missense_variant |
| 14 | 43117079 | Gm8126 | C | T | synonymous_variant |
| 14 | 43844434 | Gm10375 | C | T | synonymous_variant |
| 14 | 45403227 | Txndc16 | C | T | missense_variant |
| 14 | 47780322 | Atg14 | C | T | stop_gained |
| 14 | 48737529 | Tmem260 | GG | AC | missense_variant |
| 14 | 50272629 | Olfr725 | G | A | missense_variant |
| 14 | 50600334 | Tlr11 | C | T | synonymous_variant |
| 14 | 55781986 | Pck2 | C | T | missense_variant |
| 14 | 55887909 | Tssk4 | C | T | missense_variant |
| 14 | 70785478 | Reep4 | C | T | synonymous_variant |
| 14 | 75323584 | Lrrc63 | C | T | missense_variant |
| 14 | 98153250 | Dach1 | C | T | missense_variant |

| | | | | | |
|---|---|---|---|---|---|
| 14 | 103441931 | Mycbp2 | C | T | missense_variant |
| 14 | 103520123 | Mycbp2 | C | T | synonymous_variant |
| 14 | 118202183 | Gpc6 | G | A | synonymous_variant |
| 14 | 118385669 | Gpr180 | A | G | synonymous_variant |
| 14 | 118472913 | Sox21 | C | T | synonymous_variant |
| 14 | 118504795 | Gm9376 | T | C | missense_variant |
| 14 | 118504932 | Gm9376 | T | C | synonymous_variant |
| 14 | 118505001 | Gm9376 | C | T | synonymous_variant |
| 14 | 118835036 | Abcc4 | G | A | missense_variant |
| 14 | 118836747 | Abcc4 | G | A | synonymous_variant |
| 14 | 118849189 | Abcc4 | A | G | synonymous_variant |
| 14 | 118869516 | Abcc4 | G | A | synonymous_variant |
| 14 | 118869528 | Abcc4 | G | A | synonymous_variant |
| 14 | 119099235 | Cldn10 | T | C | synonymous_variant |
| 14 | 119099428 | Cldn10 | T | C | synonymous_variant |
| 14 | 119111109 | Cldn10 | C | T | synonymous_variant |
| 14 | 119111636 | Cldn10 | C | T | missense_variant |
| 14 | 119111686 | Cldn10 | A | G | missense_variant |
| 14 | 119144316 | Dzip1 | T | C | synonymous_variant |
| 14 | 119266433 | Uggt2 | C | T | missense_variant |
| 14 | 121792575 | Dock9 | C | T | missense_variant |
| 15 | 4971033 | Mroh2b | G | T | stop_gained |
| 15 | 6446419 | Dab2 | G | A | missense_variant |
| 15 | 10479178 | Brix1 | G | A | missense_variant |
| 15 | 10523705 | Ttc23l | G | A | missense_variant |
| 15 | 32628335 | Sema5a | G | A | stop_gained |
| 15 | 37927623 | Rrm2b | G | A | missense_variant |
| 15 | 37975878 | Ubr5 | G | A | missense_variant |
| 15 | 38042081 | Ubr5 | G | A | synonymous_variant |
| 15 | 39341977 | Rims2 | T | C | synonymous_variant |
| 15 | 57114296 | Slc22a22 | A | T | missense_variant |
| 15 | 57114353 | Slc22a22 | T | C | missense_variant |
| 15 | 57684841 | Zhx2 | T | C | missense_variant |
| 15 | 57685118 | Zhx2 | C | T | synonymous_variant |
| 15 | 57685643 | Zhx2 | G | C | synonymous_variant |
| 15 | 57685790 | Zhx2 | A | C | synonymous_variant |
| 15 | 57685799 | Zhx2 | C | G | synonymous_variant |
| 15 | 57685820 | Zhx2 | G | A | synonymous_variant |
| 15 | 57685838 | Zhx2 | T | C | synonymous_variant |
| 15 | 57685901 | Zhx2 | T | C | synonymous_variant |
| 15 | 57685943 | Zhx2 | G | A | synonymous_variant |
| 15 | 57685970 | Zhx2 | G | A | synonymous_variant |
| 15 | 57686021 | Zhx2 | C | T | synonymous_variant |
| 15 | 57686279 | Zhx2 | A | G | synonymous_variant |
| 15 | 57686727 | Zhx2 | A | C | missense_variant |
| 15 | 73202721 | Ptk2 | G | A | synonymous_variant |
| 15 | 76582745 | Gpt | G | A | synonymous_variant |
| 15 | 77522207 | Apol11b | A | C | missense_variant |
| 15 | 78139005 | Ncf4 | G | A | missense_variant |
| 15 | 79802578 | Cbx7 | G | A | missense_variant |
| 15 | 82699824 | Tcf20 | G | A | synonymous_variant |
| 15 | 85124918 | Fbln1 | C | T | synonymous_variant |
| 15 | 86028886 | Cerk | G | A | missense_variant |
| 15 | 89409403 | Shank3 | G | A | synonymous_variant |
| 15 | 92667504 | Pdzrn4 | A | G | missense_variant |
| 15 | 97144392 | Amigo2 | G | A | missense_variant |

| | | | | | |
|---|---|---|---|---|---|
| 15 | 98847997 | Tuba1a | A | G | missense_variant |
| 15 | 99127186 | Kcnh3 | G | A | splice_acceptor_variant |
| 15 | 99526514 | Racgap1 | C | T | splice_donor_variant |
| 15 | 101768335 | Krt77 | G | A | synonymous_variant |
| 15 | 102381833 | Pcbp2 | G | A | missense_variant |
| 15 | 102409902 | Map3k12 | G | A | synonymous_variant |
| 15 | 102618927 | Calcoco1 | G | A | synonymous_variant |
| 15 | 102863471 | Hoxc11 | T | C | missense_variant |
| 15 | 103436083 | Pde1b | G | A | stop_gained |
| 16 | 14525088 | Snai2 | C | A | missense_variant |
| 16 | 17902542 | Prodh | A | G | synonymous_variant |
| 16 | 31706942 | Meltf | T | C | synonymous_variant |
| 16 | 35677394 | Parp14 | G | C | missense_variant |
| 16 | 44025421 | Usf3 | C | G | missense_variant |
| 16 | 78131164 | Cxadr | C | T | synonymous_variant |
| 16 | 91454050 | Son | A | T | missense_variant |
| 17 | 3566301 | Tiam2 | C | T | missense_variant |
| 17 | 12833646 | Slc22a2 | G | A | missense_variant |
| 17 | 13194287 | Wtap | T | C | synonymous_variant |
| 17 | 14898954 | Thbs2 | G | A | synonymous_variant |
| 17 | 17259270 | Gm51425 | G | A | missense_variant |
| 17 | 18389181 | Vmn2r92 | G | T | missense_variant |
| 17 | 21240335 | Zfp160 | G | A | missense_variant,splice_region_variant |
| 17 | 23603858 | Vmn2r116 | A | - | frameshift_variant |
| 17 | 24035041 | Srrm2 | [illegible]CAGGAG | - | inframe_deletion |
| 17 | 24532263 | Abca17 | G | A | missense_variant |
| 17 | 24822252 | Tsc2 | C | G | missense_variant |
| 17 | 24823352 | Tsc2 | G | A | missense_variant |
| 17 | 25541191 | Prss29 | G | A | missense_variant |
| 17 | 26426279 | Rgs11 | G | A | missense_variant |
| 17 | 27364187 | Ip6k3 | G | A | missense_variant |
| 17 | 27411916 | Lemd2 | G | A | missense_variant |
| 17 | 27657696 | Grm4 | G | T | synonymous_variant |
| 17 | 28583332 | Tulp1 | C | T | synonymous_variant |
| 17 | 29745474 | Tmem217 | A | G | synonymous_variant |
| 17 | 34786547 | Notch4 | G | A | missense_variant |
| 17 | 34907865 | Tnxb | G | A | missense_variant |
| 17 | 35220600 | Vars | G | A | missense_variant |
| 17 | 35230454 | Vars | G | A | synonymous_variant |
| 17 | 35368323 | Prrc2a | G | A | missense_variant |
| 17 | 35439932 | Nfkbil1 | G | A | stop_gained |
| 17 | 35835446 | Cchcr1 | G | A | missense_variant |
| 17 | 36166028 | Mdc1 | C | - | frameshift_variant |
| 17 | 36198362 | Dhx16 | G | A | synonymous_variant |
| 17 | 36982679 | H2-M1 | G | A | synonymous_variant |
| 17 | 37194122 | Trim40 | G | A | synonymous_variant |
| 17 | 37536712 | Olfr96 | G | A | missense_variant |
| 17 | 46486241 | Polh | G | A | synonymous_variant |
| 17 | 46569440 | Tjap1 | G | A | missense_variant |
| 17 | 47119416 | Bicral | G | A | missense_variant |
| 17 | 47284907 | Ubr2 | G | A | missense_variant |
| 17 | 47711335 | Guca1a | T | C | missense_variant |
| 17 | 53212123 | Kcnh8 | G | A | synonymous_variant |
| 17 | 56412915 | Plin4 | G | A | missense_variant |
| 17 | 56578233 | Ticam1 | G | A | synonymous_variant |
| 17 | 57608861 | Vav1 | G | A | missense_variant,splice_region_variant |

| | | | | | |
|---|---|---|---|---|---|
| 17 | 66426096 | Washc1 | G | A | missense_variant |
| 17 | 68055058 | Lama1 | A | C | missense_variant |
| 17 | 68059878 | Lama1 | G | A | missense_variant |
| 17 | 68106112 | Lama1 | G | A | missense_variant |
| 17 | 72036325 | Togaram2 | G | A | synonymous_variant |
| 17 | 74205404 | Xdh | C | G | splice_region_variant,synonymous_variant |
| 17 | 74948744 | Birc6 | G | A | missense_variant |
| 17 | 75106317 | Ttc27 | C | T | missense_variant |
| 17 | 79249618 | Ndufaf7 | G | A | missense_variant |
| 17 | 85421239 | Camkmt | G | A | missense_variant |
| 17 | 86446630 | Srbd1 | G | A | missense_variant |
| 17 | 88866065 | Ppp1r21 | G | A | missense_variant |
| 17 | 95046919 | Mettl4 | G | A | missense_variant |
| 18 | 5767024 | Zeb1 | C | T | missense_variant |
| 18 | 9280850 | Gjd4 | C | A | missense_variant |
| 18 | 12344707 | Npc1 | C | T | missense_variant |
| 18 | 13978408 | Zfp521 | C | T | synonymous_variant |
| 18 | 16757248 | Cdh2 | G | A | synonymous_variant |
| 18 | 16757314 | Cdh2 | A | G | synonymous_variant |
| 18 | 16757844 | Cdh2 | C | T | synonymous_variant |
| 18 | 16757922 | Cdh2 | C | T | synonymous_variant |
| 18 | 20176789 | Dsc2 | C | A | synonymous_variant |
| 18 | 20414962 | Dsg1c | C | A | synonymous_variant |
| 18 | 20643462 | Dsg3 | T | C | missense_variant |
| 18 | 20652841 | Dsg3 | G | C | missense_variant |
| 18 | 20653541 | Dsg3 | C | A | missense_variant,splice_region_variant |
| 18 | 20657714 | Dsg3 | T | C | synonymous_variant |
| 18 | 20657738 | Dsg3 | G | C | missense_variant |
| 18 | 20658212 | Dsg3 | A | G | synonymous_variant |
| 18 | 20658227 | Dsg3 | T | C | synonymous_variant |
| 18 | 20658251 | Dsg3 | A | G | synonymous_variant |
| 18 | 20660670 | Dsg3 | T | C | synonymous_variant |
| 18 | 20660706 | Dsg3 | T | C | synonymous_variant |
| 18 | 20662035 | Dsg3 | T | C | synonymous_variant |
| 18 | 20662056 | Dsg3 | G | A | synonymous_variant |
| 18 | 20662122 | Dsg3 | T | C | synonymous_variant |
| 18 | 20672805 | Dsg3 | T | C | synonymous_variant |
| 18 | 20706566 | Dsg2 | C | T | synonymous_variant |
| 18 | 20729024 | Dsg2 | G | A | synonymous_variant |
| 18 | 20961622 | Trappc8 | A | G | synonymous_variant |
| 18 | 20980197 | Trappc8 | C | T | synonymous_variant |
| 18 | 20996549 | Trappc8 | T | C | synonymous_variant |
| 18 | 37624286 | Pcdhb18 | C | T | missense_variant |
| 18 | 37882224 | Pcdhga10 | G | A | missense_variant |
| 18 | 38387052 | Dele1 | C | T | synonymous_variant |
| 18 | 57426962 | Megf10 | C | T | synonymous_variant |
| 18 | 58070829 | Slc12a2 | C | T | synonymous_variant |
| 18 | 58159588 | Fbn2 | G | A | synonymous_variant |
| 18 | 58168971 | Fbn2 | T | C | synonymous_variant |
| 18 | 58168974 | Fbn2 | A | G | synonymous_variant |
| 18 | 58168983 | Fbn2 | G | A | synonymous_variant |
| 18 | 58169007 | Fbn2 | A | G | synonymous_variant |
| 18 | 58170071 | Fbn2 | A | G | synonymous_variant |
| 18 | 58170110 | Fbn2 | C | T | synonymous_variant |
| 18 | 58171294 | Fbn2 | A | G | synonymous_variant |
| 18 | 58171306 | Fbn2 | G | A | synonymous_variant |

| | | | | | |
|---|---|---|---|---|---|
| 18 | 58171321 | Fbn2 | T | C | synonymous_variant |
| 18 | 58177356 | Fbn2 | A | G | synonymous_variant |
| 18 | 58178453 | Fbn2 | G | A | synonymous_variant |
| 18 | 58192459 | Fbn2 | G | A | synonymous_variant |
| 18 | 58196595 | Fbn2 | A | G | synonymous_variant |
| 18 | 58196626 | Fbn2 | C | T | missense_variant |
| 18 | 58202207 | Fbn2 | T | C | synonymous_variant |
| 18 | 58204941 | Fbn2 | G | A | synonymous_variant |
| 18 | 58217656 | Fbn2 | G | A | synonymous_variant |
| 18 | 58229013 | Fbn2 | C | T | synonymous_variant |
| 18 | 58237081 | Fbn2 | T | C | missense_variant |
| 18 | 58246392 | Fbn2 | A | G | synonymous_variant |
| 18 | 58247574 | Fbn2 | G | T | missense_variant |
| 18 | 59144167 | Adamts19 | G | A | synonymous_variant |
| 18 | 59165968 | Adamts19 | G | A | synonymous_variant |
| 18 | 59309239 | Chsy3 | T | C | missense_variant |
| 18 | 59309435 | Chsy3 | A | G | synonymous_variant |
| 18 | 60433011 | 30016B08R | - | AAC | inframe_insertion |
| 18 | 60840940 | Ndst1 | T | C | synonymous_variant |
| 18 | 60964612 | Tcof1 | A | G | synonymous_variant |
| 18 | 60971838 | Tcof1 | T | C | synonymous_variant |
| 18 | 60976372 | Tcof1 | G | A | missense_variant |
| 18 | 61243327 | Csf1r | G | T | missense_variant |
| 18 | 61417124 | Pde6a | C | T | synonymous_variant |
| 18 | 61515567 | Ppargc1b | G | A | synonymous_variant |
| 18 | 61638830 | Arhgef37 | G | C | missense_variant |
| 18 | 61870668 | Afap1l1 | G | A | missense_variant |
| 18 | 62004710 | Ablim3 | G | A | synonymous_variant |
| 18 | 62086234 | Sh3tc2 | T | C | synonymous_variant |
| 18 | 62094061 | Sh3tc2 | G | A | synonymous_variant |
| 18 | 62101172 | Sh3tc2 | C | T | synonymous_variant |
| 18 | 62106370 | Sh3tc2 | T | C | missense_variant |
| 18 | 62144917 | Sh3tc2 | A | C | synonymous_variant |
| 18 | 62311897 | Adrb2 | G | A | synonymous_variant |
| 18 | 62638574 | Fbxo38 | G | T | synonymous_variant |
| 18 | 65439810 | Alpk2 | C | T | missense_variant |
| 18 | 66992149 | Mc4r | A | G | synonymous_variant |
| 18 | 66992566 | Mc4r | G | A | synonymous_variant |
| 18 | 66993013 | Mc4r | C | T | synonymous_variant |
| 18 | 66993039 | Mc4r | C | T | missense_variant |
| 18 | 67370609 | Mppe1 | A | G | synonymous_variant |
| 18 | 67534387 | Tubb6 | G | A | synonymous_variant |
| 18 | 67534396 | Tubb6 | A | G | synonymous_variant |
| 18 | 67534726 | Tubb6 | T | C | synonymous_variant |
| 18 | 67535302 | Tubb6 | T | C | synonymous_variant |
| 18 | 67542570 | Afg3l2 | G | A | synonymous_variant |
| 18 | 67542600 | Afg3l2 | G | A | synonymous_variant |
| 18 | 67547193 | Afg3l2 | A | G | synonymous_variant |
| 18 | 67554078 | Afg3l2 | C | T | synonymous_variant |
| 18 | 67554081 | Afg3l2 | C | T | synonymous_variant |
| 18 | 67559058 | Afg3l2 | C | T | synonymous_variant |
| 18 | 67559064 | Afg3l2 | C | T | synonymous_variant |
| 18 | 67564741 | Afg3l2 | A | G | synonymous_variant |
| 18 | 67575912 | Afg3l2 | T | C | synonymous_variant |
| 18 | 67606062 | Prelid3a | T | C | synonymous_variant |
| 18 | 67652398 | Spire1 | T | C | synonymous_variant |

| | | | | | |
|---|---|---|---|---|---|
| 18 | 67678781 | Spire1 | C | A | synonymous_variant |
| 18 | 67685653 | Spire1 | G | A | synonymous_variant |
| 18 | 67756216 | Cep76 | C | T | synonymous_variant |
| 18 | 67756282 | Cep76 | C | T | synonymous_variant |
| 18 | 67921804 | Seh1l | C | T | synonymous_variant |
| 18 | 67951877 | Cep192 | T | C | missense_variant |
| 18 | 67962928 | Cep192 | T | C | missense_variant |
| 18 | 67967578 | Cep192 | T | C | synonymous_variant |
| 18 | 67968015 | Cep192 | C | T | missense_variant |
| 18 | 67971028 | Cep192 | C | A | missense_variant |
| 18 | 67974877 | Cep192 | A | G | missense_variant |
| 18 | 67983530 | Cep192 | T | C | missense_variant |
| 18 | 67987278 | Cep192 | CA | TG | missense_variant |
| 18 | 67989284 | Cep192 | G | A | synonymous_variant |
| 18 | 67991169 | Cep192 | A | C | missense_variant |
| 18 | 67995572 | Cep192 | A | G | missense_variant |
| 18 | 67999550 | Cep192 | T | C | missense_variant |
| 18 | 68014396 | Cep192 | C | T | synonymous_variant |
| 18 | 68239707 | Ldlrad4 | T | C | synonymous_variant |
| 18 | 68472004 | Mc5r | T | A | missense_variant |
| 18 | 69770026 | Tcf4 | T | C | synonymous_variant |
| 18 | 69784342 | Tcf4 | A | G | synonymous_variant |
| 18 | 69811099 | Tcf4 | C | T | synonymous_variant |
| 18 | 70600483 | 30503L19R | G | A | synonymous_variant |
| 18 | 70609250 | Stard6 | C | G | missense_variant |
| 18 | 70642392 | Poli | C | T | missense_variant |
| 18 | 70659638 | Poli | A | G | synonymous_variant |
| 18 | 71432466 | Dcc | T | C | synonymous_variant |
| 18 | 71454180 | Dcc | A | G | synonymous_variant |
| 18 | 71454196 | Dcc | G | T | synonymous_variant |
| 18 | 71454208 | Dcc | A | G | synonymous_variant |
| 18 | 71469030 | Dcc | A | G | synonymous_variant |
| 18 | 71469081 | Dcc | A | G | synonymous_variant |
| 18 | 71469144 | Dcc | C | T | synonymous_variant |
| 18 | 71500290 | Dcc | A | G | synonymous_variant |
| 18 | 71553345 | Dcc | T | C | synonymous_variant |
| 18 | 77482126 | Loxhd1 | G | A | missense_variant |
| 18 | 81016832 | Sall3 | C | T | synonymous_variant |
| 19 | 16582362 | Gna14 | G | A | missense_variant |
| 19 | 16653507 | Vps13a | G | A | missense_variant |
| 19 | 29254266 | Jak2 | G | T | missense_variant |
| 19 | 44924048 | Slf2 | G | A | synonymous_variant |
| 19 | 46069772 | Nolc1 | G | A | missense_variant |
| 19 | 53840042 | Rbm20 | G | T | missense_variant |
| 19 | 55214437 | Gucy2g | G | A | missense_variant |
| 19 | 56310283 | Nrap | G | A | missense_variant |
| 19 | 60823384 | Dennd10 | G | A | missense_variant |
| X | 13022718 | Usp9x | C | T | synonymous_variant |
| X | 56147340 | Vgll1 | G | T | missense_variant |
| X | 71544724 | Gabra3 | A | C | missense_variant |
| X | 101750863 | Gm9112 | G | A | missense_variant |
| X | 103899519 | Magee2 | G | A | synonymous_variant |
| X | 134570113 | Tcp11x2 | T | C | missense_variant |
| X | 134744517 | Gprasp2 | C | T | synonymous_variant |
| X | 134977012 | Nxf3 | C | T | splice_region_variant,synonymous_variant |
| X | 135071673 | Tceal8 | T | G | missense_variant |

| | | | | | |
|---|---|---|---|---|---|
| X | 135115184 | Bex1 | T | C | synonymous_variant |
| X | 135115209 | Bex1 | G | A | missense_variant |
| X | 135425702 | Kir3dl1 | C | T | synonymous_variant |
| X | 135425885 | Kir3dl1 | A | C | synonymous_variant |
| X | 135427322 | Kir3dl1 | G | C | missense_variant |
| X | 135430588 | Kir3dl1 | A | G | missense_variant,splice_region_variant |
| X | 135430654 | Kir3dl1 | A | G | missense_variant |
| X | 135434691 | Kir3dl1 | G | A | missense_variant,splice_region_variant |
| X | 135874972 | Tmsb15b1 | T | G | missense_variant |
| X | 135876158 | Tmsb15l | G | A | missense_variant |
| X | 135884271 | Slc25a53 | C | T | synonymous_variant |
| X | 135896217 | Zcchc18 | T | C | synonymous_variant |
| X | 135916035 | Slc25a53 | [illegible]ACAGCA[illegible] | - | frameshift_variant |
| X | 135916053 | Slc25a53 | G | A | synonymous_variant |
| X | 135916081 | Slc25a53 | G | A | missense_variant |
| X | 135963473 | Fam199x | C | G | synonymous_variant |
| X | 135963590 | Fam199x | G | A | synonymous_variant |
| X | 136019402 | Esx1 | A | C | missense_variant |
| X | 162298030 | Grpr | A | C | missense_variant |
| Y | 826391 | Uba1y | G | A | synonymous_variant |

**Table S2. Neoantigen mutations with high affinity to H-2K$^b$ alleles**

| Gene | Mutation | Neoepitope | TPM[a] | VAF[b] | IC50 (MT[c]) |
|---|---|---|---|---|---|
| Ecpas | L1064F | STYKEFCSL | 17.35324 | 0.431579 | 12.8 |
| Tbc1d32 | M1054L | LTVRYGRFLNL | 2.30625 | 0.42069 | 39.6 |
| Rcc1 | S29F | VSHRFHNT | 23.706072 | 0.411765 | 76.3 |
| Ubr2 | A537V | AVFTLQMKL | 11.28678 | 0.333333 | 76.5 |
| Tmem30a | P290S | SAGQYFLNI | 33.6073 | 0.522727 | 86.0 |
| Atr | L2126F | SVFTEHTNRL | 4.406605 | 0.409091 | 118.4 |
| Klhl26 | A154V | VSLVLRHV | 10.831677 | 0.337931 | 125.6 |
| Dennd5b | L338V | ASLLHFVDAPV | 1.152 | 0.336207 | 131.8 |
| Mycbp2 | V1978I | IAILNQKYA | 15.179241 | 0.472603 | 171.0 |
| Bptf | K1284N | RAFSNAVDF | 10.327738 | 0.357542 | 216.1 |
| Ubr5 | P2546S | CSITLNRHVI | 27.878175 | 0.300469 | 225.5 |
| Rgs11 | D224N | SNFYKCEI | 7.363055 | 0.275568 | 232.5 |
| Slc22a15 | S96T | VHFSNSFTTI | 1.712 | 0.339286 | 241.4 |
| Rbbp6 | P1616S | SQLSHSSRL | 18.597982 | 0.392405 | 242.0 |
| Ncor2 | P1737L | LAYLPTAPPL | 29.053076 | 0.330935 | 291.0 |
| Snai2 | P198H | KAFSRHWL | 6.1506 | 0.43 | 291.5 |
| Slc12a7 | L467F | VTTSFIYF | 7.884342 | 0.495726 | 390.0 |
| Ly75 | D1328E | LMWFEKTAL | 7.633376 | 0.485849 | 403.1 |
| Grk6 | T117I | IHEYLSTAPF | 17.70934 | 0.407258 | 406.9 |
| Birc6 | V3064I | TIHMMLQPI | 18.058234 | 0.357988 | 434.7 |
| Pxylp1 | T469I | SSINYYDAC | 1.471476 | 0.466667 | 478.3 |
| Tmem260 | D333H | VHFPGHRWNPV | 4.07349 | 0.284768 | 480.6 |

a: TPM, transcripts per kilobase per million mapped reads;

b: VAF, variant allele frequency;

c: MT, mutation.
